# Supplementary material for: Honey bees (Apis mellifera) modify plant-pollinator network structure, but do not alter wild species’ interactions
Source: PLoS One. 2023 Jul 13;18(7):e0287332. doi: 10.1371/journal.pone.0287332 (PMC10343163; doi:10.1371/journal.pone.0287332)
Supplement: S1 File — (DOCX) [file pone.0287332.s001.docx]

# Supporting information

## Description of network metrics associated with stability

Of the network metrics theoretically related to plant-pollinator network stability, **nestedness** has the strongest association. A highly nested network is one in which interactions of specialists are subsets of the interactions of generalists. In this way, the interactions in the network become highly asymmetrical and organized around a few generalist species [1]. Plant-pollinator networks are generally nested when “binary” networks are considered (presence/absence of interactions only, as opposed to “quantitative” networks that also consider interaction frequencies), and this binary nested structure has been interpreted to minimize interspecific competition and enhance species coexistence, and to arise when new species enter the network where they have minimal competitive load [2]. Binary nestedness increases plant-pollinator network stability [3], and almost all studies that have examined honey bee effects on nestedness concur that honey bees increase binary network nestedness [4–6] when honey bee interactions are included in the network, but the effect of honey bees on nestedness of just the wild pollinator-plant interactions has not been investigated. More recently, [7] showed that although most plant-pollinator networks are nested in their binary structure, they are not quantitatively nested, meaning that less abundant flower species are focused on by different pollinator species, such that niches are less overlapping than appears to be the case in the binary structure. Because we were interested in detecting honey bee effects on this kind of niche partitioning, we used the weighted version, WNODF, of the metric for nestedness, NODF, that has been used in previous studies of the effects of honey bees on nestedness [4–6].

**Modularity** (the extent to which interactions are clumped into semi-separate modules] is thought to decrease stability in plant-pollinator networks [3] and to be decreased by honey bees, because they introduce new links that join previously separate modules [4, 5]; but [8] found the opposite). We calculated modularity only for the full season all taxa and full season without-honey-bees datasets; as it was not significant in either, it was not calculated for other datasets.

Other network metrics are more tenuously linked to stability, such as **connectance** (number of observed interactions over total possible interactions) and **link density** (total number of interactions over the total number of nodes, or the mean number of interactions per species). Both are expected to increase stability [3] and be decreased by honey bees [8-10]. [9] compared effects of honey bees on link density of the total network and effects of honey bees on link density of just wild pollinator interactions, and found that the effect of honey bees on this metric decreased dramatically when the honey bee interactions were not included in the calculation of the metric. Nevertheless, they found a significant but small decrease in link density of wild pollinator interactions as honey bee density increased.

Network metrics of **interaction strength asymmetry** (the asymmetrical strength of one species interaction relative to the reciprocal interaction, averaged across all species pairs in the network) and **interaction evenness** (the uniformity of the interaction frequencies distributed through the network, calculated using Shannon’s evenness index) may also be related to stability. Interaction strength asymmetry has been found to increase with increasing honey bee abundance, as honey bees dominate interactions with wild plants [10]. However, interaction evenness has been found to decrease with increasing honey bee abundance, as the distribution of interactions was skewed toward honey bees [9] and even for plant-pollinator networks excluding honey bees [11], though this study identified taxa into broad groups rather than providing species-level resolution, so network metrics may not be comparable). While interaction strength asymmetry is thought to be stabilizing, interaction evenness may be destabilizing, though its relationship to stability has only been tested in antagonistic networks, not in mutualistic networks [12].

## Justifications for the predictions shown in Fig 1b.

In Fig 1b, we show our predictions as to how each network metric will change as honey bee abundance increases (going from transects far from honey bee hives to transects close to honey bee hives). Because we added honey bees in high enough densities that they would be involved in a large proportion of all plant-flower visitor interactions, for certain network metrics, we anticipated that increasing honey bee abundance would affect the with-honey bee versus without-honey bee networks in different ways (yellow vs. blue lines in Fig 1b).

## Resource overlap metrics

For the resource overlap metrics all of our predictions stem from our hypothesis that introduced honey bees would compete with many other pollinator species, excluding some of these species from some of their floral resources, and narrowing their floral niches. We predicted decreases in **pollinator niche overlap** (a univariate index of similarity in the set of flower species visited by different pollinator species), **plant niche overlap** (univariate similarity in the set of pollinator species visiting each plant species), **generality** (the mean number of plant species visited by each pollinator species), **vulnerability** (the mean number of pollinator species visiting each plant species), and increases in **pollinator functional complementarity** (the multivariate dissimilarity of the set of species that each pollinator species interacts with) and **plant functional complementarity** (multivariate dissimilarity of the set of species that each plant species interacts with) [9].

## Stability-related metrics for networks including honey bees (yellow trend lines in Fig 1b)

Our predictions for the effects of varying honey bee abundance on the set of stability-related metrics are based on the findings of previous studies. We expected networks from transects with higher honey bee abundance to have an increase in **nestedness** (**WNODF**) [4–6, 13, 14], either an increase [8] or a decrease [4, 5] in **modularity**, decreases in **connectance** [8, 10], **link density** [8-10], and **interaction evenness** [9], and either no change [8] or an increase [10] in **interaction strength asymmetry**.

## Stability-related metrics for networks excluding honey bees (blue trend lines in Fig 1b)

With honey bees removed from the network we predicted no change in WNODF and interaction strength asymmetry, an increase in modularity, and decreases in connectance, link density [9], and interaction evenness [11].

## Calculation of network metrics associated with stability

The first network metric for stability, **weighted nestedness** based on overlap and decreasing fill (WNODF, hereinafter referred to as “nestedness”) is a measurement of the degree to which interactions of specialists are subsets of the interactions of generalists [15]. We adopted the methods used by [8] to calculate **modularity** using DIRT_LBA_wb_plus which computes modules with **Newman’s modularity measure** (bipartite package, [16, 17]). We ran 50 trials for each network matrix and retained the output to two decimal places. **Interaction strength asymmetry** describes, on average, how asymmetric the interactions are between interacting species pairs from each trophic level, where strength is the frequency of visits [18]. We used the “Bluethgen” method to calculate this metric [19]. Similarly, **interaction evenness** measures the evenness of the frequency of unique interactions using **Shannon’s evenness** [20, 21]. **Weighted connectance** is a calculation of the proportion of interactions relative to the number of possible interactions in a network, and is therefore calculated by dividing the number of links by the number of cells in the species interaction matrix [16]. Lastly, **link density** measures the mean number of interactions per pollinator species weighted by the average number of interactions of each species [20, 21].

## Rationale for using both SLR and MR models in the statistical analysis

The purpose of using both an SLR and an MR to analyze honey bee effects on each response variable is because it is statistically impossible to separate any correlated effects of honey bee abundance and flower community variables (flower abundance and flower species richness) on our response variables (network metrics). In an SLR, any effect on the response variable possibly attributable to honey bee abundance is attributed fully to honey bee abundance. Conversely, in the MR model any effect on the response variable that is statistically indistinguishable (because of multicollinearity) between an effect due to honey bees or an effect due to flower community variables, is not attributed to honey bees [22]. Thus, the reason to include the SLR model in addition to the more conservative MR model is because the part of the effect of the predictor variables on the response variable that cannot be attributed to solely honey bee abundance, may in reality be due to honey bee abundance, but our methods cannot detect this. So, providing the SLR as well as the MR allows us to see what the maximum possible effect of honey bee abundance was in this study (regression coefficient from the SLR) versus the more conservative but unambiguous effect of honey bee abundance (partial regression coefficient from the MR) [22].

## References

1. Bascompte J, Jordano P, Melian CJ, Olesen JM. The nested assembly of plant-animal mutualistic networks. Proceedings of the National Academy of Sciences. 2003 Aug 5;100(16):9383–7.

2. Bastolla U, Fortuna MA, Pascual-García A, Ferrera A, Luque B, Bascompte J. The architecture of mutualistic networks minimizes competition and increases biodiversity. Nature. 2009;458(7241):1018–20.

3. Thébault E, Fontaine C. Stability of Ecological Communities and the Architecture of Mutualistic and Trophic Networks. Science. 2010 Aug 13;329(5993):853–6.

4. Geslin B, Gauzens B, Baude M, Dajoz I, Fontaine C, Henry M, et al. Massively Introduced Managed Species and Their Consequences for Plant–Pollinator Interactions. Advances in Ecological Research. 2017;57:147–99.

5. Santos, GM, Aguiar CML, Genini J, Martins CF, Zanella FCV, Mello MAR. Invasive Africanized honeybees change the structure of native pollination networks in Brazil. Biol Invasions. 2012 Nov;14(11):2369–78.

6. Giannini TC, Cordeiro GD, Freitas BM, Saraiva AM, Imperatriz-Fonseca VL. The Dependence of Crops for Pollinators and the Economic Value of Pollination in Brazil. Journal of Economic Entomology. 2015 Jun 1;108(3):849–57.

7. Staniczenko PPA, Kopp JC, Allesina S. The ghost of nestedness in ecological networks. Nat Commun. 2013 Jun;4(1):1391.

8. Valido A, Rodríguez-Rodríguez MC, Jordano P. Honeybees disrupt the structure and functionality of plant-pollinator networks. Sci Rep. 2019 Dec;9(1):4711.

9. Magrach A, González-Varo JP, Boiffier M, Vilà M, Bartomeus I. Honeybee spillover reshuffles pollinator diets and affects plant reproductive success. Nat Ecol Evol. 2017 Sep;1(9):1299–307.

10. Aizen MA, Morales CL, Morales JM. Invasive Mutualists Erode Native Pollination Webs. Simberloff D, editor. PLoS Biol [Internet]. 2008 Feb 12 [cited 2021 Aug 1];6(2). Available from: https://dx.plos.org/10.1371/journal.pbio.0060031

11. Ropars L, Dajoz I, Fontaine C, Muratet A, Geslin B. Wild pollinator activity negatively related to honey bee colony densities in urban context. Blenau W, editor. PLoS ONE [Internet]. 2019 Sep 12 [cited 2021 Jul 1];14(9). Available from: https://dx.plos.org/10.1371/journal.pone.0222316

12. Frost CM, Allen WJ, Courchamp F, Jeschke JM, Saul WC, Wardle DA. Using Network Theory to Understand and Predict Biological Invasions. Trends in Ecology and Evolution. 2019;34(9):831–43.

13. Lázaro A, Müller A, Ebmer AW, Dathe HH, Scheuchl E, Schwarz M, et al. Impacts of beekeeping on wild bee diversity and pollination networks in the Aegean Archipelago. Ecography. 2021 Sep;44(9):1353–65.

14. Traveset A, Heleno R, Chamorro S, Vargas P, McMullen CK, Castro-Urgal R, et al. Invaders of pollination networks in the Galápagos Islands: emergence of novel communities. Proc R Soc B. 2013 May 7;280(1758):20123040.

15. Almeida-Neto M, Ulrich W. A straightforward computational approach for measuring nestedness using quantitative matrices. Environmental Modelling and Software. 2011;26(2):173–8.

16. Dormann CF, Gruber B, Frueund J. Introducing the bipartite package: Analysing Ecological Networks. R News. 2008;8(2):8–11.

17. Newman MEJ, Girvan M. Finding and evaluating community structure in networks. Phys Rev E. 2004 Feb 26;69(2):026113.

18. Bascompte J, Jordano P, Olesen JM. Asymmetric coevolutionary networks facilitate biodiversity maintenance. Science. 2006;312(5772):431–3.

19. Blüthgen N. Why network analysis is often disconnected from community ecology: A critique and an ecologist’s guide. Basic and Applied Ecology. 2010 May;11(3):185–95.

20. Bersier LF, Banašek-Richter C, Cattin MF. Quantitative descriptors of food-web matrices. Ecology. 2002 Sep;83(9):2394–407.

21. Tylianakis JM, Tscharntke T, Lewis OT. Habitat modification alters the structure of tropical host-parasitoid food webs. Nature. 2007;445(7124):202–5.

22. Morrissey MB, Ruxton GD. Multiple Regression Is Not Multiple Regressions: The Meaning of Multiple Regression and the Non-Problem of Collinearity. Philosophy, Theory, and Practice in Biology [Internet]. 2018 Jun [cited 2022 Apr 28];10(20220112). Available from: http://hdl.handle.net/2027/spo.16039257.0010.003
