# Supplementary figures and images for: Honey bees (Apis mellifera) modify plant-pollinator network structure, but do not alter wild species’ interactions
Source: PLoS One. 2023 Jul 13;18(7):e0287332. doi: 10.1371/journal.pone.0287332 (PMC10343163; doi:10.1371/journal.pone.0287332)

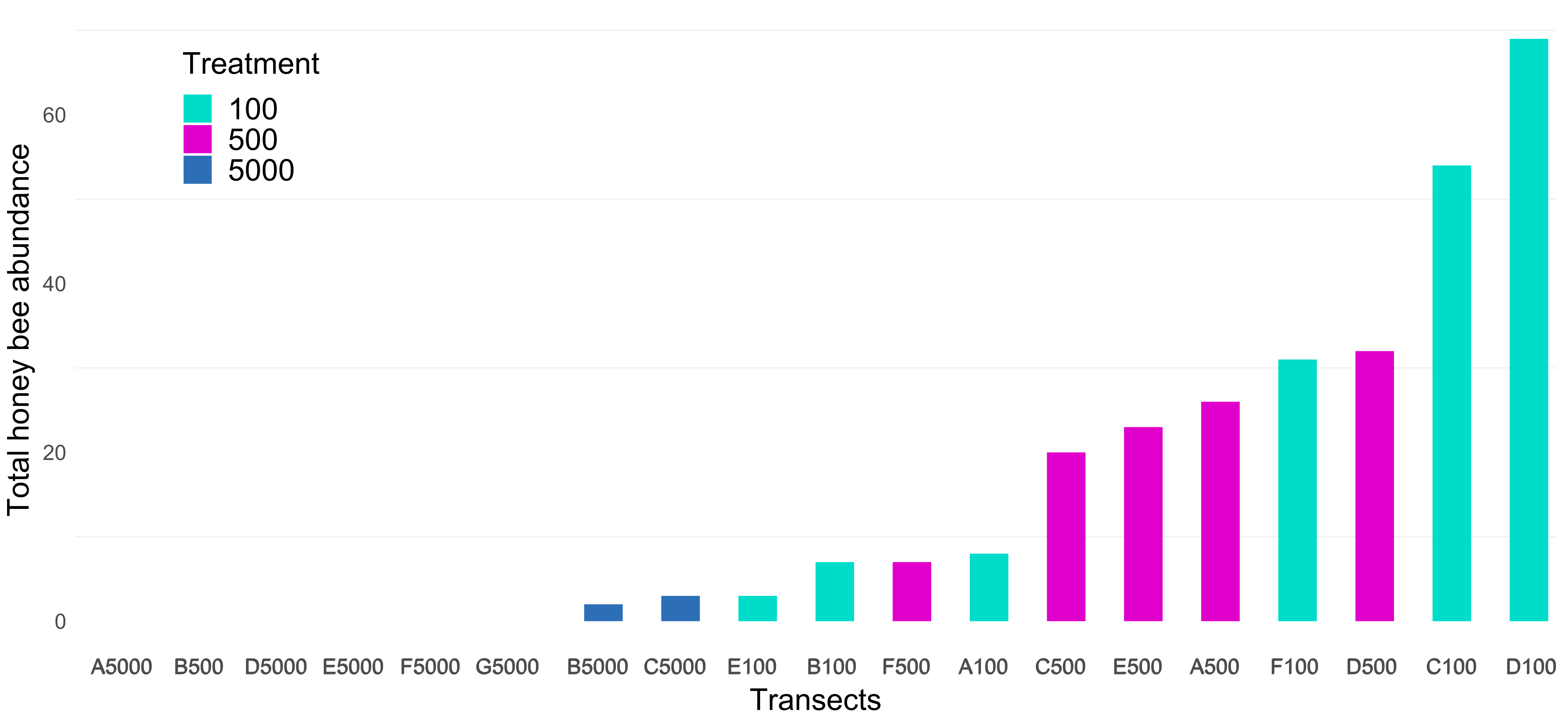

Supplement: S1 Fig — In the transect names, 100 indicates 100 m, 500 indicates 500 m, and 5000 indicates 5000 m distances from hives. (TIFF) [file pone.0287332.s002.tiff]

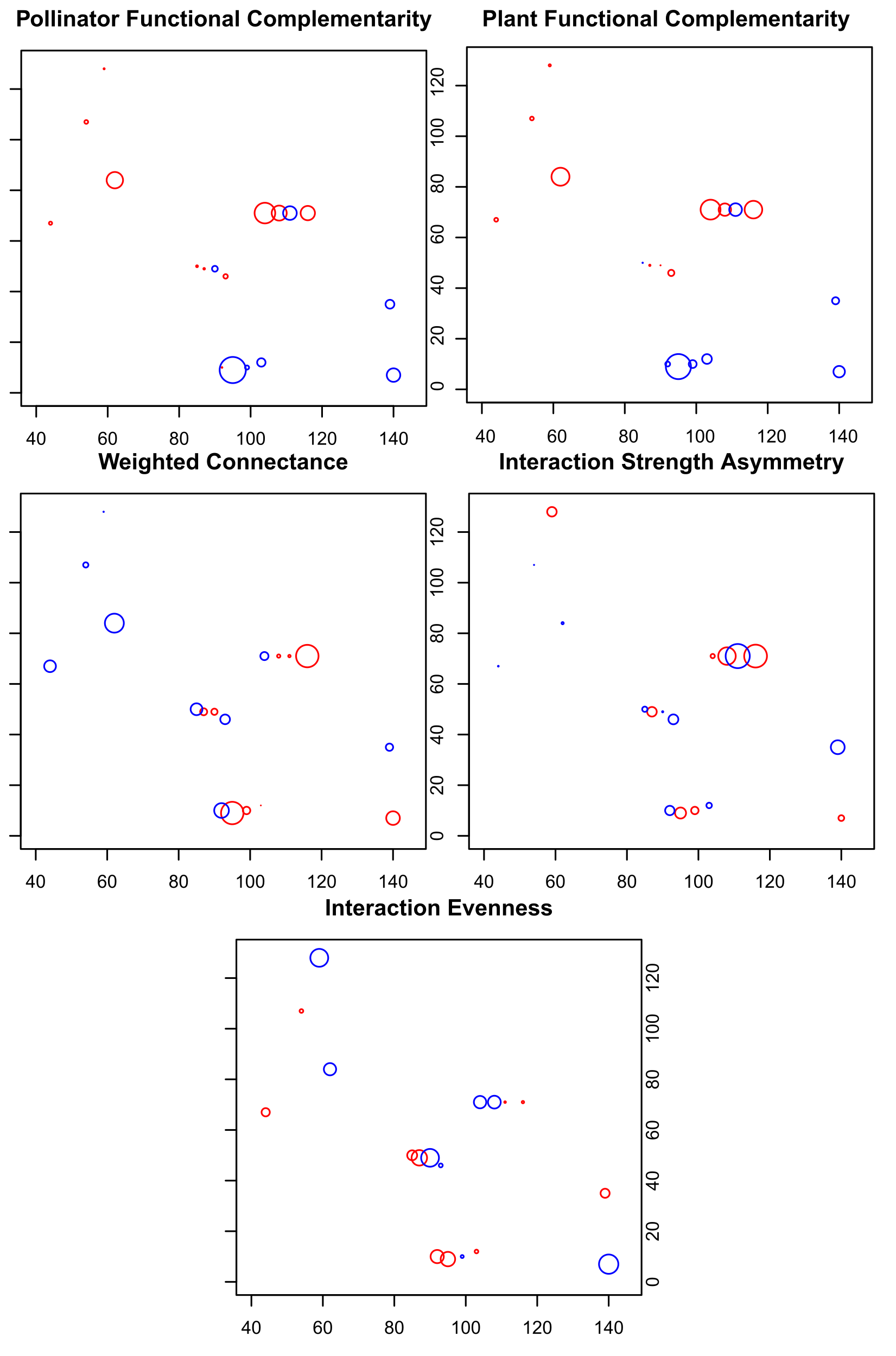

Supplement: S2 Fig — Each panel is a map of the transect locations in space (compare to Fig 2). Circles indicate the residual from each transect, with circle size proportional to residual size (smaller circle = better model fit for that transect). Colour indicates the sign of the residual; blue shows values lower than 0 and red values higher than 0. Circles close together in space having the same colour and size would indicate that spatial autocorrelation might be a problem, in which case a special correlation structure would be likely to be selected during model selection. (TIFF) [file pone.0287332.s003.tiff]

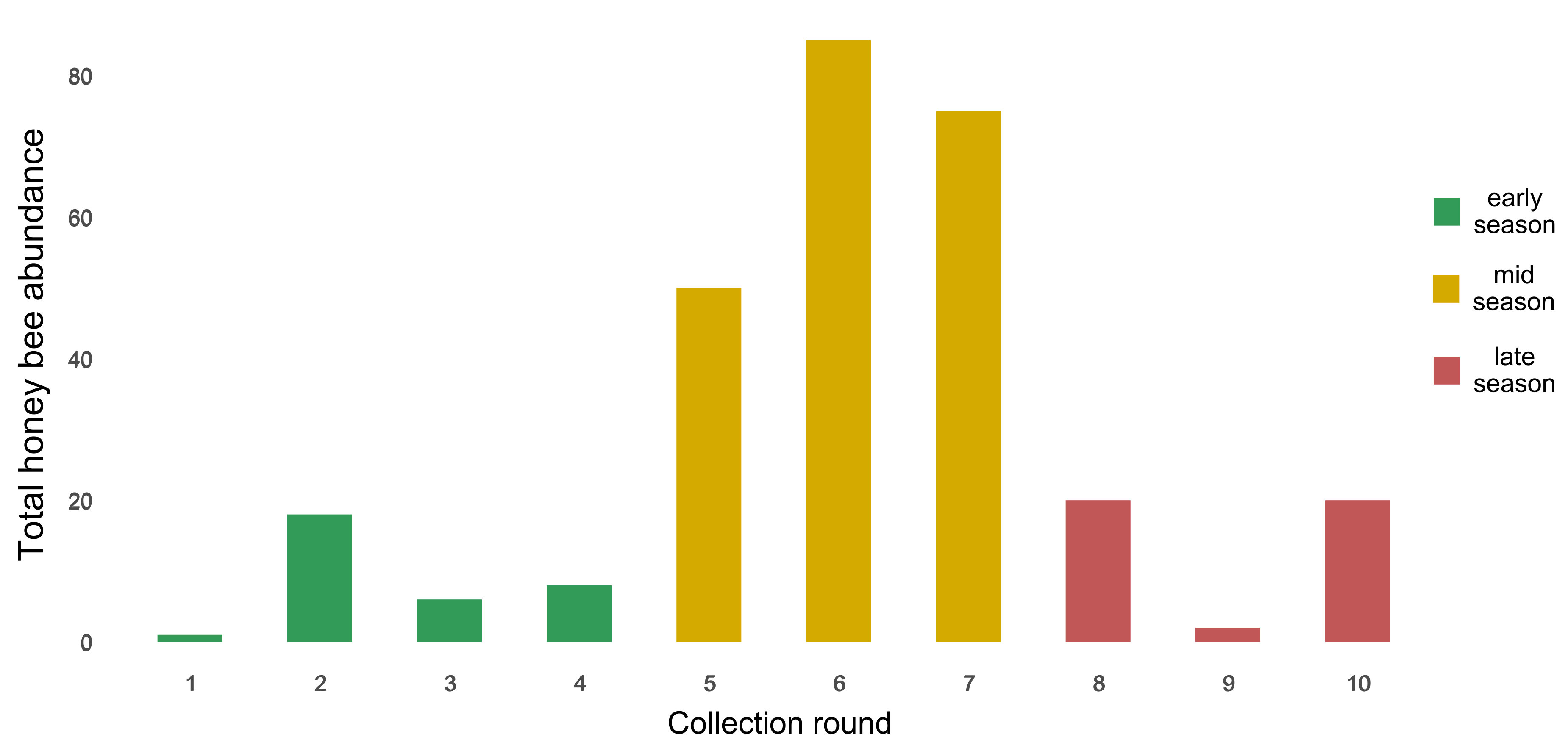

Supplement: S3 Fig — The entire season was split into three: collection rounds 1–4 represented “early” season (May 28th-July 7th), 5–7 represented “mid” season (July 8th-July 31st), and 8–10 represented “late” season (August 1st-August 28th). (TIFF) [file pone.0287332.s004.tiff]

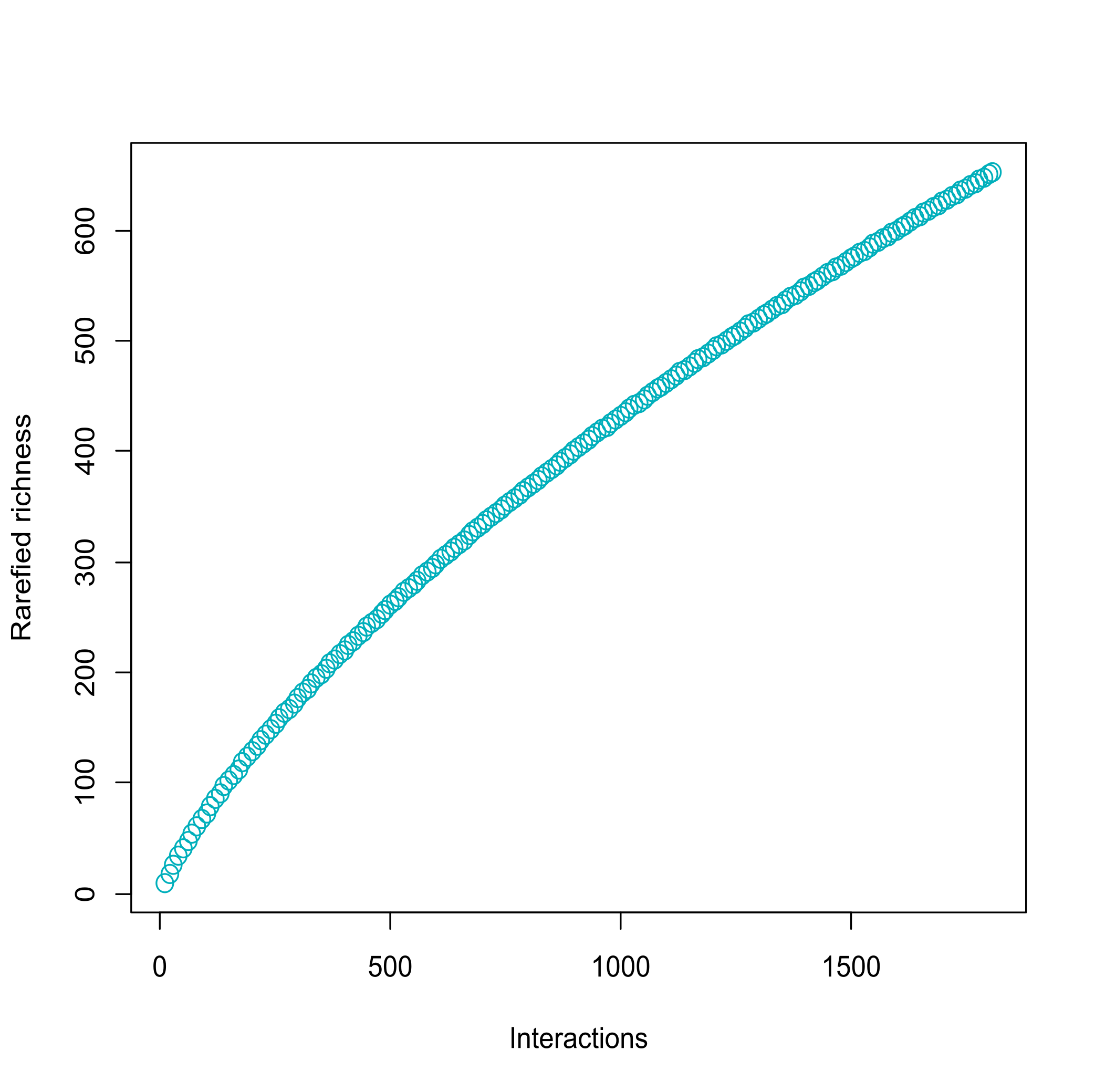

Supplement: S4 Fig — This figure shows, for a given number of re-sampled interactions from our full season all taxa dataset (x-axis), the mean number of unique interactions detected (y-axis). There was a total observed interaction richness of 654, and Chao1 estimated 1,779 interactions (95% confidence interval: 1500–2148 interactions), meaning 31–44% of the estimated interactions were observed. (TIFF) [file pone.0287332.s005.tiff]
