# Supplementary material for: Honey bees (Apis mellifera) modify plant-pollinator network structure, but do not alter wild species’ interactions
Source: PLoS One. 2023 Jul 13;18(7):e0287332. doi: 10.1371/journal.pone.0287332 (PMC10343163; doi:10.1371/journal.pone.0287332)
Supplement: S1 Table — The northernmost, central, and southernmost hive locations are listed as Bee48, Bee32, and Bee16 respectively, indicating their number of hives. Each transect is indicated by its treatment (100 m, 500 m, or 5000 m distance from a hive location). Letters indicate each replicated set of distances from hives (See Fig 1). G5000 indicates the new location sampled at once sampling at F5000 was discontinued mid-season. (DOCX) [file pone.0287332.s006.docx]

Table S1. Longitude and latitude for each hive location and sampling transect, and collection effort at each transect. The northernmost, central, and southernmost hive locations are listed as Bee48, Bee32, and Bee16 respectively, indicating their number of hives. Each transect is indicated by its treatment (100 m, 500 m, or 5000 m distance from a hive location). Letters indicate each replicated set of distances from hives (See Fig 1). G5000 indicates the new location sampled at once sampling at F5000 was discontinued mid-season.

| **Longitude** | **Latitude** | **Transect** | **Number of Collections** |
| --- | --- | --- | --- |
| -111.91850 | 50.90210 | Bee48 |  |
| -111.94658 | 50.88120 | Bee32 |  |
| -111.93580 | 50.84320 | Bee16 |  |
| -111.91992 | 50.90202 | A100 | 10 |
| -111.92545 | 50.90248 | A500 | 10 |
| -111.98687 | 50.91594 | A5000 | 8 |
| -111.91699 | 50.90205 | B100 | 7 |
| -111.91010 | 50.90265 | B500 | 6 |
| -111.85447 | 50.88185 | B5000 | 8 |
| -111.94792 | 50.88178 | C100 | 9 |
| -111.95325 | 50.88365 | C500 | 8 |
| -112.00554 | 50.90691 | C5000 | 8 |
| -111.94533 | 50.88081 | D100 | 9 |
| -111.94149 | 50.87831 | D500 | 9 |
| -111.87483 | 50.86788 | D5000 | 8 |
| -111.93735 | 50.84338 | E100 | 3 |
| -111.94283 | 50.84407 | E500 | 8 |
| -111.99767 | 50.93795 | E5000 | 7 |
| -111.93415 | 50.84310 | F100 | 8 |
| -111.92928 | 50.84510 | F500 | 7 |
| -111.86687 | 50.83544 | F5000 | 2 |
| -111.98858 | 50.95827 | G5000 | 5 |
