# Supplementary material for: Honey bees (Apis mellifera) modify plant-pollinator network structure, but do not alter wild species’ interactions
Source: PLoS One. 2023 Jul 13;18(7):e0287332. doi: 10.1371/journal.pone.0287332 (PMC10343163; doi:10.1371/journal.pone.0287332)
Supplement: S2 Table — Full citations are listed in the References section of the Supporting Information. (DOCX) [file pone.0287332.s007.docx]

Table S2. List of references and resources used in species identifications. Full citations are listed in the References section of the Supporting Information.

| **Insect Group** | **References** | **Museum Resources** | **Expert Taxonomists** |
| --- | --- | --- | --- |
| Butterflies  (Lepidoptera - Papilionoidea) | - Acorn (1993) - [1] - Vandyk (2021) - [2] - Warren et al (2016) - [3] | - University of Alberta Strickland Museum | John Acorn |
| Moths (Lepidoptera) |  | - Northern Forestry Centre | Greg Pohl |
| Ants (Hymenoptera – Formicoidea) | - Glasier et al (2013) - [4] |  | James Glasier |
| Beetles (Coleoptera) | - Acorn (2001) - [5] - Acorn (2007) - [6] - Arnett and Thomas (2000) - [7] - Arnett et al (2002) - [8] - Bousquet et al (2013) - [9] - Lindroth (1961) - [10] - Pinto (1991) - [11] - Smith and Skelley (2020) - [12] - Vandyk (2021) - [2] | - University of Alberta Strickland Museum | John Acorn |
| Flies (Diptera) | - Aldrich (1926) - [13] - Aldrich (1928) - [14] - Ávalos-Hernández (2009) - [15] - Brooks (1943a) - [16] - Brooks (1943b) - [17] - Brooks (1945) - [18] - Burt (2015) - [19] - Camras (1944) - [20] - Camras (1945) - [21] - Camras (1957) - [22] - Cole (1923) - [23] - Coquillett (1897) - [24] - Curran (1921) - [25] - Curran (1923) - [26] - Curran (1935) - [27] - Curran (1939) - [28] - Curran (1941) - [29] - Curran and Fluke (1926) - [30] - Fluke and Weems (1956) - [31] - Foster and Mathis (2012) - [32] - Gibson (2017) - [33] - Gill (1962) - [34] - Hardy (1943) - [35] - Huckett (1954) - [36] - James (1936) - [37] - James (1974) - [38] - James and Steyskal (1952) - [39] - Kits et al. (2008) - [40] - Malloch (1918) - [41] - Malloch (1920) - [42] - Malloch (1921) - [43] - Martin (1959) - [44] - McAlpine (1981) - [45] - McAlpine (1993) - [46] - Miranda et al (2013) - [47] - O’Hara (1982) - [48] - O’Hara and Wood (2004) - [49] - Sabrosky (1935) - [50] - Sabrosky (1955) - [51] - Sabrosky (1967) - [52] - Schlinger (1960) - [53] - Sedman (1966) - [54] - Shannon (1926) - [55] - Shannon (1939) - [56] - Skevington and Thompson (2012) - [57] - Strickland (1938) - [58] - Sun and Marshall (2003) - [59] - Telford (1970) - [60] - Thompson (1981) - [61] - Thompson et al (1990) - [62] - Townsend (1891) - [63] - Townsend (1908) - [64] - Vockeroth (1992) - [65] - Webb et al (2013) - [66] - Whitworth (2006) - [67] - Wilder (1979) - [68] - Young et al (2016) - [69] | - University of Alberta Strickland Museum | Brittany Wingert |
| Bees (Hymenoptera - Anthophila) | - Cockerell (1896) - [70] - Cockerell (1897) - [71] - Cockerell (1902) - [72] - Cockerell (1936) - [73] - Cockerell (1937a) - [74] - Cockerell (1937b) - [75] - Cockerell (1937c) - [76] - Cresson (1869) - [77] - Cresson (1878) - [78] - Droege et al (2010) - [79] - Droege et al (2021) - [80] - Dumesh and Sheffield (2012) - [81] - Dumesh and Sheffield (2014) - [82] - Gibbs (2010) - [83] - Hurd and Michener (1955) - [84] - LaBerge (1956) - [85] - LaBerge (1961) - [86] - McGinley (1986) - [87] - Michener (2007) - [88] - Mitchell (1956) - [89] - Mitchell (1960) - [90] - Mitchell (1962) - [91] - Mitchell (1973) - [92] - Onuferko (2017) - [93] - Packer et al (2007) - [94] - Rightmyer (2008) - [95] - Roberts (1973a) - [96] - Roberts (1973b) - [97] - Sheffield et al. (2011) - [98] - Sheffield et al. (2014) - (99] - Stephen (1954) - [100] - Viereck and Cockerell (1914) - [101] - Williams et al (2014) - [102] - York University (n.d.) - [103] | - University of Alberta Strickland Museum - University of Calgary Museum of Zoology | Lincoln Best |
| Wasps (Hymenoptera - Apocrita) | - Goulet and Huber (1993) - [104] - Kimsey and Carpenter (2012) - [105] | Royal Alberta Museum | Matthias Buck |
| Plants | - Bain et al (2014) - [106] - Moss (1994) - [107] - Tannas (2003) - [108] - Tannas (2004) - [109] |  |  |

## References

1. Acorn J. Butterflies of Alberta. Edmonton: Lone Pine Publishing; 1993.

2. Vandyk J, editor. BugGuide.Net: Identification, Images, & Information For Insects, Spiders & Their Kin For the United States & Canada. [Internet]. Iowa State University; 2021 [cited 2021 Nov 29]. Available from: https://bugguide.net/

3.Warren AD, Davis KJ, Strangeland EM, Pelham JP, Willmott KR, Grishin NV. Butterflies of America [Internet]. Illustrated Lists of American Butterflies (North and South Amerca). 2016 [cited 2021 Nov 29]. Available from: https://www.butterfliesofamerica.com/

4.Glasier JRN, Acorn, John H., Nielsen SE, Proctor H. Ants (Hymenoptera: Formicidae) of Alberta: A key to species based primarily on the worker caste. CJAI. 2013 Jul 4;22.

5. Acorn J. The Tiger Beetles of Alberta: Killers on the Clay, Stalkers on the Sand. Edmonton: University of Alberta Press; 2001.

6. Acorn J. Ladybugs of Alberta: finding the spots and connecting the dots. 1st ed., 1st print., 2007. Edmonton: University of Alberta Press; 2007. 169 p. (Alberta insects series).

7. Arnett RH, Thomas MC, editors. American beetles, Volume I: Archostemata, Myxophaga, Adephaga, Polyphaga: Staphyliniformia. Boca Raton, Fla: CRC Press; 2000. 2 p.

8. Arnett RH, Thomas MC, Skelley PE, Frank JH, editors. American beetles, Volume II: Polyphaga: Scarabaeoidea through Curculionoidea. Boca Raton, Fla: CRC Press; 2002. 2 p.

9. Bousquet Y, Bouchard P, Davies A, Sikes D. Checklist of beetles (Coleoptera) of Canada and Alaska. Second edition. ZK. 2013 Dec 6;360:1–44.

10. Lindroth CH. The Ground-Beetles (Carabidae, Excluding Cicindelinae) of Canada and Alaska. Opuscula Entomologica [Internet]. 1961 [cited 2021 Nov 29];Supplementa XX, XXIV, XXIX, XXXIII, XXXIV, XXXV. Available from: https://academic.oup.com/sysbio/article-lookup/doi/10.2307/2412348

11. Pinto JD. The taxonomy of North American Epicauta (Coleoptera: Meloidae), with a revision of the nominate subgenus and a survey of courtship behavior. Berkeley: University of California Press; 1991. 372 p. (University of California publications in entomology).

12. Smith ABT, Skelley PE. A New Species of Flaviellus Gordon and Skelley, 2007 (Coleoptera: Scarabaeidae: Aphodiinae) from the Yukon, Canada. The Coleopterists Bulletin. 2020 Mar 25;74(1):101.

13. Aldrich JM. North American two-winged flies of the genus Cylindromyia Meigen (Ocyptera of authors). Proceedings of the United States National Museum. 1926;68(2624):1–27.

14. Aldrich JM. A revision of the American parasitic flies belonging to the genus Belvosia. Proceedings of the United States National Museum. 1928;73(2729):1–45.

15. Ávalos-Hernández O. A Review Of The North American Species Of Hemipenthes Loew, 1869 (Diptera: Bombyliidae). 2009 Dec 31 [cited 2022 Jan 28]; Available from: https://zenodo.org/record/187152

16. Brooks AR. A REVIEW OF THE CANADIAN SPECIES OF ERNESTIA SENS. LAT. (TACHINIDAE, DIPTERA). Can Entomol. 1943 Apr;75(4):66–78.

17. Brooks AR. A REVIEW OF THE NORTH AMERICAN SPECIES OF GONIA sens. lat. (DIPTERA, TACHINIDAE). Can Entomol. 1943 Dec;75(12):219–36.

18. Brooks AR. A REVISION OF THE NORTH AMERICAN SPECIES OF THE RHODOGYNE COMPLEX (DIPTERA, LARVAEVORIDAE). Can Entomol. 1945 Dec;77(12):218–30.

19. Burt T. Taxonomic revision of four Nearctic Conopidae (Insecta: Diptera) genera (Dalmannia, Roberstonomyia, Stylogaster and Zodion) with notes on all other Nearctic genera [Internet]. [Ottawa, Ontario]: Carleton University; 2015 [cited 2021 Oct 6]. Available from: https://curve.carleton.ca/system/files/etd/83a38375-1d49-4c32-8c2c-2b0f0b9301a1/etd_pdf/1c9eb60a06629211d3fe3d6e7f5ffdff/burt-taxonomicrevisionoffournearcticconopidaeinsecta.pdf

20. Camras S. Notes on the North American species of the Zodion fulvifrons group (Diptera: Conopidae). The Pan-Pacific Entomologist. 1944;20(4):121–8.

21. Camras S. A Study of the Genus Occemyia in North America (Diptera: Conopidae)1. Annals of the Entomological Society of America. 1945 Jun 1;38(2):216–22.

22. Camras S. A Review of the New World Physocephala (Diptera: Conopidae). Annals of the Entomological Society of America. 1957 May 1;50(3):213–8.

23. Cole FR. A revision of the North American two-winged flies of the family Therevidae. Proceedings of the United States National Museum. 1923;62(2450):1–140.

24. Coquillett DW. Revision of the Tachinidae of America north of Mexico: a family of parasitic two-winged insects [Internet]. Washington: Government Printing Office; 1897 [cited 2022 Jan 27]. Available from: http://www.biodiversitylibrary.org/bibliography/87236

25. Curran CH. Revision of the Pipiza group of the family Syrphidae (flower-flies) from north of Mexico. Proceedings of the California Academy of Sciences. 1921;11(16):345–93.

26. Curran CH. Notes on the Genus Pipizella Rondani, with Descriptions of New Species (Diptera; Syrphidae). Transactions of the American Entomological Society. 1923;49(4):339–45.

27. Curran CH. The families and genera of North American Diptera. American Museum Novitates [Internet]. 1935 [cited 2022 Jan 28];812. Available from: http://www.biodiversitylibrary.org/bibliography/6825

28. Curran CH. The species of Temnostoma related to bombylans Linné (Syrphidae, Diptera). American Museum Novitates. 1939;1040.

29. Curran CH. New American Syrphidae. Bulletin of the American Museum of Natural History. 1941;78(3):243–304.

30. Curran CH, Fluke CL. Revision of the nearctic species of Helophilus and allied genera. Transactions of the Wisconsin Academy of Sciences, Arts and Letters. 1926;207–81.

31. Fluke CL, Weems JrHV. The Myoleptini of the Americas (Diptera, Syrphidae). American Museum Novitates. 1956;1758:1–23.

32. Foster GA, Mathis WN. A revision of the nearctic species of the genus Trixoscelis Rondani (Diptera: Heleomyzidae: Trixoscelidinae). Smithsonian Contributions to Zoology. 2012;(637):1–128.

33. Gibson JF. An updated and annotated checklist of the thick-headed flies (Diptera: Conopidae) of British Columbia, the Yukon, and Alaska. Journal of the Entomological Society of British Columbia. 2017;114.

34. Gill GD. The Heleomyzid Flies of American North of Mexico (Diptera: Heleomyzidae). Proceedings of the United States National Museum. 1962;113(3465):495–603.

35. Hardy E. New Nearctic Pipunculidae (Diptera). Journal of the Kansas Entomological Society. 1943;12(1):16–25.

36. Huckett HC. A Review of the North American Species Belonging to the Genus Hydrotaea Robineau-Desvoidy (Diptera, Muscidae). Annals of the Entomological Society of America. 1954 Jun 1;47(2):316–42.

37. James MT. The Genus Odontomyia in America North of Mexico (Diptera, Stratiomyidae). Annals of the Entomological Society of America. 1936 Sep 1;29(3):517–50.

38. James MT. The Status of Odontomyia arcuata Loew, O. inaequalis Loew, and Their Close Relatives in Western North America (Diptera: Stratiomyidae). Journal of the Kansas Entomological Society. 1974;47(2):222–6.

39. James MT, Steyskal GC. A Review of the Nearctic Stratiomyini (Diptera, Stratiomyidae). Annals of the Entomological Society of America. 1952 Sep 1;45(3):385–412.

40. Kits JH, Marshall SA, Evenhuis NL. The Bee Flies (Diptera: Bombyliidae) of Ontario, with a Key to the Species of Eastern Canada. CJAI. 2008;6.

41. Malloch JR. Diptera from the South-Western United States. Paper IV. Anthomyiidae. Transactions of the American Entomological Society. 1918;44(3):263–319.

42. Malloch JR. A SYNOPTIC REVISION OF THE ANTHOMYIID GENUS HYDROPHORIA ROBINEAU-DESVOIDY (DIPTERA). Can Entomol. 1920 Nov;52(11):253–7.

43. Malloch JR. A SYNOPSIS OF THE NORTH AMERICAN SPECIES OF THE GENUS HELINA R.-D., SENS. LAT. (DIPTERA, ANTHOMYIIDAE). Can Entomol. 1921 May;53(5):103–9.

44. Martin CH. The Holopogon complex of North America, excluding Mexico, with the descriptions of a new genus and a new subgenus (Diptera, Asilidae). American Museum Novitates. 1959;1980.

45. McAlpine JF, editor. Manual of Nearctic Diptera. Vol. 1. Ottawa: Research Branch, Agriculture Canada; 1981. (Monograph / Research Branch, Agriculture Canada; vol. 1).

46. McAlpine JF, editor. Manual of Nearctic Diptera. Vol. 2. Repr. Hull, Que: Canadian Government Publ. Centre; 1993. 675 p. (Monograph / Research Branch, Agriculture Canada; vol. 2).

47. Miranda GFG, Young AD, Locke MM, Marshall SA, Skevington JH, Thompson FC. Key to the Genera of Nearctic Syrphidae. CJAI. 2013;23:1–351.

48. O’Hara JE. Classification, phylogeny and zoogeography of the North American species of Siphona Meigen (Diptera: Tachinidae). Department of Entomology, University of Alberta. 1982;18:261–380.

49. O’Hara JE, Wood DM. Checklist of the Tachinidae (Diptera) of America north of Mexico. Online [Internet]. 2004 [cited 2021 Jan 28]; Available from: http://www.nadsdiptera.org/Tach/WorldTachs/TTimes/Tach18.html

50. Sabrosky CW. The Chloropidae of Kansas (Diptera). Transactions of the American Entomological Society. 1935;61(3):207–68.

51. Sabrosky CW. A Third Species of Eusiphona, with Remarks on the Systematic Position of the Genus (Diptera, Milichiidae). Entomological News. 1955;66(7):169–73.

52. Sabrosky CW. Notes on the tachinid genus Cylindromyia in North America. Proceedings of the Entomological Society of Washington. 1967;69(1):60–3.

53. Schlinger EI. A Revision of the Genus Ogcodes Latreille with Particular Reference to Species of the Western Hemisphere. Proceedings of the United States National Museum. 1960;111(3429):227–336.

54. Sedman YS. The Chrysogaster (Orthonevra) pictipennis group in North America (Diptera: Syrphidae). Entomological Society of Washington. 1966;68:185–94.

55. Shannon RC. Review of the American xylotine syrphid-flies. Proceedings of the United States National Museum. 1926;69(2635):1–52.

56. Shannon RC. Temnostoma bombylans and related species (Syrphidae, Diptera). Entomological Society of Washington. 1939;41(7):215–24.

57. Skevington JH, Thompson FC. Review of New World Sericomyia (Diptera: Syrphidae), including description of a new species. Can Entomol. 2012 Apr;144(2):216–47.

58. Strickland EH. AN ANNOTATED LIST OF THE DIPTERA (FLIES) OF ALBERTA. Can J Res. 1938 Jul 1;16d(7):175–219.

59. Sun X, Marshall SA. Systematics of Phasia Latreille (Diptera: Tachinidae). Zootaxa. 2003 Aug 29;276(1):1.

60. Telford HS. Eristalis (Diptera: Syrphidae) from America North of Mexico1. Annals of the Entomological Society of America. 1970 Sep 15;63(5):1201–10.

61. Thompson FC. Revisionary notes on Nearctic Microdon flies (Diptera: Syrphidae). Proceedings of the Entomological Society of Washington. 1981;83:725–58.

62. Thompson FC, Fee FD, Bezark LD. Two Immigrant Synanthropic Flower Flies (Diptera: Syrphidae) New to North America. Entomological News. 1990;101(2):69–74.

63. Townsend CHT. NOTES ON NORTH AMERICAN TACHINIDÆ, WITH DESCRIPTIONS OF NEW GENERA AND SPECIES. Paper II. Can Entomol. 1891;18(4):349–82.

64. Townsend CHT. The taxonomy of the muscoidean flies, including descriptions of new genera and species. Smithsonian Miscellaneous Collections. 1908;51(2):1–138.

65. Vockeroth JR. The Flower Flies of the Subfamily Syrphinae of Canada, Alaska, and Greenland: Diptera: Syrphidae. Ottawa, Ont: Centre for Land and Biological Resources Research, Research Branch, Agriculture Canada; 1992. 456 p. (The Insects and Arachnids of Canada).

66. Webb DW, Gaimari SD, Hauser M, Holston KC, Metz MA, Irwin ME, et al. An annotated catalogue of the New World Therevidae (Insecta: Diptera: Asiloidea). Zootaxa. 2013 Jan 11;3600(1):1–105.

67. Whitworth T. Keys to the Genera and Species of Blow Flies (Diptera: Calliphoridae) of America, North of Mexico. Proceedings of the Entomological Society of Washington. 2006;108(3):689–725.

68. Wilder DD. Systematics of the Nearctic Ptilodexia Brauer and Bergenstamm (Diptera, Tachinidae). Proceedings of the California Academy of Sciences. 1979;42(1):55.

69. Young AD, Marshall SA, Skevington JH. Revision of Platycheirus Lepeletier and Serville (Diptera: Syrphidae) in the Nearctic north of Mexico. Zootaxa. 2016 Feb 17;4082(1):1.

70. Cockerell TDA. The Bees of the Genus Perdita F. Smith. Proceedings of the Academy of Natural Sciences of Philadelphia. 1896;48:25–107.

71. Cockerell TDA. New species of Andrena from North America. The Entomologist. 1897;30:305–9.

72. Cockerell TDA. North American bees of the Genus Andrena. Annals and Magazine of Natural History. 1902 Feb;9(50):101–6.

73. Cockerell TDA. THE BEES OF ALBERTA.—I. Can Entomol. 1936 Dec;68(12):274–7.

74. Cockerell TDA. Bees of the Genus Sphecodes from Saskatchewan. Vol. 909. The American Museum of Natural History; 1937.

75. Cockerell TDA. THE BEES OF ALBERTA—IV. Can Entomol. 1937 May;69(5):113–4.

76. Cockerell TDA. BEES OF ALBERTA. V. Can Entomol. 1937 Jun;69(6):126–7.

77. Cresson ET. A list of the North American species of the genus Anthophora, with descriptions of new species. In Transactions of the American Entomological Society; 1869. p. 289–93.

78. Cresson ET. Descriptions of new species of North American bees. In Proceedings of the Academy of Natural Sciences of Philadelphia; 1878. p. 181–221.

79. Droege S, Rightmyer MG, Sheffield CS, Brady SG. New synonymies in the bee genus Nomada from North America (Hymenoptera: Apidae). Zootaxa. 2010 Oct 29;2661(1):1.

80. Droege S, Jean R, Orr M. Bee Genera of Eastern North America [Internet]. Apoidea - Discover Life. 2021 [cited 2020 Aug 2]. Available from: https://www.discoverlife.org/20/q?search=Apoidea

81. Dumesh S, Sheffield CS. Bees of the Genus Dufourea Lepeletier (Hymenoptera: Halictidae: Rophitinae) of Canada. CJAI. 2012 May;18.

82. Dumesh S, Sheffield CS. Photographic keys to the bees of the Northwest Territories, Canada. Department of Environment and Natural Resources, Government of the Northwest Territories.; 2014. 257 p.

83. Gibbs J. Revision of the metallic species of Lasioglossum (Dialictus) in Canada (Hymenoptera, Halictidae, Halictini). Zootaxa. 2010 Aug 31;2591(1):1.

84. Hurd PD, Michener CD. The Megachiline bees of California (Hymenoptera : Megachilidae). University of California Press Berkeley and Los Angeles; 1955. 251 p. (Bulletin of the California Insect Survey; vol. 3).

85. LaBerge WE. A Revision of the Bees of the Genus Melissodes in North and Central America. Parts I., II. (Hymenoptera, Apidae). The University of Kansas science bulletin. 1956;37:911–1194.

86. LaBerge WE. A Revision of the Bees of the Genus Melissodes in North and Central America. Part III (Hymenoptera, Apidae). The University of Kansas science bulletin. 1961;42:283–663.

87. McGinley RJ. Studies of Halictinae (Apoidea: Halictidae), I: Revision of New World Lasioglossum Curtis. Smithsonian Contributions to Zoology. 1986;(429):1–294.

88. Michener CD. The Bees of the World. 2nd ed. Baltimore: Johns Hopkins University Press; 2007. 953 p.

89. Mitchell TB. New Species of Sphecodes from the Eastern United States. Journal of the Elisha Mitchell Scientific Society. 1956;72(2):206–22.

90. Mitchell TB. Bees of the Eastern United States, V1. Raleigh: North Carolina Agricultural Experiment Station Technical Bulletin; 1960. 538 p.

91. Mitchell TB. Bees of the Eastern United States, V2. Raleigh: North Carolina Agricultural Experiment Station Technical Bulletin; 1962. 557 p.

92. Mitchell TB. A subgeneric revision of the bees of the genus Coelioxys of the Western Hemisphere (Hymenoptera: Megachilidae). Department of Entomology, North Carolina State University; 1973. 129 p.

93. Onuferko TM. Cleptoparasitic bees of the genus Epeolus Latreille (Hymenoptera: Apidae) in Canada. CJAI. 2017;30:1–62.

94. Packer L, Genaro JA, Sheffield CS. The Bee Genera of Eastern Canada. CJAI. 2007;3:1–32.

95. Rightmyer MG. A review of the cleptoparasitic bee genus Triepeolus (Hymenoptera: Apidae).-Part I. Zootaxa. 2008 Feb 22;1710(1):1.

96. Roberts RB. Bees of northwestern America: Agapostemon (Hymenoptera: Halictidae). Technical Bulletin of the Agricultural Experiment Station. 1973;125:1–23.

97. Roberts RB. Bees of northwestern America: Halictus (Hymenoptera: Halictidae). Technical Bulletin of the Agricultural Experiment Station [Internet]. 1973 [cited 2021 Nov 29];126. Available from: https://static1.squarespace.com/static/5a849d4c8dd041c9c07a8e4c/t/5a9727410d9297d03d5f566c/1519855427669/Roberts+1973+Bees+of+Northwestern+America+Halictus.pdf

98. Sheffield CS, Ratti C, Packer L, Griswold T. Leafcutter and Mason Bees of the Genus Megachile Latreille (Hymenoptera: Megachilidae) in Canada and Alaska. CJAI. 2011 Nov 29;18.

99. Sheffield CS, Frier SD, Dumesh, S. The Bees (Hymenoptera: Apoidea, Apiformes) of the Prairies Ecozone with Comparisons to other Grasslands of Canada. 2014;427–67.

100. Stephen WP. A Revision of the Bee Genus Colletes in America North of Mexico (Hymenoptera Colletidae). The University of Kansas Science Bulletin. 1954;36(6):149–527.

101. Viereck HL, Cockerell TDA. New North American bees of the genus Andrena. Proceedings of the United States National Museum. 1914;48(2064):1–58.

102. Williams P, Thorp RW, Richardson L, Colla S. Bumble bees of North America: an identification guide. Princeton: Princeton University Press; 2014. 208 p.

103.York University. Key to the Genera of Andreninae [Internet]. York University; [cited 2020 Jul 19]. Available from: https://www.yorku.ca/bugsrus/resources/keys/Andreninae/Images/Andreninae_A_Start/Andreninae_A_Start_Export.htm

104. Goulet H, Huber JT, editors. Hymenoptera of the world: an identification guide to families. Ottawa, Ontario: Centre for Land and Biological Resources Research; 1993. 668 p. (Publication).

105. Kimsey L, Carpenter J. The Vespinae of North America (Vespidae, Hymenoptera). JHR. 2012 Aug 24;28:37–65.

106. Bain J, Flanagan J, Kuigt J. Common Coulee Plants of Southern Alberta. 2nd ed. University of Lethbridge Herbarium.; 2014.

107. Moss EH, Packer JG. Flora of Alberta [Internet]. 2nd ed. University of Toronto Press; 1994 [cited 2020 Aug 2]. Available from: http://www.jstor.org/stable/10.3138/j.ctt1287xvf

108. Tannas K. Common plants of the western rangelands, volume 2: Trees and Shrubs. Olds College; 2003.

109. Tannas K. Common plants of the western rangelands, volume 3: Forbs. Olds College; 2004.
