# Supplementary material for: Honey bees (Apis mellifera) modify plant-pollinator network structure, but do not alter wild species’ interactions
Source: PLoS One. 2023 Jul 13;18(7):e0287332. doi: 10.1371/journal.pone.0287332 (PMC10343163; doi:10.1371/journal.pone.0287332)
Supplement: S3 Table — Each row and column header refers to the following metrics, respectively: G, generality; V, vulnerability; Poll. NO, pollinator niche overlap; Pl. NO, plant niche overlap; Poll. FC, pollinator functional complementarity; Pl. FC, plant functional complementarity; WNODF, weighted nestedness based on overlap and decreasing fill; M, modularity; WC, weighted connectance; LD, link density; ISA, interaction strength asymmetry; IE, interaction evenness. (DOCX) [file pone.0287332.s008.docx]

Table S3. Pearson correlations between the response variables (network metrics). Each row and column header refers to the following metrics, respectively: G, generality; V, vulnerability; Poll. NO, pollinator niche overlap; Pl. NO, plant niche overlap; Poll. FC, pollinator functional complementarity; Pl. FC, plant functional complementarity; WNODF, weighted nestedness based on overlap and decreasing fill; M, modularity; WC, weighted connectance; LD, link density; ISA, interaction strength asymmetry; IE, interaction evenness.

|  | **G** | **V** | **Poll. NO** | **Pl. NO** | **Poll. FC** | **Pl. FC** | **WNODF** | **M** | **WC** | **LD** | **ISA** | **IE** |
| --- | --- | --- | --- | --- | --- | --- | --- | --- | --- | --- | --- | --- |
| **G** |  | 0.2697 | -0.6359 | 0.6946 | 0.2326 | 0.1871 | 0.6796 | 0.4069 | -0.5348 | 0.3489 | -0.6631 | 0.1584 |
| **V** | 0.2697 |  | 0.3847 | 0.1610 | 0.0598 | -0.0021 | 0.1004 | 0.1328 | 0.1491 | 0.9965 | 0.2703 | 0.4320 |
| **Poll. NO** | -0.6359 | 0.3847 |  | -0.2876 | -0.2328 | -0.2307 | -0.2577 | -0.5865 | 0.7461 | 0.3195 | 0.7857 | 0.1155 |
| **Pl. NO** | 0.6946 | 0.1610 | -0.2876 |  | 0.1473 | 0.1077 | 0.7357 | 0.0417 | -0.2985 | 0.2167 | -0.3601 | 0.0293 |
| **Poll. FC** | 0.2326 | 0.0598 | -0.2328 | 0.1473 |  | 0.9922 | 0.0476 | 0.0006 | -0.7518 | 0.0783 | -0.2365 | -0.7252 |
| **Pl. FC** | 0.1871 | -0.0021 | -0.2307 | 0.1077 | 0.9922 |  | 0.0147 | -0.0267 | -0.7322 | 0.0141 | -0.2439 | -0.7601 |
| **WNODF** | 0.6796 | 0.1004 | -0.2577 | 0.7357 | 0.0476 | 0.0147 |  | -0.0233 | -0.2743 | 0.1564 | -0.5198 | 0.0206 |
| **M** | 0.4069 | 0.1328 | -0.5865 | 0.0417 | 0.0006 | -0.0267 | -0.0233 |  | -0.4000 | 0.1644 | -0.3664 | 0.4278 |
| **WC** | -0.5348 | 0.1491 | 0.7461 | -0.2985 | -0.7518 | -0.7322 | -0.2743 | -0.4000 |  | 0.0989 | 0.6602 | 0.5171 |
| **LD** | 0.3489 | 0.9965 | 0.3195 | 0.2167 | 0.0783 | 0.0141 | 0.1564 | 0.1644 | 0.0989 |  | 0.2058 | 0.4341 |
| **ISA** | -0.6631 | 0.2703 | 0.7857 | -0.3601 | -0.2365 | -0.2439 | -0.5198 | -0.3664 | 0.6602 | 0.2058 |  | 0.1623 |
| **IE** | 0.1584 | 0.4320 | 0.1155 | 0.0293 | -0.7252 | -0.7601 | 0.0206 | 0.4278 | 0.5171 | 0.4341 | 0.1623 |  |
