# Supplementary material for: Honey bees (Apis mellifera) modify plant-pollinator network structure, but do not alter wild species’ interactions
Source: PLoS One. 2023 Jul 13;18(7):e0287332. doi: 10.1371/journal.pone.0287332 (PMC10343163; doi:10.1371/journal.pone.0287332)
Supplement: S4 Table — “Collections” refers to the number of collection rounds at each transect, “Honey bee abundance” refers to honey bee abundance at each transect, “Flower species” refers to the number of flowering species at each transect, and “Flower abundance” refers to the number of individual flowers at each transect. (DOCX) [file pone.0287332.s009.docx]

Table S4. Pearson correlations between predictor variables. “Collections” refers to the number of collection rounds at each transect, “Honey bee abundance” refers to honey bee abundance at each transect, “Flower species” refers to the number of flowering species at each transect, and “Flower abundance” refers to the number of individual flowers at each transect.

|  | Honey bee abundance | Collections | Flower species | Flower abundance |
| --- | --- | --- | --- | --- |
| Honey bee abundance |  | 0.4454 | 0.6178 | 0.8517 |
| Collections | 0.4454 |  | 0.4343 | 0.3848 |
| Flower species | 0.6178 | 0.4343 |  | 0.5981 |
| Flower abundance | 0.8517 | 0.3848 | 0.5981 |  |
