# Supplementary material for: Honey bees (Apis mellifera) modify plant-pollinator network structure, but do not alter wild species’ interactions
Source: PLoS One. 2023 Jul 13;18(7):e0287332. doi: 10.1371/journal.pone.0287332 (PMC10343163; doi:10.1371/journal.pone.0287332)
Supplement: S5 Table — The “Lower 95%” column indicates the observed interactions divided by the lowest Chao1 estimated total number of true interactions, and the “Upper 95%” column indicates the observed interactions divided by the highest Chao1 estimated total number of true interactions (which is why the “Lower” values are higher). Shaded cells denote networks that had too few species for some network metrics to be meaningful, so these replicates were excluded from analysis for the metrics: generality, vulnerability, plant and pollinator niche overlap, and nestedness. (DOCX) [file pone.0287332.s010.docx]

Table S5. Percent sampling completeness and 95% confidence intervals, calculated as the number of unique interactions observed, divided by the Chao1 estimated total number of unique interactions, and multiplied by 100, for the full season all taxa, mid-season all taxa, and full season bees-only datasets. The “Lower 95%” column indicates the observed interactions divided by the lowest Chao1 estimated total number of true interactions, and the “Upper 95%” column indicates the observed interactions divided by the highest Chao1 estimated total number of true interactions (which is why the “Lower” values are higher). Shaded cells denote networks that had too few species for some network metrics to be meaningful, so these replicates were excluded from analysis for the metrics: generality, vulnerability, plant and pollinator niche overlap, and nestedness.

| Transect | Full Season Chao (%) | Full Season (Lower 95%) | Full Season (Upper 95%) | Mid-Season Chao (%) | Mid-Season (Lower 95%) | Mid-Season (Upper 95%) | Full Season Bees Only Chao (%) | Full Season Bees Only (Lower 95%) | Full Season Bees Only (Upper 95%) |
| --- | --- | --- | --- | --- | --- | --- | --- | --- | --- |
| A100 | 32.261 | 56.043 | 15.104 | 42.155 | 74.898 | 15.109 | 51.891 | 85.115 | 16.904 |
| A500 | 37.002 | 60.298 | 18.510 | 21.937 | 54.374 | 6.215 | 41.172 | 73.312 | 15.133 |
| A5000 | 30.264 | 55.443 | 13.146 | 31.239 | 62.984 | 10.818 | 58.120 | 85.569 | 24.515 |
| B100 | 19.273 | 49.517 | 5.492 | 42.996 | 85.010 | 9.120 | 26.521 | 70.481 | 5.174 |
| B500 | 37.534 | 66.456 | 15.415 | NA | NA | NA | 73.275 | 94.453 | 94.453 |
| B5000 | 25.159 | 46.371 | 11.559 | 43.914 | 77.220 | 15.313 | 23.161 | 56.628 | 6.506 |
| C100 | 46.666 | 66.435 | 27.892 | 25.828 | 60.420 | 7.358 | 71.861 | 90.536 | 40.544 |
| C500 | 33.590 | 50.239 | 20.217 | 51.112 | 74.850 | 26.860 | 26.942 | 57.414 | 9.164 |
| C5000 | 50.330 | 68.571 | 32.000 | 50.051 | 74.673 | 25.405 | 53.648 | 84.379 | 19.870 |
| D100 | 21.306 | 42.350 | 9.073 | 21.265 | 47.044 | 7.588 | 33.229 | 65.748 | 11.428 |
| D500 | 24.903 | 45.944 | 11.456 | 41.776 | 78.793 | 12.170 | 12.111 | 44.338 | 2.328 |
| D5000 | 20.522 | 46.661 | 7.081 | 31.528 | 68.871 | 8.745 | 28.679 | 64.485 | 8.177 |
| E100 | 44.262 | 80.830 | 13.009 | NA | NA | NA | NA | NA | NA |
| E500 | 31.594 | 48.799 | 18.289 | 35.263 | 58.631 | 17.311 | 37.100 | 62.525 | 17.254 |
| E5000 | 36.864 | 63.866 | 16.170 | 15.967 | 53.154 | 3.084 | 62.461 | 91.466 | 20.538 |
| F100 | 26.546 | 52.593 | 10.533 | 46.701 | 76.693 | 18.917 | 42.938 | 79.319 | 12.865 |
| F500 | 40.013 | 72.013 | 14.742 | 50.505 | 85.046 | 15.473 | 25.180 | 69.013 | 4.839 |
| F5000 | 35.302 | 69.065 | 11.767 | 48.478 | 83.064 | 15.293 | 71.016 | 95.303 | 22.809 |
| G5000 | 33.442 | 65.967 | 11.523 | 43.852 | 77.176 | 15.282 | 83.333 | 97.752 | 36.498 |
