# Supplementary material for: Honey bees (Apis mellifera) modify plant-pollinator network structure, but do not alter wild species’ interactions
Source: PLoS One. 2023 Jul 13;18(7):e0287332. doi: 10.1371/journal.pone.0287332 (PMC10343163; doi:10.1371/journal.pone.0287332)
Supplement: S6 Table — Morphospecies identifications are listed by “[Genus] sp. #”. Some species could not be differentiated between genera, and so both genera are listed along with the epithet “sp”. Specimens listed beside “cf” (confer, meaning compare with) are specimens that were damaged or for which taxonomic keys are insufficient, and these were compared to other specimens to determine identification. Numbers of each (morpho)species are given for each distance from hives, despite the fact that we used honey bee abundance, rather than distance from hive as the predictor variable in our analyses. (DOCX) [file pone.0287332.s011.docx]

Table S6. Identifications of insect pollinators to species-level or morphospecies level. Morphospecies identifications are listed by “[Genus] sp. #”. Some species could not be differentiated between genera, and so both genera are listed along with the epithet “sp”. Specimens listed beside “cf” (confer, meaning compare with) are specimens that were damaged or for which taxonomic keys are insufficient, and these were compared to other specimens to determine identification. Numbers of each (morpho)species are given for each distance from hives, despite the fact that we used honey bee abundance, rather than distance from hive as the predictor variable in our analyses.

| **Group/ Order** | **Family** | **Species** | | **100 m** | | **500 m** | | **5000 m** | | **Total** | |  |  |  |  |  |  |  |  |
| --- | --- | --- | --- | --- | --- | --- | --- | --- | --- | --- | --- | --- | --- | --- | --- | --- | --- | --- | --- |
| **Anthophila** | | | | | | | | | | | | |  |  |  |  |  |  |  |
|  | Andrenidae | *Andrena amphibola* | | 1 | | 0 | | 0 | | 1 | |  |  |  |  |  |  |  |  |
|  | Andrenidae | *Andrena cyanophila* | | 0 | | 2 | | 0 | | 2 | |  |  |  |  |  |  |  |  |
|  | Andrenidae | *Andrena lupinorum* | | 0 | | 1 | | 1 | | 2 | |  |  |  |  |  |  |  |  |
|  | Andrenidae | *Andrena medionitens* | | 0 | | 1 | | 0 | | 1 | |  |  |  |  |  |  |  |  |
|  | Andrenidae | *Andrena peckhami* | | 0 | | 3 | | 6 | | 9 | |  |  |  |  |  |  |  |  |
|  | Andrenidae | *Andrena prunorum* | | 0 | | 1 | | 0 | | 1 | |  |  |  |  |  |  |  |  |
|  | Andrenidae | *Andrena* sp.1 | | 0 | | 1 | | 0 | | 1 | |  |  |  |  |  |  |  |  |
|  | Andrenidae | *Andrena* sp.2 | | 1 | | 0 | | 0 | | 1 | |  |  |  |  |  |  |  |  |
|  | Andrenidae | *Andrena thaspii* | | 4 | | 1 | | 2 | | 7 | |  |  |  |  |  |  |  |  |
|  | Andrenidae | *Panurginus beardsleyi* | | 0 | | 2 | | 0 | | 2 | |  |  |  |  |  |  |  |  |
|  | Andrenidae | *Perdita bruneri* | | 7 | | 12 | | 0 | | 19 | |  |  |  |  |  |  |  |  |
|  | Andrenidae | *Perdita swenki* | | 26 | | 4 | | 0 | | 30 | |  |  |  |  |  |  |  |  |
|  | Apidae | *Apis mellifera* | | 172 | | 108 | | 5 | | 285 | |  |  |  |  |  |  |  |  |
|  | Apidae | *Bombus borealis* | | 7 | | 2 | | 4 | | 13 | |  |  |  |  |  |  |  |  |
|  | Apidae | *Bombus fervidus* | | 1 | | 0 | | 0 | | 1 | |  |  |  |  |  |  |  |  |
|  | Apidae | *Bombus rufocinctus* | | 2 | | 0 | | 0 | | 2 | |  |  |  |  |  |  |  |  |
|  | Apidae | *Bombus ternarius* | | 0 | | 1 | | 1 | | 2 | |  |  |  |  |  |  |  |  |
|  | Apidae | *Epeolus compactus* | | 0 | | 0 | | 2 | | 2 | |  |  |  |  |  |  |  |  |
|  | Apidae | *Epeolus minimus* | | 0 | | 5 | | 4 | | 9 | |  |  |  |  |  |  |  |  |
|  | Apidae | *Holcopasites pulchellus* | | 0 | | 1 | | 0 | | 1 | |  |  |  |  |  |  |  |  |
|  | Apidae | *Melissodes* cf. *coreopsis* | | 3 | | 2 | | 4 | | 9 | |  |  |  |  |  |  |  |  |
|  | Apidae | *Melissodes rivalis* | | 1 | | 0 | | 0 | | 1 | |  |  |  |  |  |  |  |  |
|  | Apidae | *Neolarra pruinosa* | | 2 | | 0 | | 0 | | 2 | |  |  |  |  |  |  |  |  |
|  | Apidae | *Neolarra vigilans* | | 1 | | 0 | | 0 | | 1 | |  |  |  |  |  |  |  |  |
|  | Apidae | *Triepeolus helianthi* | | 0 | | 1 | | 1 | | 2 | |  |  |  |  |  |  |  |  |
|  | Colletidae | *Colletes brevicornis* | | 0 | | 18 | | 23 | | 41 | |  |  |  |  |  |  |  |  |
|  | Colletidae | *Colletes fulgidus* | | 3 | | 0 | | 1 | | 4 | |  |  |  |  |  |  |  |  |
|  | Colletidae | *Colletes hyalinus* | | 28 | | 46 | | 15 | | 89 | |  |  |  |  |  |  |  |  |
|  | Colletidae | *Colletes impunctatus* | | 6 | | 11 | | 6 | | 23 | |  |  |  |  |  |  |  |  |
|  | Colletidae | *Colletes kincaidii* | | 6 | | 11 | | 10 | | 27 | |  |  |  |  |  |  |  |  |
|  | Colletidae | *Colletes* cf. *petalostemonis* | | 2 | | 0 | | 0 | | 2 | |  |  |  |  |  |  |  |  |
|  | Colletidae | *Colletes* cf. *simulans* | | 3 | | 3 | | 1 | | 7 | |  |  |  |  |  |  |  |  |
|  | Colletidae | *Hylaeus annulatus* | | 2 | | 0 | | 0 | | 2 | |  |  |  |  |  |  |  |  |
|  | Colletidae | *Hylaeus mesillae* | | 1 | | 3 | | 0 | | 4 | |  |  |  |  |  |  |  |  |
|  | Halictidae | *Agapostemon femoratus* | | 1 | | 0 | | 0 | | 1 | |  |  |  |  |  |  |  |  |
|  | Halictidae | *Agapostemon texanus* | | 1 | | 0 | | 2 | | 3 | |  |  |  |  |  |  |  |  |
|  | Halictidae | *Agapostemon virescens* | | 1 | | 1 | | 0 | | 2 | |  |  |  |  |  |  |  |  |
|  | Halictidae | *Dufourea maura* | | 0 | | 1 | | 10 | | 11 | |  |  |  |  |  |  |  |  |
|  | Halictidae | *Halictus confusus* | | 5 | | 7 | | 4 | | 16 | |  |  |  |  |  |  |  |  |
|  | Halictidae | *Halictus ligatus* | | 0 | | 2 | | 0 | | 2 | |  |  |  |  |  |  |  |  |
|  | Halictidae | *Halictus rubicundus* | | 1 | | 1 | | 5 | | 7 | |  |  |  |  |  |  |  |  |
|  | Halictidae | *Lasioglossum* cf. *albohirtum* | | 2 | | 4 | | 5 | | 11 | |  |  |  |  |  |  |  |  |
|  | Halictidae | *Lasioglossum egregium* | | 0 | | 1 | | 0 | | 1 | |  |  |  |  |  |  |  |  |
|  | Halictidae | *Lasioglossum hudsoniellum* | | 2 | | 2 | | 1 | | 5 | |  |  |  |  |  |  |  |  |
|  | Halictidae | *Lasioglossum leucozonium* | | 0 | | 2 | | 1 | | 3 | |  |  |  |  |  |  |  |  |
|  | Halictidae | *Lasioglossum paraforbesii* | | 0 | | 0 | | 1 | | 1 | |  |  |  |  |  |  |  |  |
|  | Halictidae | *Lasioglossum pruinosum* | | 1 | | 2 | | 2 | | 5 | |  |  |  |  |  |  |  |  |
|  | Halictidae | *Lasioglossum sagax* | | 2 | | 0 | | 0 | | 2 | |  |  |  |  |  |  |  |  |
|  | Halictidae | *Lasioglossum* sp.1 | | 2 | | 1 | | 2 | | 5 | |  |  |  |  |  |  |  |  |
|  | Halictidae | *Lasioglossum* sp.2 | | 0 | | 0 | | 6 | | 6 | |  |  |  |  |  |  |  |  |
|  | Halictidae | *Lasioglossum* sp.3 | | 0 | | 1 | | 0 | | 1 | |  |  |  |  |  |  |  |  |
|  | Halictidae | *Lasioglossum succinipenne* | | 2 | | 3 | | 1 | | 6 | |  |  |  |  |  |  |  |  |
|  | Halictidae | *Lasiogossum* cf. *rufulipes* | | 6 | | 12 | | 18 | | 36 | |  |  |  |  |  |  |  |  |
|  | Megachilidae | *Anthidium clypeodentatum* | | 0 | | 4 | | 0 | | 4 | |  |  |  |  |  |  |  |  |
|  | Megachilidae | *Coelioxys rufitarsus* | | 0 | | 1 | | 0 | | 1 | |  |  |  |  |  |  |  |  |
|  | Megachilidae | *Hoplitis fulgida* | | 0 | | 1 | | 0 | | 1 | |  |  |  |  |  |  |  |  |
|  | Megachilidae | *Hoplitis pilosifrons* | | 3 | | 4 | | 1 | | 8 | |  |  |  |  |  |  |  |  |
|  | Megachilidae | *Hoplitis producta* | | 5 | | 1 | | 0 | | 6 | |  |  |  |  |  |  |  |  |
|  | Megachilidae | *Hoplitis spoliata* | | 0 | | 1 | | 1 | | 2 | |  |  |  |  |  |  |  |  |
|  | Megachilidae | *Megachile brevis* | | 2 | | 1 | | 2 | | 5 | |  |  |  |  |  |  |  |  |
|  | Megachilidae | *Megachile circumcincta* | | 0 | | 1 | | 0 | | 1 | |  |  |  |  |  |  |  |  |
|  | Megachilidae | *Megachile dentitarsus* | | 1 | | 1 | | 32 | | 34 | |  |  |  |  |  |  |  |  |
|  | Megachilidae | *Megachile frigida* | | 0 | | 1 | | 0 | | 1 | |  |  |  |  |  |  |  |  |
|  | Megachilidae | *Megachile inermis* | | 0 | | 2 | | 0 | | 2 | |  |  |  |  |  |  |  |  |
|  | Megachilidae | *Megachile latimanus* | | 5 | | 9 | | 3 | | 17 | |  |  |  |  |  |  |  |  |
|  | Megachilidae | *Megachile perihirta* | | 1 | | 4 | | 2 | | 7 | |  |  |  |  |  |  |  |  |
|  | Megachilidae | *Megachile rotundata* | | 11 | | 4 | | 0 | | 15 | |  |  |  |  |  |  |  |  |
|  | Megachilidae | *Megachile wheeleri* | | 0 | | 1 | | 0 | | 1 | |  |  |  |  |  |  |  |  |
|  | Megachilidae | *Osmia distincta* | | 5 | | 2 | | 3 | | 10 | |  |  |  |  |  |  |  |  |
|  | Megachilidae | *Osmia integra* | | 0 | | 1 | | 0 | | 1 | |  |  |  |  |  |  |  |  |
|  | Megachilidae | *Osmia simillima* | | 10 | | 9 | | 5 | | 24 | |  |  |  |  |  |  |  |  |
|  | Megachilidae | *Osmia* sp.1 | | 0 | | 1 | | 0 | | 1 | |  |  |  |  |  |  |  |  |
|  | Megachilidae | *Stelis lateralis* | | 0 | | 2 | | 0 | | 2 | |  |  |  |  |  |  |  |  |
| **Coleoptera** | | | | | | | | | | | | |  |  |  |  |  |  |  |
|  | Anthicidae | *Notoxus* cf. *anchora* | | 10 | | 2 | | 10 | | 22 | |  |  |  |  |  |  |  |  |
|  | Chrysomelidae | *Acanthoscelides* sp. | | 0 | | 0 | | 1 | | 1 | |  |  |  |  |  |  |  |  |
|  | Chrysomelidae | *Erynephala* cf. *puncticollis* | | 1 | | 0 | | 0 | | 1 | |  |  |  |  |  |  |  |  |
|  | Cleridae | *Phyllobaenus humeralis* | | 4 | | 25 | | 8 | | 37 | |  |  |  |  |  |  |  |  |
|  | Cleridae | *Trichodes nutalli* | | 1 | | 0 | | 2 | | 3 | |  |  |  |  |  |  |  |  |
|  | Coccinellidae | *Brachiacantha albifrons* | | 0 | | 1 | | 0 | | 1 | |  |  |  |  |  |  |  |  |
|  | Meloidae | *Epicauta ferruginea* | | 0 | | 1 | | 0 | | 1 | |  |  |  |  |  |  |  |  |
|  | Meloidae | *Epicauta pruinosa* | | 0 | | 1 | | 0 | | 1 | |  |  |  |  |  |  |  |  |
|  | Meloidae | *Epicauta puncticollis* | | 0 | | 1 | | 0 | | 1 | |  |  |  |  |  |  |  |  |
|  | Meloidae | *Epicauta subglabra* | | 0 | | 1 | | 5 | | 6 | |  |  |  |  |  |  |  |  |
|  | Melyridae | *Collops vittatus* | | 0 | | 1 | | 0 | | 1 | |  |  |  |  |  |  |  |  |
|  | Melyridae | *Listrus* sp. | | 0 | | 2 | | 0 | | 2 | |  |  |  |  |  |  |  |  |
|  | Mordellidae | *Mordella atrata* | | 0 | | 2 | | 1 | | 3 | |  |  |  |  |  |  |  |  |
|  | Staphylinidae | *Philonthus caerulipennis* | | 1 | | 7 | | 10 | | 18 | |  |  |  |  |  |  |  |  |
| **Diptera** | | | | | | | | | | | | |  |  |  |  |  |  |  |
|  | Acroceridae | *Ogcodes eugonatus* | | 2 | | 0 | | 0 | | 2 | |  |  |  |  |  |  |  |  |
|  | Anthomyiidae | *Adia* sp. | | 0 | | 0 | | 1 | | 1 | |  |  |  |  |  |  |  |  |
|  | Anthomyiidae | *Adia* or *Paregle* sp. | | 0 | | 0 | | 1 | | 1 | |  |  |  |  |  |  |  |  |
|  | Anthomyiidae | *Botanophila* sp. | | 1 | | 0 | | 0 | | 1 | |  |  |  |  |  |  |  |  |
|  | Anthomyiidae | *Delia* sp. | | 0 | | 0 | | 5 | | 5 | |  |  |  |  |  |  |  |  |
|  | Anthomyiidae | *Delia* or *Lasiomma* sp. | | 0 | | 0 | | 1 | | 1 | |  |  |  |  |  |  |  |  |
|  | Anthomyiidae | *Fucellia* sp. | | 0 | | 2 | | 0 | | 2 | |  |  |  |  |  |  |  |  |
|  | Anthomyiidae | *Fucellia* or *Delia* sp. | | 0 | | 1 | | 0 | | 1 | |  |  |  |  |  |  |  |  |
|  | Anthomyiidae | *Hydrophoria* or *Delia* sp. | | 0 | | 1 | | 0 | | 1 | |  |  |  |  |  |  |  |  |
|  | Anthomyiidae | *Pegohylemyia* or *Delia* or *Lasiomma* sp. | | 0 | | 1 | | 1 | | 2 | |  |  |  |  |  |  |  |  |
|  | Anthomyiidae | *Pegohylemyia* or *Delia* sp. | | 0 | | 0 | | 5 | | 5 | |  |  |  |  |  |  |  |  |
|  | Anthomyiidae | *Pegohylemyia* or *Lasiomma* sp. | | 1 | | 0 | | 0 | | 1 | |  |  |  |  |  |  |  |  |
|  | Anthomyiidae | *Pegomya* sp. | | 0 | | 1 | | 1 | | 2 | |  |  |  |  |  |  |  |  |
|  | Anthomyiidae | *Pegomya* or *Adia* sp. | | 0 | | 0 | | 1 | | 1 | |  |  |  |  |  |  |  |  |
|  | Anthomyiidae | *Pegoplata* sp. | | 1 | | 1 | | 1 | | 3 | |  |  |  |  |  |  |  |  |
|  | Anthomyiidae | *Phorbia* or *Hydrophoria* or *Pegohylemyia* sp.*egoplata* sp. | | 1 | | 0 | | 1 | | 0 | |  |  |  |  |  |  |  |  |
|  | Asillidae | *Dicropaltum mesae* | | 0 | | 1 | | 0 | | 1 | |  |  |  |  |  |  |  |  |
|  | Asillidae | *Holopogon albopilosa* | | 1 | | 0 | | 1 | | 2 | |  |  |  |  |  |  |  |  |
|  | Bombyliidae | *Anastoechus barbatus* | | 12 | | 18 | | 4 | | 34 | |  |  |  |  |  |  |  |  |
|  | Bombyliidae | *Anastoechus melanohalteralis* | | 2 | | 6 | | 1 | | 9 | |  |  |  |  |  |  |  |  |
|  | Bombyliidae | *Anthrax picea* | | 1 | | 0 | | 0 | | 1 | |  |  |  |  |  |  |  |  |
|  | Bombyliidae | *Chrysanthrax costata* | | 0 | | 0 | | 1 | | 1 | |  |  |  |  |  |  |  |  |
|  | Bombyliidae | *Geron* sp. | | 0 | | 1 | | 0 | | 1 | |  |  |  |  |  |  |  |  |
|  | Bombyliidae | *Hemipenthes morio* | | 4 | | 2 | | 0 | | 6 | |  |  |  |  |  |  |  |  |
|  | Bombyliidae | *Hemipenthes sinuosa* | | 0 | | 0 | | 1 | | 1 | |  |  |  |  |  |  |  |  |
|  | Bombyliidae | *Poecilanthrax alcyon* | | 0 | | 1 | | 1 | | 2 | |  |  |  |  |  |  |  |  |
|  | Bombyliidae | *Poecilanthrax monticola* | | 0 | | 3 | | 0 | | 3 | |  |  |  |  |  |  |  |  |
|  | Bombyliidae | *Poecilanthrax tegminipennis* | | 0 | | 1 | | 11 | | 12 | |  |  |  |  |  |  |  |  |
|  | Bombyliidae | *Poecilanthrax willistonii* | | 0 | | 2 | | 0 | | 2 | |  |  |  |  |  |  |  |  |
|  | Bombyliidae | *Poecilognathus* sp. | | 0 | | 0 | | 1 | | 1 | |  |  |  |  |  |  |  |  |
|  | Bombyliidae | *Systoechus vulgaris* | | 4 | | 10 | | 2 | | 16 | |  |  |  |  |  |  |  |  |
|  | Bombyliidae | *Villa fulviana* | | 1 | | 1 | | 3 | | 5 | |  |  |  |  |  |  |  |  |
|  | Bombyliidae | *Villa lateralis* | | 2 | | 2 | | 1 | | 5 | |  |  |  |  |  |  |  |  |
|  | Bombyliidae | *Villa lateralis* spgr. | | 0 | | 2 | | 0 | | 2 | |  |  |  |  |  |  |  |  |
|  | Calliphoridae | *Phormia regina* | | 1 | | 0 | | 0 | | 1 | |  |  |  |  |  |  |  |  |
|  | Calliphoridae | *Protophormia terraenovae* | | 0 | | 0 | | 1 | | 1 | |  |  |  |  |  |  |  |  |
|  | Conopidae | *Thecophora occidensis* | | 0 | | 1 | | 0 | | 1 | |  |  |  |  |  |  |  |  |
|  | Conopidae | *Zodion cinereiventre* | | 0 | | 0 | | 2 | | 2 | |  |  |  |  |  |  |  |  |
|  | Conopidae | *Zodion fulvifrons* | | 0 | | 1 | | 1 | | 2 | |  |  |  |  |  |  |  |  |
|  | Conopidae | *Zodion hitchensi* | | 0 | | 1 | | 0 | | 1 | |  |  |  |  |  |  |  |  |
|  | Conopidae | *Zodion lisafyrea* | | 1 | | 0 | | 0 | | 1 | |  |  |  |  |  |  |  |  |
|  | Conopidae | *Zodion* sp. | | 0 | | 1 | | 1 | | 2 | |  |  |  |  |  |  |  |  |
|  | Dolichopodidae | sp. indet. | | 1 | | 0 | | 0 | | 1 | |  |  |  |  |  |  |  |  |
|  | Milichiidae | *Eusiphona mira* | | 0 | | 1 | | 1 | | 2 | |  |  |  |  |  |  |  |  |
|  | Milichiidae | *Pholeomyia* sp. | | 0 | | 1 | | 0 | | 1 | |  |  |  |  |  |  |  |  |
|  | Muscidae | *Haematobia irritans* | | 0 | | 1 | | 0 | | 1 | |  |  |  |  |  |  |  |  |
|  | Muscidae | *Hydrotaea meteorica* | | 1 | | 1 | | 0 | | 2 | |  |  |  |  |  |  |  |  |
|  | Muscidae | *Neomyia cornicina* | | 0 | | 2 | | 2 | | 4 | |  |  |  |  |  |  |  |  |
|  | Pipunculidae | sp. indet. | | 0 | | 0 | | 1 | | 1 | |  |  |  |  |  |  |  |  |
|  | Sarcophagidae | *Ravinia* sp. | | 2 | | 1 | | 6 | | 9 | |  |  |  |  |  |  |  |  |
|  | Sarcophagidae | *Ravinia* or *Arachnidomyia* sp. | | 0 | | 0 | | 1 | | 1 | |  |  |  |  |  |  |  |  |
|  | Sarcophagidae | sp. indet. | | 2 | | 2 | | 3 | | 7 | |  |  |  |  |  |  |  |  |
|  | Sarcophagidae | *Senotainia* sp. | | 1 | | 9 | | 1 | | 11 | |  |  |  |  |  |  |  |  |
|  | Sarcophagidae | *Senotainia* or *Macronychia* sp. | | 0 | | 2 | | 0 | | 2 | |  |  |  |  |  |  |  |  |
|  | Sarcophagidae | *Sphixapata trilineata* | | 0 | | 4 | | 0 | | 4 | |  |  |  |  |  |  |  |  |
|  | Sarcophagidae | *Udamopyga niagarana* | | 0 | | 1 | | 0 | | 1 | |  |  |  |  |  |  |  |  |
|  | Stratiomyidae | *Nemotelus* or *Camptopelta* sp. | | 1 | | 0 | | 0 | | 1 | |  |  |  |  |  |  |  |  |
|  | Syrphidae | *Cheilosia* sp. | | 0 | | 0 | | 2 | | 2 | |  |  |  |  |  |  |  |  |
|  | Syrphidae | *Copestylum marginatum* | | 1 | | 0 | | 0 | | 1 | |  |  |  |  |  |  |  |  |
|  | Syrphidae | *Copestylum* sp. | | 0 | | 1 | | 0 | | 1 | |  |  |  |  |  |  |  |  |
|  | Syrphidae | *Eristalis dimidiata* | | 0 | | 0 | | 1 | | 1 | |  |  |  |  |  |  |  |  |
|  | Syrphidae | *Eristalis hirta* | | 1 | | 0 | | 1 | | 2 | |  |  |  |  |  |  |  |  |
|  | Syrphidae | *Eristalis stipator* | | 15 | | 16 | | 14 | | 45 | |  |  |  |  |  |  |  |  |
|  | Syrphidae | *Eristalis tenax* | | 0 | | 0 | | 1 | | 1 | |  |  |  |  |  |  |  |  |
|  | Syrphidae | *Eupeodes* sp. | | 0 | | 2 | | 0 | | 2 | |  |  |  |  |  |  |  |  |
|  | Syrphidae | *Eupeodes volucris* | | 2 | | 1 | | 1 | | 4 | |  |  |  |  |  |  |  |  |
|  | Syrphidae | *Helophilus hybridus* | | 3 | | 1 | | 1 | | 5 | |  |  |  |  |  |  |  |  |
|  | Syrphidae | *Helophilus latifrons* | | 3 | | 7 | | 4 | | 14 | |  |  |  |  |  |  |  |  |
|  | Syrphidae | *Helophilus obscurus* | | 1 | | 1 | | 0 | | 2 | |  |  |  |  |  |  |  |  |
|  | Syrphidae | *Lapposyrphus lapponicus* | | 0 | | 0 | | 1 | | 1 | |  |  |  |  |  |  |  |  |
|  | Syrphidae | *Paragus haemorrhous* | | 6 | | 10 | | 9 | | 25 | |  |  |  |  |  |  |  |  |
|  | Syrphidae | *Paragus* sp. | | 4 | | 13 | | 2 | | 19 | |  |  |  |  |  |  |  |  |
|  | Syrphidae | *Sphaerophoria bifurcata* | | 2 | | 2 | | 0 | | 4 | |  |  |  |  |  |  |  |  |
|  | Syrphidae | *Sphaerophoria contigua* | | 1 | | 3 | | 2 | | 6 | |  |  |  |  |  |  |  |  |
|  | Syrphidae | *Sphaerophoria philanthus* | | 8 | | 13 | | 4 | | 25 | |  |  |  |  |  |  |  |  |
|  | Syrphidae | *Sphaerophoria* sp. | | 1 | | 0 | | 0 | | 1 | |  |  |  |  |  |  |  |  |
|  | Syrphidae | *Syritta pipiens* | | 1 | | 0 | | 0 | | 1 | |  |  |  |  |  |  |  |  |
|  | Syrphidae | *Syrphus vitripennis* | | 0 | | 0 | | 1 | | 1 | |  |  |  |  |  |  |  |  |
|  | Syrphidae | *Toxomerus marginatus* | | 6 | | 7 | | 16 | | 29 | |  |  |  |  |  |  |  |  |
|  | Syrphidae | *Trichopsomyia apisaon* | | 1 | | 0 | | 0 | | 1 | |  |  |  |  |  |  |  |  |
|  | Tachinidae | *Aphria ocypterata* | | 5 | | 3 | | 1 | | 9 | |  |  |  |  |  |  |  |  |
|  | Tachinidae | *Archytas californiae* | | 0 | | 0 | | 1 | | 1 | |  |  |  |  |  |  |  |  |
|  | Tachinidae | *Belvosia canadensis* | | 1 | | 0 | | 0 | | 1 | |  |  |  |  |  |  |  |  |
|  | Tachinidae | *Besseria brevipennis* | | 0 | | 1 | | 4 | | 5 | |  |  |  |  |  |  |  |  |
|  | Tachinidae | *Chaetocrania antennalis* | | 0 | | 1 | | 0 | | 1 | |  |  |  |  |  |  |  |  |
|  | Tachinidae | *Cylindromyia californica* | | 0 | | 3 | | 0 | | 3 | |  |  |  |  |  |  |  |  |
|  | Tachinidae | *Cylindromyia decora* | | 0 | | 1 | | 2 | | 3 | |  |  |  |  |  |  |  |  |
|  | Tachinidae | *Dinera grisescens* | | 1 | | 1 | | 0 | | 2 | |  |  |  |  |  |  |  |  |
|  | Tachinidae | *Estheria* sp. | | 3 | | 4 | | 0 | | 7 | |  |  |  |  |  |  |  |  |
|  | Tachinidae | *Exorista* sp. | | 1 | | 0 | | 0 | | 1 | |  |  |  |  |  |  |  |  |
|  | Tachinidae | *Gonia* sp. | | 0 | | 6 | | 0 | | 6 | |  |  |  |  |  |  |  |  |
|  | Tachinidae | *Gymnoclytia immaculata* | | 0 | | 1 | | 0 | | 1 | |  |  |  |  |  |  |  |  |
|  | Tachinidae | *Lydina americana spcomplex* | | 1 | | 0 | | 1 | | 2 | |  |  |  |  |  |  |  |  |
|  | Tachinidae | *Panzeria fasciventris* | | 0 | | 1 | | 0 | | 1 | |  |  |  |  |  |  |  |  |
|  | Tachinidae | *Peleteria clara* | | 4 | | 18 | | 11 | | 33 | |  |  |  |  |  |  |  |  |
|  | Tachinidae | *Peleteria* sp. | | 1 | | 3 | | 2 | | 6 | |  |  |  |  |  |  |  |  |
|  | Tachinidae | *Peleteria* or *Oxydosphyria* sp. | | 0 | | 3 | | 0 | | 3 | |  |  |  |  |  |  |  |  |
|  | Tachinidae | *Peleteria* or *Sphyromyia* sp. | | 0 | | 0 | | 1 | | 1 | |  |  |  |  |  |  |  |  |
|  | Tachinidae | *Ptilodexia rufipennis* | | 1 | | 0 | | 4 | | 5 | |  |  |  |  |  |  |  |  |
|  | Tachinidae | *Siphona medialis* | | 0 | | 1 | | 0 | | 1 | |  |  |  |  |  |  |  |  |
|  | Tachinidae | *Spallanzania hebes* | | 1 | | 0 | | 0 | | 1 | |  |  |  |  |  |  |  |  |
|  | Tachinidae | *Spallanzania hesperidarum* | | 0 | | 2 | | 1 | | 3 | |  |  |  |  |  |  |  |  |
|  | Tachinidae | *Tachina* sp. | | 1 | | 0 | | 0 | | 1 | |  |  |  |  |  |  |  |  |
|  | Tachinidae | *Tachinidae* sp.1 | | 1 | | 0 | | 0 | | 1 | |  |  |  |  |  |  |  |  |
|  | Tachinidae | *Tachinidae* sp.2 | | 0 | | 1 | | 0 | | 1 | |  |  |  |  |  |  |  |  |
|  | Tachinidae | *Tachinidae* sp.3 | | 2 | | 0 | | 0 | | 2 | |  |  |  |  |  |  |  |  |
|  | Tachinidae | *Tachinidae* sp.4 | | 1 | | 0 | | 0 | | 1 | |  |  |  |  |  |  |  |  |
|  | Tachinidae | *Tachinidae* sp.6 | | 1 | | 0 | | 0 | | 1 | |  |  |  |  |  |  |  |  |
|  | Tachinidae | *Tachinidae* sp.8 | | 0 | | 0 | | 1 | | 1 | |  |  |  |  |  |  |  |  |
|  | Tachinidae | *Tachinidae* sp.9 | | 0 | | 1 | | 0 | | 1 | |  |  |  |  |  |  |  |  |
|  | Therevidae | *Ozodiceromya platancala* | | 0 | | 1 | | 0 | | 1 | |  |  |  |  |  |  |  |  |
|  | Ulidiidae | sp. indet. | | 1 | | 1 | | 0 | | 2 | |  |  |  |  |  |  |  |  |
|  | Ulidiidae | sp. indet. | | 1 | | 1 | | 0 | | 2 | |  |  |  |  |  |  |  |  |
| **Papilionoidea** | | | | | | | | | | | | | Hesperiidae | | *Hesperia assiniboia* | 2 | 19 | 55 | 76 |
|  | Hesperiidae | *Oarisma garita* | | 3 | | 3 | | 0 | | 6 | |  |  |  |  |  |  |  |  |
|  | Hesperiidae | *Pyrgus communis* | | 0 | | 4 | | 0 | | 4 | |  |  |  |  |  |  |  |  |
|  | Lycaenidae | *Glaucopsyche lygdamus* | | 1 | | 0 | | 0 | | 1 | |  |  |  |  |  |  |  |  |
|  | Lycaenidae | *Icaricia saepiolus* | | 3 | | 4 | | 0 | | 7 | |  |  |  |  |  |  |  |  |
|  | Lycaenidae | *Lycaena dione* | | 0 | | 0 | | 1 | | 1 | |  |  |  |  |  |  |  |  |
|  | Lycaenidae | *Plebejus melissa* | | 7 | | 0 | | 0 | | 7 | |  |  |  |  |  |  |  |  |
|  | Nymphalidae | *Cercyonis pegala* | | 2 | | 4 | | 3 | | 9 | |  |  |  |  |  |  |  |  |
|  | Nymphalidae | *Coenonympha california* | | 2 | | 0 | | 0 | | 2 | |  |  |  |  |  |  |  |  |
|  | Nymphalidae | *Phyciodes tharos* | | 0 | | 2 | | 1 | | 3 | |  |  |  |  |  |  |  |  |
|  | Nymphalidae | *Speyeria aphrodite* | | 0 | | 0 | | 1 | | 1 | |  |  |  |  |  |  |  |  |
|  | Nymphalidae | *Speyeria callippe* | | 1 | | 0 | | 0 | | 1 | |  |  |  |  |  |  |  |  |
|  | Pieridae | *Colias alexandra* | | 1 | | 0 | | 0 | | 1 | |  |  |  |  |  |  |  |  |
|  | Pieridae | *Colias philodice* | | 2 | | 5 | | 2 | | 9 | |  |  |  |  |  |  |  |  |
|  | Pieridae | *Pontia occidentalis* | | 1 | | 0 | | 1 | | 2 | |  |  |  |  |  |  |  |  |
|  | Pieridae | *Pontia occidentalis* | | 1 | | 0 | | 1 | | 2 | |  |  |  |  |  |  |  |  |
| **Non-Papilionoidea Lepidoptera – “Moths”** | | | | | | | | | | | | | Coleophoridae | | *Coleophora trifolii* | 1 | 0 | 0 | 1 |
|  | Crambidae | *Pediasia dorsipunctellus* | | 0 | | 0 | | 1 | | 1 | |  |  |  |  |  |  |  |  |
|  | Gelechiidae | sp. indet. | | 1 | | 0 | | 0 | | 1 | |  |  |  |  |  |  |  |  |
|  | Gelechiidae | *Gnorimoschema* sp. | | 0 | | 0 | | 1 | | 1 | |  |  |  |  |  |  |  |  |
|  | Geometridae | sp. indet. | | 1 | | 0 | | 0 | | 1 | |  |  |  |  |  |  |  |  |
|  | Noctuidae | *Euxoa ochrogaster* | | 0 | | 0 | | 1 | | 1 | |  |  |  |  |  |  |  |  |
|  | Noctuidae | *Euxoa* sp. | | 0 | | 0 | | 1 | | 1 | |  |  |  |  |  |  |  |  |
|  | Noctuidae | *Lacinipolia lorea* | | 1 | | 2 | | 0 | | 3 | |  |  |  |  |  |  |  |  |
|  | Noctuidae | Noctuinae sp. indet. | | 0 | | 0 | | 1 | | 1 | |  |  |  |  |  |  |  |  |
|  | Noctuidae | *Ponometia tortricina* | | 1 | | 0 | | 1 | | 2 | |  |  |  |  |  |  |  |  |
|  | Noctuidae | *Schinia villosa* | | 1 | | 0 | | 0 | | 1 | |  |  |  |  |  |  |  |  |
|  | Scythrididae | *Landryia scintillifera* | | 0 | | 1 | | 1 | | 2 | |  |  |  |  |  |  |  |  |
|  | Scythrididae | *Rhamphura ochristriata* | | 0 | | 1 | | 1 | | 2 | |  |  |  |  |  |  |  |  |
|  | Scythrididae | *Scythris eboracensis* | | 0 | | 1 | | 3 | | 4 | |  |  |  |  |  |  |  |  |
|  | Scythrididae | *Scythris inspersella* | | 0 | | 0 | | 2 | | 2 | |  |  |  |  |  |  |  |  |
|  | Tortricidae | *Hystrichophora ochreicostana* | | 1 | | 0 | | 0 | | 1 | |  |  |  |  |  |  |  |  |
|  | Tortricidae | *Hystrichophora ochreicostana* | | 1 | | 0 | | 0 | | 1 | |  |  |  |  |  |  |  |  |
| **Formicoidea** | | | | | | | | | | | | | Formicidae | | *Formica canadensis* | 2 | 1 | 1 | 4 |
|  | Formicidae | *Formica lasioides* | | 18 | | 9 | | 4 | | 31 | |  |  |  |  |  |  |  |  |
|  | Formicidae | *Formica montana* | | 1 | | 0 | | 1 | | 2 | |  |  |  |  |  |  |  |  |
|  | Formicidae | *Formica neogagates* | | 0 | | 5 | | 0 | | 5 | |  |  |  |  |  |  |  |  |
|  | Formicidae | *Formica obscuripes* | | 13 | | 5 | | 0 | | 18 | |  |  |  |  |  |  |  |  |
|  | Formicidae | *Formica oreas* | | 1 | | 26 | | 0 | | 27 | |  |  |  |  |  |  |  |  |
|  | Formicidae | *Formica podzolica* | | 7 | | 3 | | 0 | | 10 | |  |  |  |  |  |  |  |  |
|  | Formicidae | *Formica ravida* | | 0 | | 1 | | 0 | | 1 | |  |  |  |  |  |  |  |  |
|  | Formicidae | *Myrmica fracticornis* | | 0 | | 0 | | 1 | | 1 | |  |  |  |  |  |  |  |  |
|  | Formicidae | *Myrmica* sp. | | 1 | | 0 | | 0 | | 1 | |  |  |  |  |  |  |  |  |
|  | Formicidae | *Tapinoma sessile* | | 0 | | 4 | | 0 | | 4 | |  |  |  |  |  |  |  |  |
|  | Formicidae | *Tapinoma sessile* | | 0 | | 4 | | 0 | | 4 | |  |  |  |  |  |  |  |  |
| **Hemiptera** | | | | | | | | | | | | | Alydidae | | *Alydus* sp. | 4 | 1 | 2 | 7 |
|  | Cicadellidae | *Cicadellidae* sp.1 | | 1 | | 1 | | 1 | | 3 | |  |  |  |  |  |  |  |  |
|  | Cicadellidae | *Cicadellidae* sp.2 | | 0 | | 0 | | 1 | | 1 | |  |  |  |  |  |  |  |  |
|  | Miridae | *Lopidea* sp. | | 1 | | 0 | | 0 | | 1 | |  |  |  |  |  |  |  |  |
|  | Miridae | *Lygus* sp. | | 0 | | 0 | | 2 | | 2 | |  |  |  |  |  |  |  |  |
|  | Reduviidae | *Phymata americana* | | 4 | | 5 | | 5 | | 14 | |  |  |  |  |  |  |  |  |
|  | Rhyparochromidae | sp. indet. | | 0 | | 1 | | 0 | | 1 | |  |  |  |  |  |  |  |  |
|  | Rhyparochromidae | sp. indet. | | 0 | | 1 | | 0 | | 1 | |  |  |  |  |  |  |  |  |
| **Aculeata (non-Anthophila & non-Formicoidea) – “Wasps”** | | | | | | | | | | | | | Braconidae | | *Braconidae* sp. | 1 | 1 | 0 | 2 |
|  | Braconidae | *Chelonus annulipes* | | 1 | | 2 | | 0 | | 3 | |  |  |  |  |  |  |  |  |
|  | Braconidae | *Chelonus* sp. | | 0 | | 1 | | 0 | | 1 | |  |  |  |  |  |  |  |  |
|  | Crabronidae | *Belomicrus* sp. | | 0 | | 0 | | 3 | | 3 | |  |  |  |  |  |  |  |  |
|  | Crabronidae | *Cerceris deserta* | | 0 | | 1 | | 0 | | 1 | |  |  |  |  |  |  |  |  |
|  | Crabronidae | *Cerceris nigrescens* | | 2 | | 0 | | 0 | | 2 | |  |  |  |  |  |  |  |  |
|  | Crabronidae | *Ectemnius arcuatus* | | 0 | | 1 | | 0 | | 1 | |  |  |  |  |  |  |  |  |
|  | Crabronidae | *Ectemnius rufifemur* | | 1 | | 0 | | 5 | | 6 | |  |  |  |  |  |  |  |  |
|  | Crabronidae | *Eucerceris tricolor* | | 1 | | 0 | | 0 | | 1 | |  |  |  |  |  |  |  |  |
|  | Crabronidae | *Gorytes simillimus* | | 0 | | 4 | | 0 | | 4 | |  |  |  |  |  |  |  |  |
|  | Crabronidae | *Nysson recticornis* | | 0 | | 1 | | 1 | | 2 | |  |  |  |  |  |  |  |  |
|  | Crabronidae | *Philanthus bilunatus* | | 0 | | 2 | | 0 | | 2 | |  |  |  |  |  |  |  |  |
|  | Crabronidae | *Tachysphex aequalis* | | 0 | | 1 | | 1 | | 2 | |  |  |  |  |  |  |  |  |
|  | Crabronidae | *Tachysphex pompilliformis* | | 0 | | 1 | | 1 | | 2 | |  |  |  |  |  |  |  |  |
|  | Cynipidae | *Diplolepsis rosae* | | 0 | | 3 | | 0 | | 3 | |  |  |  |  |  |  |  |  |
|  | Ichneumonidae | *Himerta* sp.1 | | 0 | | 0 | | 1 | | 1 | |  |  |  |  |  |  |  |  |
|  | Ichneumonidae | *Himerta* sp.2 | | 0 | | 1 | | 0 | | 1 | |  |  |  |  |  |  |  |  |
|  | Ichneumonidae | sp. indet. | | 1 | | 0 | | 0 | | 1 | |  |  |  |  |  |  |  |  |
|  | Ichneumonidae | *Ophion* sp. | | 0 | | 0 | | 1 | | 1 | |  |  |  |  |  |  |  |  |
|  | Perilampidae | *Chrysolampus schwarzi* | | 0 | | 1 | | 0 | | 1 | |  |  |  |  |  |  |  |  |
|  | Perilampidae | *Perilampus hyalinus* | | 0 | | 0 | | 3 | | 3 | |  |  |  |  |  |  |  |  |
|  | Pompilidae | *Arachnospila michiganensis* | | 0 | | 1 | | 0 | | 1 | |  |  |  |  |  |  |  |  |
|  | Pompilidae | *Episyron oregon* | | 0 | | 0 | | 1 | | 1 | |  |  |  |  |  |  |  |  |
|  | Pompilidae | *Evagetes crassicornis* | | 1 | | 0 | | 0 | | 1 | |  |  |  |  |  |  |  |  |
|  | Sphecidae | *Ammophila harti* | | 0 | | 2 | | 2 | | 4 | |  |  |  |  |  |  |  |  |
|  | Sphecidae | *Prionyx atratus* | | 0 | | 0 | | 1 | | 1 | |  |  |  |  |  |  |  |  |
|  | Sphecidae | *Sphex ichneumoneus* | | 1 | | 0 | | 0 | | 1 | |  |  |  |  |  |  |  |  |
|  | Vespidae | *Ancistrocerus* sp. | | 0 | | 1 | | 0 | | 1 | |  |  |  |  |  |  |  |  |
|  | Vespidae | *Euodynerus crypticus* | | 0 | | 0 | | 1 | | 1 | |  |  |  |  |  |  |  |  |
|  | Vespidae | *Euodynerus leucomelas* | | 1 | | 0 | | 0 | | 1 | |  |  |  |  |  |  |  |  |
|  | Vespidae | *Paranstrocerus* or *Stenodynerus* sp. | | 0 | | 0 | | 1 | | 1 | |  |  |  |  |  |  |  |  |
|  | Vespidae | *Stenodynerus anormis* | | 2 | | 2 | | 2 | | 6 | |  |  |  |  |  |  |  |  |
|  |  |  | |  | | **586** | | **726** | | **502** | |  |  |  |  |  |  |  |  |
|  |  |  |  | |  | |  | |  | |  | | |  |  |  |  |  |  |
