# Supplementary material for: Honey bees (Apis mellifera) modify plant-pollinator network structure, but do not alter wild species’ interactions
Source: PLoS One. 2023 Jul 13;18(7):e0287332. doi: 10.1371/journal.pone.0287332 (PMC10343163; doi:10.1371/journal.pone.0287332)
Supplement: S7 Table — (DOCX) [file pone.0287332.s012.docx]

Table S7. Identifications of flowering species from each distance from honey bee hives to species level.

| **Family** | **Species** | **100 m** | **500 m** | **5000 m** | **Total** |
| --- | --- | --- | --- | --- | --- |
| Asteraceae | *Achillea millefolium* | 68 | 183 | 115 | 366 |
| Asteraceae | *Antennaria microphylla* | 0 | 0 | 47 | 47 |
| Asteraceae | *Cirsium arvense* | 0 | 0 | 4 | 4 |
| Asteraceae | *Cirsium undulatum* | 18 | 21 | 2 | 41 |
| Asteraceae | *Erigeron philadelphicus* | 9 | 27 | 6 | 42 |
| Asteraceae | *Erigeron speciosus* | 0 | 0 | 2 | 2 |
| Asteraceae | *Grindelia squarrosa* | 15 | 346 | 178 | 539 |
| Asteraceae | *Gutierrezia sarothrae* | 0 | 7 | 0 | 7 |
| Asteraceae | *Happlopappus spinulosus* | 0 | 2 | 0 | 2 |
| Asteraceae | *Heterotheca villosa* | 117 | 281 | 47 | 445 |
| Asteraceae | *Liatris punctata* | 30 | 84 | 197 | 311 |
| Asteraceae | *Lygodesmia juncea* | 0 | 72 | 4 | 76 |
| Asteraceae | *Mulgedium pulchellum* | 0 | 1 | 0 | 1 |
| Asteraceae | *Solidago canadensis* | 96 | 91 | 47 | 234 |
| Asteraceae | *Solidago missouriensis* | 8 | 67 | 8 | 83 |
| Asteraceae | *Sonchus arvensis* | 33 | 34 | 3 | 70 |
| Asteraceae | *Sonchus oleraceus* | 0 | 0 | 1 | 1 |
| Asteraceae | *Taraxacum officinale* | 52 | 0 | 1 | 53 |
| Asteraceae | *Trogopogon dubius* | 1 | 0 | 4 | 5 |
| Boraginaceae | *Lithospermum incisum* | 28 | 61 | 0 | 89 |
| Brassicaceae | *Descurainia sophia* | 36 | 4 | 0 | 40 |
| Brassicaceae | *Erysimum inconspicuum* | 3 | 2 | 4 | 9 |
| Cactaceae | *Escobaria vivipara* | 1 | 3 | 4 | 8 |
| Campanulaceae | *Campanula rotundifolia* | 18 | 151 | 526 | 695 |
| Caprifoliaceae | *Symphoricarpos occidentalis* | 368 | 443 | 367 | 1178 |
| Caprifoliaceae | *Symphyotrichum falcatum* | 30 | 75 | 140 | 245 |
| Caryophyllaceae | *Cerastium arvense* | 0 | 9 | 7 | 16 |
| Elaeagnaceae | *Elaeagnus commutata* | 7 | 0 | 0 | 7 |
| Fabaceae | *Astragalus adsurgens* | 0 | 6 | 0 | 6 |
| Fabaceae | *Astragalus cicer* | 245 | 164 | 118 | 527 |
| Fabaceae | *Astragalus flexuosus* | 0 | 0 | 2 | 2 |
| Fabaceae | *Astragalus striatus* | 20 | 12 | 9 | 41 |
| Fabaceae | *Dalea purpurea* | 5 | 21 | 0 | 26 |
| Fabaceae | *Glycyrrhiza lepidota* | 67 | 497 | 413 | 977 |
| Fabaceae | *Medicago sativa* | 34 | 0 | 0 | 34 |
| Fabaceae | *Melilotus albus* | 66 | 21 | 0 | 87 |
| Fabaceae | *Melilotus officinalis* | 0 | 1 | 0 | 1 |
| Fabaceae | *Thermopsis rhombifolia* | 117 | 48 | 0 | 165 |
| Fabaceae | *Vicia americana* | 0 | 7 | 0 | 7 |
| Liliaceae | *Allium textile* | 68 | 2 | 0 | 70 |
| Onagraceae | *Oenothera nuttallii* | 0 | 5 | 1 | 6 |
| Onagraceae | *Oenothera suffrutescens* | 0 | 11 | 1 | 12 |
| Rosaceae | *Potentilla arguta* | 0 | 2 | 1 | 3 |
| Rosaceae | *Potentilla concinna* | 3 | 85 | 0 | 88 |
| Rosaceae | *Potentilla pensylvanica* | 0 | 1 | 0 | 1 |
| Rosaceae | *Rosa arkansana* | 14 | 14 | 10 | 38 |
| Rosaceae | *Rosa woodsii* | 16 | 8 | 5 | 29 |
| Santalaceae | *Comandra umbellata* | 81 | 62 | 0 | 143 |
|  |  | **1674** | **2931** | **2274** | **6879** |
