# Supplementary material for: Honey bees (Apis mellifera) modify plant-pollinator network structure, but do not alter wild species’ interactions
Source: PLoS One. 2023 Jul 13;18(7):e0287332. doi: 10.1371/journal.pone.0287332 (PMC10343163; doi:10.1371/journal.pone.0287332)
Supplement: S8 Table — Shaded cells denote networks that had too few species for some network metrics to be meaningful, so these replicates were excluded from analysis for the metrics: generality, vulnerability, plant and pollinator niche overlap, and nestedness. Mean network size and range in network size are shown for each dataset. (DOCX) [file pone.0287332.s013.docx]

**Table S8.** Network size (number of interactions) for each network that was analyzed from each transect. Shaded cells denote networks that had too few species for some network metrics to be meaningful, so these replicates were excluded from analysis for the metrics: generality, vulnerability, plant and pollinator niche overlap, and nestedness. Mean network size and range in network size are shown for each dataset.

|  | **Network Size** | | | |
| --- | --- | --- | --- | --- |
| **Transect** | Full Season All Taxa | Mid-season All Taxa | Full Season Bees Only | Without-honey bees Full Taxa |
| A100 | 118 | 39 | 67 | 110 |
| A500 | 146 | 70 | 107 | 120 |
| A5000 | 101 | 46 | 50 | 101 |
| B100 | 55 | 22 | 21 | 48 |
| B500 | 103 | 25 | 27 | 103 |
| B5000 | 78 | 20 | 28 | 76 |
| C100 | 164 | 78 | 98 | 110 |
| C500 | 145 | 61 | 42 | 125 |
| C5000 | 122 | 54 | 25 | 119 |
| D100 | 126 | 79 | 91 | 57 |
| D500 | 123 | 41 | 69 | 91 |
| D5000 | 53 | 15 | 28 | 53 |
| E100 | 18 | 0 | 7 | 15 |
| E500 | 147 | 72 | 72 | 124 |
| E5000 | 74 | 30 | 26 | 74 |
| F100 | 105 | 46 | 64 | 74 |
| F500 | 62 | 25 | 14 | 55 |
| F5000 | 25 | 23 | 21 | 25 |
| G5000 | 49 | 21 | 15 | 49 |
| **Mean Network Size** | 95.5 | 40.4 | 45.9 | 80.5 |
| **Range in Network Size** | 18 to 164 | 0 to 79 | 7 to 107 | 15 to 125 |
