# Supplementary material for: Honey bees (Apis mellifera) modify plant-pollinator network structure, but do not alter wild species’ interactions
Source: PLoS One. 2023 Jul 13;18(7):e0287332. doi: 10.1371/journal.pone.0287332 (PMC10343163; doi:10.1371/journal.pone.0287332)
Supplement: S9 Table — There was a general increase in each network metric with distance to hives, with the exceptions of plant niche overlap, functional complementarity, and generality. Bolded values indicate results that were unexpected based on the literature and/or expectations if competition between honey bees and wild pollinators is occurring. Positive (+) indicates a positive correlation between honey bee abundance and the metric, while negative (-) indicates a negative correlation, with distance from hives as a proxy for honey bee abundance (even though this was not a perfect proxy, as can be seen in S1 Fig). (DOCX) [file pone.0287332.s014.docx]

**Table S9**. Comparison of the full season meta-network metrics calculated for networks created by pooling data from all transects at each distance from hives, over the whole season. There was a general increase in each network metric with distance to hives, with the exceptions of plant niche overlap, functional complementarity, and generality. Bolded values indicate results that were unexpected based on the literature and/or expectations if competition between honey bees and wild pollinators is occurring. Positive (+) indicates a positive correlation between honey bee abundance and the metric, while negative (-) indicates a negative correlation, with distance from hives as a proxy for honey bee abundance (even though this was not a perfect proxy, as can be seen in Fig S1).

|  | Distance From Honey Bee Hives | | |  |
| --- | --- | --- | --- | --- |
| Network Metric | 100 m | 500 m | 5000 m |  |
| **Generality** | **2.29** | **2.94** | **2.06** | **+** |
| Vulnerability | 9.92 | 12.45 | 14.44 | - |
| Pollinator niche overlap | 0.12 | 0.09 | 0.14 | - |
| **Plant niche overlap** | **0.06** | **0.07** | **0.04** | **+** |
| Pollinator functional complementarity | 476.78 | 427.39 | 339.66 | + |
| Plant functional complementarity | 411.43 | 355.71 | 255.55 | + |
| **Weighted nestedness** | **4.21** | **4.15** | **5.97** | **-** |
| Modularity | 0.59 | 0.58 | 0.63 | - |
| **Weighted connectance** | **0.04** | **0.04** | **0.05** | **-** |
| Link density | 6.11 | 7.69 | 8.25 | - |
| **Interaction strength asymmetry** | **0.18** | **0.13** | **0.23** | **-** |
| Interaction evenness | 0.54 | 0.60 | 0.61 | - |
