# Supplementary material for: Honey bees (Apis mellifera) modify plant-pollinator network structure, but do not alter wild species’ interactions
Source: PLoS One. 2023 Jul 13;18(7):e0287332. doi: 10.1371/journal.pone.0287332 (PMC10343163; doi:10.1371/journal.pone.0287332)
Supplement: S10 Table — (DOCX) [file pone.0287332.s015.docx]

**Table S10.** Historical data obtained from the Alberta Climate Information Service (ACIS) Verger AGCM weather station in Southern Alberta, located on the University of Alberta’s Mattheis Research Ranch.

|  | **Month** | **Time Period** | | |
| --- | --- | --- | --- | --- |
|  |  | **2018** | **2019** | **20-Year Average (1999-2019)** |
| Average Maximum Air Temperature (℃) | June | 23.92 | 23.68 | 22.26 |
|  | July | 27.20 | 25.87 | 26.62 |
|  | August | 26.06 | 24.24 | 25.54 |
| Average Precipitation (mm) | June | 51.65 | 26.77 | 74.61 |
|  | July | 42.78 | 39.39 | 46.49 |
|  | August | 28.36 | 43.50 | 44.67 |
