# Supplementary material for: Honey bees (Apis mellifera) modify plant-pollinator network structure, but do not alter wild species’ interactions
Source: PLoS One. 2023 Jul 13;18(7):e0287332. doi: 10.1371/journal.pone.0287332 (PMC10343163; doi:10.1371/journal.pone.0287332)

PLOS ONE

Honey bees (Apis mellifera) modify plant-pollinator network structure, but do not alter wild species' interactions

--Manuscript Draft--

**Manuscript Number:**	PONE-D-23-06336
**Article Type:**	Research Article
**Full Title:**	Honey bees (Apis mellifera) modify plant-pollinator network structure, but do not alter wild species' interactions
**Short Title:**	Managed honey bees in wild plant-pollinator networks
**Corresponding Author:**	Sydney Worthy, MSc.<br>University of Alberta<br>Edmonton, Alberta CANADA
**Keywords:**	Apis mellifera; ecological network; grassland; honey bee; mutualistic network; native pollinator; plant-pollinator network interactions; wild pollinator
**Abstract:**	Honey bees (Apis mellifera) are widely used for honey production and crop pollination, raising concern for wild pollinators, as honey bees may compete with wild pollinators for floral resources. The first sign of competition, before changes appear in wild pollinator abundance or diversity, may be changes to wild pollinator interactions with plants. Such changes for a community can be measured by looking at changes to metrics of resource use overlap in plant-pollinator interaction networks. Studies of honey bee effects on plant-pollinator networks have usually not distinguished whether honey bees alter wild pollinator interactions, or if they merely alter total network structure by adding their own interactions. To test this question, we experimentally introduced honey bees to a Canadian grassland and measured plant-pollinator interactions at varying distances from the introduced hives. We found that honey bees increased the network metrics of pollinator and plant functional complementarity and decreased interaction evenness. However, in networks constructed from just wild pollinator interactions, honey bee abundance did not affect any of the metrics calculated. Thus, all network structural changes to the full network (including honey bee interactions) were due only to honey bee-plant interactions, and not to honey bees causing changes in wild pollinator-plant interactions. Given widespread and increasing use of honey bees, it is important to establish whether they affect wild pollinator communities. Our results suggest that honey bees did not alter wild pollinator foraging patterns in this system, even in a year that was drier than the 20-year average.
**Order of Authors:**	Sydney Worthy, MSc. Conservation Biology
	John Acorn
	Carol Frost
**Additional Information:**	
**Question**	**Response**
**Financial Disclosure**<br><br>Enter a financial disclosure statement that describes the sources of funding for the work included in this submission. Review the submission guidelines for detailed requirements. View published research articles from *PLOS ONE* for specific examples.<br><br>This statement is required for submission and **will appear in the published article** if	S.H.W. was provided funding by the University of Alberta's Rangeland Research Institute and awarded funding by the Alberta Conservation Association. There were no award or grant numbers associated with these grants.<br><br>RRI - https://rri.ualberta.ca/<br>ACA - https://www.ab-conservation.com/<br><br>The funders had no role in study design, data collection and analysis, decision to publish, or preparation of the manuscript.

Powered by Editorial Manager® and ProduXion Manager® from Aries Systems Corporation

the submission is accepted. Please make sure it is accurate.<br><br>**Unfunded studies**<br>Enter: *The author(s) received no specific funding for this work.*<br><br>**Funded studies**<br>Enter a statement with the following details:<br>• Initials of the authors who received each award<br>• Grant numbers awarded to each author<br>• The full name of each funder<br>• URL of each funder website<br>• Did the sponsors or funders play any role in the study design, data collection and analysis, decision to publish, or preparation of the manuscript?<br>• **NO** - Include this sentence at the end of your statement: *The funders had no role in study design, data collection and analysis, decision to publish, or preparation of the manuscript.*<br>• **YES** - Specify the role(s) played.<br><br>* typeset	
**Competing Interests**<br><br>Use the instructions below to enter a competing interest statement for this submission. On behalf of all authors, disclose any competing interests that could be perceived to bias this work—acknowledging all financial support and any other relevant financial or non-financial competing interests.<br><br>This statement is required for submission and **will appear in the published article** if the submission is accepted. Please make sure it is accurate and that any funding sources listed in your Funding Information later in the submission form are also declared in your Financial Disclosure statement.<br><br>View published research articles from *PLOS ONE* for specific examples.	The authors have declared that no competing interests exist.

Powered by Editorial Manager® and ProduXion Manager® from Aries Systems Corporation

**NO authors have competing interests**<br><br>Enter: *The authors have declared that no competing interests exist.*<br><br>**Authors with competing interests**<br><br>Enter competing interest details beginning with this statement:<br><br>*I have read the journal's policy and the authors of this manuscript have the following competing interests: [insert competing interests here]*<br><br>* typeset	
**Ethics Statement**<br><br>Enter an ethics statement for this submission. This statement is required if the study involved:<br><br>• Human participants<br>• Human specimens or tissue<br>• Vertebrate animals or cephalopods<br>• Vertebrate embryos or tissues<br>• Field research<br><br>Write "N/A" if the submission does not require an ethics statement.<br><br>General guidance is provided below. Consult the submission guidelines for detailed instructions. **Make sure that all information entered here is included in the Methods section of the manuscript.**	Field Research<br>This study involved the collection of plant and animal materials from the Mattheis Research Ranch, a research area owned by the University of Alberta, and the Eastern Irrigation District (EID) in Southern Alberta. Permission was granted by both and did not require a field permit number.

Powered by Editorial Manager® and ProduXion Manager® from Aries Systems Corporation

**Format for specific study types**<br><br>**Human Subject Research (involving human participants and/or tissue)**<br>• Give the name of the institutional review board or ethics committee that approved the study<br>• Include the approval number and/or a statement indicating approval of this research<br>• Indicate the form of consent obtained (written/oral) or the reason that consent was not obtained (e.g. the data were analyzed anonymously)<br><br>**Animal Research (involving vertebrate animals, embryos or tissues)**<br>• Provide the name of the Institutional Animal Care and Use Committee (IACUC) or other relevant ethics board that reviewed the study protocol, and indicate whether they approved this research or granted a formal waiver of ethical approval<br>• Include an approval number if one was obtained<br>• If the study involved *non-human primates*, add *additional details* about animal welfare and steps taken to ameliorate suffering<br>• If anesthesia, euthanasia, or any kind of animal sacrifice is part of the study, include briefly which substances and/or methods were applied<br><br>**Field Research**<br><br>Include the following details if this study involves the collection of plant, animal, or other materials from a natural setting:<br>• Field permit number<br>• Name of the institution or relevant body that granted permission	
**Data Availability**<br><br>Authors are required to make all data underlying the findings described fully available, without restriction, and from the time of publication. PLOS allows rare exceptions to address legal and ethical concerns. See the PLOS Data Policy and FAQ for detailed information.	Yes - all data are fully available without restriction

Powered by Editorial Manager® and ProduXion Manager® from Aries Systems Corporation

A Data Availability Statement describing where the data can be found is required at submission. Your answers to this question constitute the Data Availability Statement and **will be published in the article**, if accepted.<br><br>**Important:** Stating 'data available on request from the author' is not sufficient. If your data are only available upon request, select 'No' for the first question and explain your exceptional situation in the text box.<br><br>Do the authors confirm that all data underlying the findings described in their manuscript are fully available without restriction?	
**Describe where the data may be found in full sentences. If you are copying our sample text, replace any instances of XXX with the appropriate details.**<br><br>• If the data are **held or will be held in a public repository**, include URLs, accession numbers or DOIs. If this information will only be available after acceptance, indicate this by ticking the box below. For example: *All XXX files are available from the XXX database (accession number(s) XXX, XXX.*).<br>• If the data are all contained **within the manuscript and/or Supporting Information files**, enter the following: *All relevant data are within the manuscript and its Supporting Information files.*<br>• If neither of these applies but you are able to provide **details of access elsewhere**, with or without limitations, please do so. For example:<br><br>*Data cannot be shared publicly because of [XXX]. Data are available from the XXX Institutional Data Access / Ethics Committee (contact via XXX) for researchers who meet the criteria for access to confidential data.*<br><br>*The data underlying the results presented in the study are available from (include the name of the third party*	Data will be available after acceptance of the manuscript for publication.

and contact information or URL).<br>• This text is appropriate if the data are owned by a third party and authors do not have permission to share the data.<br><br>* typeset	
Additional data availability information:	Tick here if the URLs/accession numbers/DOIs will be available only after acceptance of the manuscript for publication so that we can ensure their inclusion before publication.

Powered by Editorial Manager® and ProduXion Manager® from Aries Systems Corporation

Manuscript

Click here to access/download;Manuscript;PLOSONE_manuscript_Worthy.do

Honey bees (*Apis mellifera*) modify plant-pollinator network structure, but do not alter wild species' interactions

Sydney H. Worthy[1,#a,*], John H. Acorn[1], Carol M. Frost[1]

[1] Department of Renewable Resources; University of Alberta, Edmonton, Alberta, Canada

[#a]Current address: City of Saskatoon, 1101 Avenue P North, Saskatoon, SK S7L 7K6

[*] **Corresponding author*****:*** worthy@ualberta.ca

Authors' Contributions

C.M.F., S.H.W., and J.H.A. conceptualized the project and designed the study. S.H.W. collected samples, and S.H.W. and J.H.A. identified the majority of the insect flower visitors. S.H.W. conducted data analysis with guidance from C.M.F. and J.H.A., and wrote the first draft of the manuscript. All authors contributed substantially to the final draft of the manuscript.

Abstract

Honey bees (*Apis mellifera*) are widely used for honey production and crop pollination, raising concern for wild pollinators, as honey bees may compete with wild pollinators for floral resources. The first sign of competition, before changes appear in wild pollinator abundance or diversity, may be changes to wild pollinator interactions with plants. Such changes for a community can be measured by looking at changes to metrics of resource use overlap in plant-pollinator interaction networks. Studies of honey bee effects on plant-pollinator networks have usually not distinguished whether honey bees alter wild pollinator interactions, or if they merely alter total network structure by adding their own interactions. To test this question, we experimentally introduced honey bees to a Canadian grassland and measured plant-pollinator interactions at varying distances from the introduced hives. We found that honey bees increased the network metrics of pollinator and plant functional complementarity and decreased interaction evenness. However, in networks constructed from just wild pollinator interactions, honey bee abundance did not affect any of the metrics calculated. Thus, all network structural changes to the full network (including honey bee interactions) were due only to honey bee-plant interactions, and not to honey bees causing changes in wild pollinator-plant interactions. Given widespread and increasing use of honey bees, it is important to establish whether they affect wild pollinator communities. Our results suggest that honey bees did not alter wild pollinator foraging patterns in this system, even in a year that was drier than the 20-year average.

Introduction

With the widespread use of managed honey bees (*Apis mellifera*) for crop pollination and honey production in parts of the world where they are not native, many studies have suggested that high honey bee densities negatively affect wild pollinators through interference or exploitative competition (reviewed in (1,2). Honey bees visit a wide variety of flower species (3) and high honey bee density has been linked to declines in wild pollinator diversity (4), abundance (5), floral resource use (6), and fitness (7), all via competition (8).

However, the first sign of competition between honey bees and wild pollinators may be changes to which flower species wild pollinators visit in the presence of honey bees, versus when honey bees are not present (5,6). If a wild pollinator is displaced by honey bees from one or more of its usual flower species (as reported for other competitive relationships between pollinators; e.g., (9,10), and for honey bees, (7)), the total set of flowers that the wild pollinator visits will either decrease (as in (11)), or will shift to include new flower species not previously visited by that pollinator species (12). Additionally, if honey bee use of a flower depletes nectar or pollen (13), the frequency of visits by wild pollinator species to that flower species may decrease, even if visitation does not cease altogether (14). The ecological effects of these changes may cascade across the plant-pollinator community, in that a wild pollinator species displaced by honey bees onto a new flower species may in turn displace the pollinators that had previously focused on that flower species (15). Furthermore, when honey bees dominate floral resources (7), this may also affect a plant’s ability to attract specific pollinators and ultimately affect plant fitness and persistence (16). Thus, shifts in resource overlap patterns in the plant-pollinator community may signal whether honey bees could cause eventual wild pollinator (or plant) diversity losses. To

examine this, an interaction network approach, considering interaction patterns across the whole community, can be useful (17).

Plant-pollinator interactions can be visualized as bipartite (two trophic-level) networks (Fig 1a), which comprise the set of interactions ("links") observed between species ("nodes"). These networks represent the pattern of interactions between all species within the plant-pollinator community that are included in the network model (18). Honey bees may change the structure and function of a plant-pollinator network in two different ways: (i) by adding new interactions (their own interactions with plants), thereby changing network structure without necessarily indicating competitive displacement (19); and/or by (ii) altering pre-existing plant-pollinator interactions, signaling competitive displacement. Changes to network structure caused by honey bees can be detected by comparing the values for network structural metrics under different honey bee densities (e.g., (17)). To detect changes to the entire network caused by honey bees (i), total networks including honey bee interactions must be examined (e.g., (20,21); Fig 1a.i-iii). To detect changes in just the set of wild species interactions (ii), networks not including honey bee interactions must be examined at high versus low honey bee densities (22,23; Fig 1a.iv). This is because network metrics are heavily influenced by the most abundant species (24) which honey bees often are (17). Therefore, changes to the pattern of wild species interactions cannot be detected when honey bee interactions are included in the network model. Here we assess both types of networks, while previous studies have typically tested only total networks (i) (17,25,26), but see (22,23,27). Importantly, excluding honey bees from a network model (ii) does not exclude honey bees' ecological effects, because the interactions being visualized in the network occurred when honey bees were present (ii). This is simply a method of

building a network model to clearly see wild pollinator interactions, as in (22,23,27) (Supplementary Information).

Fig 1. Visual representation of the plant-pollinator networks tested (a) and the hypothesized response of each network metric against honey bee abundance (b). a) Plant-pollinator interaction data from each of 19 sampling transects (left), four datasets were created (right): i) a full season all taxa dataset; ii) a mid-season all taxa dataset; iii) a full season bees-only dataset, where only bee interactions, including honey bee interactions, were kept from the full season network; and iv) a without-honey bees dataset, where only honey bee interactions were removed from the full season network. b) Predictions of the effects of increasing honey bee abundance on each network metric, for six metrics of resource overlap and six metrics associated with network stability. Yellow trend lines show predictions for metrics of total network structure, whereas blue trend lines show predictions for metrics calculated from just wild pollinator-plant interactions, excluding honey bee interactions. The rationale and references to support each prediction are in the Supplementary Information. Insect images are from (28).

We used six commonly employed metrics to quantify patterns of resource overlap in plant-pollinator communities (29), from both the pollinator perspective (how pollinator species overlap in what plant species they visit), and the plant perspective (how plant species overlap in what pollinator species visit them). The first metrics provide information on resource overlap from the pollinator perspective. The simplest is “generality”, which is the mean number of plant species visited by each pollinator species (29). We also calculated pollinator “niche overlap” (29), which is Horn’s information-theory-based index of overlap, ranging from 0 when all species’ interactions within a trophic level are distinct, to 1 when they are identical (30). Finally,

we calculated pollinator “functional complementarity”, a multivariate measure of the dissimilarity of resource use among pollinators. It calculates Euclidean distances between species based on the identity and frequency of their interactions, then clusters the distance matrix to produce a dendrogram, and calculates total branch length of the dendrogram, which provides a univariate expression of this multivariate difference in the set of plant species that each pollinator species interacts with (31). From the plant perspective, we calculated the equivalent three metrics: “vulnerability” (the mean number of pollinator species visiting each plant species; the name of this metric is from food webs and does not actually refer to vulnerability in plant-pollinator networks), plant “niche overlap” (the Horn’s information theoretic measure of overlap in pollinator species visiting each plant species), and plant “functional complementarity” (the index of multivariate difference in the set of pollinator species visiting each plant species).

We hypothesized that honey bees would compete with wild pollinators (8), excluding some wild pollinator species from some of their normal flower species (7), and thus narrowing their floral niches (5). We therefore predicted that in networks restricted to wild plant-pollinator interactions, we would see decreases in generality and pollinator niche overlap, and an increase in pollinator functional complementarity, with increasing honey bee abundance (Fig 1b). We also hypothesized that honey bees would dominate interactions with their preferred plant species (6), causing these plant species to be visited by fewer pollinator species (22). We thus predicted that in networks of just wild plant-pollinator interactions, we would see decreases in vulnerability and plant niche overlap and an increase in plant functional complementarity, with increasing honey bee abundance (Fig 1b).

We also calculated six other commonly reported network metrics to maximize comparability of our results with other studies reporting effects of honey bees on plant-pollinator

networks (17,22,23,26). These metrics were: nestedness, modularity, connectance, link density, interaction strength asymmetry, and interaction evenness. These additional metrics have been theoretically linked to network stability (32,33), but given that it is harder to interpret how changes in these metrics relate specifically to competition, we use them mostly as indicators of network structural change, and restrict our discussion of these metrics to the Supplementary Information. Our predictions for the effects of honey bees on the set of stability-related network metrics are shown in Fig 1b, and explained in detail in the Supplementary Information.

We expected to see the strongest effects of honey bees on native plant-pollinator interactions in midsummer (Fig 1a.ii) and therefore analyzed a midsummer dataset separately from the full season dataset. Honey bee colonies comprise between 10,000 to 50,000 individuals, attaining maximum size (34) and foraging distance (35) in midsummer.

We also examined how honey bee abundance affected network metrics for a bees-only version of the full season network (Fig 1a.iii). As most studies reporting effects of honey bees on wild pollinators have only assessed wild bees (2), we wondered if including all pollinating taxa (Hymenoptera, Diptera, Lepidoptera, Hemiptera, Coleoptera) might mask effects on just the wild bee-plant interactions.

Plant-pollinator interaction network studies are complicated by the effects of flower abundance and diversity, which vary spatially and temporally, and are not always controlled for. Network metrics also depend on sampling completeness (the proportion of the true interactions that were actually detected; (36), which is not always estimated, making comparability between studies low. Our study controls for effects of all of flower diversity, flower abundance, and sampling effort, and tests experimentally whether changes to plant-pollinator network structure are caused by honey bees changing wild plant-pollinator interactions. Our objectives were to: i)

determine if honey bees affect network metrics related to resource overlap and network stability; ii) determine if the effect of honey bees on network metrics is more detectable with higher honey bee colony size; and iii) determine if any effect of honey bees on plant-pollinator network metrics is due to changes in wild plant-pollinator interactions, or due to honey bee-plant interactions.

Materials and Methods

Experimental design

We arranged three clusters of honey bee hives, set at least 3 km apart, on the Mattheis Research Ranch, which is in mixedgrass prairie rangeland near the town of Brooks, Alberta, Canada (Fig 2; GPS locations in Table S1). Livestock have grazed these rangelands for a century, and this ranch has used fluctuating but light cattle stocking rates (approximately 0.7 animal unit months ha^{-1}) since 1970 (37). This region has average high and low temperatures of 18.3 °C in July and -12.9 °C in January, respectively, and 300 mm of rainfall annually. The hive clusters contained 48, 32, and 16 hives respectively, emulating the range of honey bee densities around commercial apiaries. We established eighteen 30 x 2 m transects at 100 m, 500 m, and 5000 m distances on either side of each cluster of honey bee hives for a total of six replicates (Fig 2).

Fig 2. The research location, Mattheis Ranch, in southern Alberta, Canada.

Sampling transects are shown as circles and honey bee hives as diamonds. Each hive location contained a cluster of hives (northernmost: 48 hives, central: 32 hives, southernmost: 16 hives).

The transect G5000 indicates the new location established when sampling was discontinued at F5000 after July 9th, 2019. Land outside the Mattheis Ranch is managed by the Eastern Irrigation District.

Between May 28 and August 28, 2019, observers visited each transect and surveyed insect flower visitation almost once per week (weather permitting), for a total of 10 collection rounds. The first transect sampled at the beginning of each day was chosen on a rotating schedule, and subsequent transects were sampled along a route of highest efficiency within that day. During some collection rounds, some transects could not be sampled due to cattle presence or a lack of flowers, resulting in different amounts of sampling for each transect (Table S1). Transects were visited only on warm (≥ 15°C), sunny days with winds under 50 km/h to improve the chance of pollinator activity (38,39). We measured wind speed with a Brunton Sherpa (Riverton, Wy, USA). Sampling occurred between 9:30 am and 5:00 pm, when insect flower visits are most frequent (40).

Transects were observed for 30 minutes by two people for a total of 60 person-minutes per transect per collection round (4200 total collection minutes). When large volumes of insects were captured, sampling was paused via a timer for processing and then resumed. All insects that visibly contacted the anthers or stigma of open flowers were collected with a hand net and placed in labelled individual vials, frozen, and identified to species in the lab. Some of these specimens (5%) were identified to morphospecies. When insects observed landing on flowers were missed they were recorded, but these data were excluded because the species-level identifications for these observations were heavily biased toward larger-bodied pollinators. All identifications were completed using a dissecting microscope, dichotomous keys, expert taxonomist help, and

comparison with reference specimens. Following flower visitor sampling, on each sampling round, all flowering plants on the transect were identified and their flowers were counted. A list of all references used for the insect and plant species identifications is included in Table S2. Occasionally, when flowers were not present on the marked transect, we moved the transect ≤ 10 m from its original location to reach flowers near the original transect demarcation. Moving the transect did not change the distance to the hives.

After July 9, 2019, sampling at one 5000 m transect "F5000" was halted after many honey bees were observed (presumably from a feral or unregistered hive, see Fig S1), since this replicate was intended to be a low-honey bee density transect. A new 5000 m transect, "G5000", was selected approximately 8000 m away from the northern 48-hive cluster (Fig 2). We treated these transects as distinct site replicates but controlled statistically for the lower number of collections at these by including number of collections as a covariate in our multiple regression models (see below and Table S1).

Network metric calculation

We pooled the observed insect-flower visitor interactions across the season for each transect, to construct one bipartite network for each transect, for a total of 19 networks (6 x 3 distances from hives, plus the new 5000 m transect "G5000" that was established mid-season).

We calculated the six network metrics described above to analyze honey bee effects on resource use overlap: generality, vulnerability, pollinator niche overlap, plant niche overlap, pollinator functional complementarity, and plant functional complemenarity (29). "Weighted" versions were used, meaning that interactions were weighted by their frequency, rather than just considering interaction presence or absence in calculating the metrics (41).

Additionally, we calculated the weighted versions of six network metrics to analyze the effects of honey bee abundance on network structural metrics related to stability: WNODF, a nestedness metric based on overlap and decreasing fill ("nestedness") (42), modularity, interaction strength asymmetry, interaction evenness, connectance, and link density. Calculation of these metrics and their interpretation is described in detail in the Supplementary Information. Each metric was calculated using the bipartite package (43). All statistical analyses were completed using R version 3.2.4. (44). Additionally, for comparability to (26), in a separate analysis, we pooled the network interactions across the season for the 100 m, 500 m, and 5000 m transects into meta-networks, and calculated each aforementioned network metric for each meta-network. We qualitatively compared them and present these results in the Supplementary Information.

Statistical Analysis

We used linear regression to test the effect of honey bee abundance on each of the six resource-overlap-related and the six stability-related network metrics. We assessed Pearson correlation coefficients between these metrics and found pollinator and plant functional complementarity were highly correlated, as well as link density and vulnerability (Table S3). We kept all metrics in the analysis, because these were ecological results rather than consequences of the metric definitions and might differ for different datasets. We pooled honey bee abundance (total number of honey bees observed visiting flowers) across the full season for each transect, and divided by number of collection rounds at that transect, to calculate "honey bee abundance" at a transect (Fig S1), which we used as the predictor variable in all full season statistical models. We used honey bee abundance rather than treatment (distance from honey bee hives) as the

predictor variable because distance from hives was not as closely associated with honey bee abundance as might have been expected (Fig S1), so honey bee abundance at a transect seemed like a better representation of honey bee "effect" at that transect, as in (23,25).

We tested the effect of honey bee abundance on each network metric for several datasets: i) each transect's network from the full season ("Full season all taxa"; Fig 1a.i); ii) each transect's network for only the mid-season sampling rounds, using honey bee abundance pooled across the mid-season divided by number of mid-season collection rounds at that transect as the predictor variable in this case ("Mid-season all taxa"; Fig 1a.ii described below); and iii) only bee interactions from the full season network ("Full season bees-only"; Fig 1a.iii). In these analyses each network included the observed honey bee interactions, as well as the wild pollinator interactions.

We first ran a simple linear regression (SLR) with honey bee abundance as the only predictor variable for each response variable (network metric) in each dataset. Some of the transects (replicates) were close together, so we plotted the standardized residuals of each SLR to visualize whether similar residuals were close together in space, and to examine whether a special correlation structure was necessary to account for any spatial autocorrelation (Fig S2).

We then ran generalized least squares (GLS) models with different correlation structures (no correlation, corEcp, corGaus, corSpher, corLin, corRatio) for each response variable (nlme package, (45)). After all models with correlation structures (including no special correlation structure) were run, the model with the lowest AICc value was selected (MuMin package, (46)). The best models for each predictor variable did not include special correlation structures, so simple linear regression models (SLR) were run. The exception was for modularity in the

without-honey bees network, so for this metric in this dataset, we ran a GLS model with the corRatio correlation structure.

We then ran an additional multiple regression model (MR) for each response variable in each dataset for which honey bee abundance was significant in the SLR. This was because flower abundance, number of available flower species, and collection effort were positively correlated with honey bee abundance (Table S4), so we included them as additional predictor variables in this second set of models, to test for an effect of honey bees above and beyond any correlation of honey bee abundance with these other variables (47). The predictor variables included in each full MR model were honey bee abundance, flower abundance, flower species richness, total number of collection rounds, the interaction between honey bee abundance and flower abundance, as well as the interaction between honey bee abundance and flower species richness. All continuous predictor variables were standardized by subtracting each transect's value from the mean and dividing by the standard deviation. Then, the full model and all possible simpler models were run, for a total of 21 regression models per response variable (nlme package, (45)), and the best model for each response variable was selected using AICc. In this second set of models, if honey bee abundance was retained after model selection and was significant, it would suggest that there was an effect of honey bees on that response variable that could not plausibly be attributed to other correlated predictor variables ((47); Supplementary Information). If honey bee abundance was significant in the SLR model, but was either not retained or not significant for the MR model, it would suggest that that response variable is related to honey bee abundance, but any effects of honey bees cannot be unambiguously attributed to honey bees. They may be due instead to flower abundance or diversity or collection effort, which honey bees were responding to themselves.

The assumptions of normality, homogeneity of variance, and linearity for each response variable were tested. If any assumptions were not met, that response variable was log transformed and model selection was repeated, after which, if assumptions were still not met, a Box-Cox transformation was applied (MASS package, (48)). Transformation did not improve normality or homogeneity of variances for the model for bees-only interaction strength asymmetry, so the results of this model were not interpreted as we could not find a robust modeling method.

To test whether the effects of honey bee abundance on the network metrics differed mid-season, we created a mid-season all taxa dataset (Fig 1a.ii). To determine which sampling dates the mid-season dataset should include, we divided the total honey bee abundance across the season, from all transects pooled, into three roughly equal-length periods: “early”, “mid” and “late”, based on natural breaks in the abundances of honey bees (Fig S3). We then tested the effects of honey bee abundance on all the same response variables as above, using the procedure outlined above, for this mid-season all taxa dataset. Because some transects’ networks were too species-poor in the mid-season to obtain accurate values (A100, B100, B500, E100, F5000), they were removed from this analysis.

Finally, we created a reduced network from the full season all taxa dataset where all honey bees were removed from the network, to look at the network metrics for wild pollinators only (Fig 1a.iv; (23). In this “without-honey bees” dataset we ran SLR and MR models that tested the effect of honey bee abundance and all predictor variables, respectively, on all the same response variables. If honey bee abundance was a significant predictor in these without-honey bees models, it would indicate that honey bees altered how wild pollinators interacted; if it was not, it would indicate that the changes to that network metric caused by honey bee abundance in

models for our other datasets were just due to the honey bee node and links contributing to the network structure.

This analysis involved interpreting 65 separate models, so to maintain Type I Error at 0.05, a Bonferroni-Holm correction was used in assessing significance of the final models (at P = 0.00077). However, all P-values are presented to allow a less conservative consideration of uncorrected P-values. To estimate the interaction sampling completeness, we divided the raw interaction richness across all transects by the Chao1 estimated total interaction richness across all transects (SpadeR, (49)). This calculated the proportion of the estimated total number of unique interactions that our sampling detected (as in (36)). We repeated this at the transect level for the full season all taxa, mid-season all taxa, and full season bees-only datasets.

Results

Diversity of pollinators, plants, and interactions in the grassland plant-pollinator community

A total of 281 pollinator species and 37 plant species were identified in 1,814 interactions, with 654 unique species pair interactions. Of these interactions, 425 were only observed once. The Chao1 estimated true total number of unique interactions was 1,779 interactions (95% confidence interval: 1500-2148 interactions), meaning that we observed 31 – 44% of the estimated total number of plant-pollinator interactions occurring in this study region (Fig S4). We also calculated the Chao1 estimated true number of unique interactions at each transect, for the full season, mid-season, and full season bees-only datasets (Table S5). At 100 m transects, honey bees accounted for 29.4% of the total interactions, at 500 m transects, 14.9% of

the total interactions, and at 5000 m transects, 1% of the total interactions. Bees (including honey bees) accounted for 872 (48.1%) of the total interactions. Of the remaining interactions, flies accounted for 494 (27.2%), butterflies accounted for 130 (7.2%), ants accounted for 104 (5.7%), beetles accounted for 100 (5.5%), wasps accounted for 62 (3.4%), true bugs accounted for 27 (1.5%), and day-flying moths accounted for 25 (1.4%). In the mid-season all taxa dataset, at 100 m transects, honey bees accounted for 49.6% of the interactions, at 500 m transects, 25.2% of the interactions, and at 5000 m transects, 2.4% of the interactions. All species identifications are listed in Tables S6 (pollinators) and S7 (plants).

Effect of honey bees on the structure of networks including honey bee interactions

In the full season all taxa dataset, in the SLR models (testing honey bee abundance as the only predictor against each network metric), increasing honey bee abundance was associated with significant changes in two network metrics related to resource overlap. Pollinator functional complementarity ($t = 5.92$, $P < 0.0001$) and plant functional complementarity ($t = 6.39$, $P < 0.0001$) significantly increased with honey bee abundance, indicating decreasing similarity in resource use between species within a trophic level with increasing honey bee abundance (Fig 3a, Table 1a). Increasing honey bee abundance was also associated with a significant decrease in interaction evenness ($t = -4.33$, $P = 0.0005$; Fig 3a, Table 1a). For the mid-season all taxa dataset (Fig 3a, Table 1b), and the full season bees-only dataset (Fig 3a, Table 1c), the results were very similar. All reported *P*-values are significant after the Bonferroni-Holm correction.

Table 1. Results of the simple linear regression (SLR) model with the lowest AICc values for each response variable, where the full model for each response variable contained honey bee abundance as the only predictor variable.

**Response variable**	**Confidence intervals (95%)**		**Adjusted R^2**	**Regression coefficient**	***t*-value**	***P*-value**
a) Full season all taxa						
log(Generality)	-0.06	0.11	-0.05	0.0238	0.58	0.5720
Vulnerability	-2.15	0.42	0.07	-0.8657	-1.44	0.1710
log(Pollinator niche overlap)	-0.31	0.03	0.13	-0.1398	-1.82	0.0910
Plant niche overlap	-0.02	0.01	-0.05	-0.0040	-0.55	0.5910
Pollinator functional complementarity	22.27	46.91	0.65	34.5940	5.92	**<0.0001**
Plant functional complementarity	21.72	43.12	0.69	32.4210	6.39	**<0.0001**
Weighted nestedness	-1.59	0.92	-0.05	-0.3371	-0.58	0.5732
Modularity	-0.05	0.05	-0.06	0.0011	0.05	0.9640
Weighted connectance	-0.04	-0.01	0.46	-0.0262	-4.07	0.0008
Link density	-1.08	0.37	0.00	-0.3514	-1.02	0.3203
Interaction strength asymmetry	-0.17	-0.01	0.18	-0.0879	-4.33	0.0381
Interaction evenness	-0.07	-0.02	0.50	-0.0475	-4.33	**0.0005**
b) Mid-season all taxa						
Generality	-0.12	0.12	-0.12	0.0023	0.04	0.9660
Vulnerability	-4.71	0.13	0.30	-2.2903	-2.18	0.0605
Pollinator niche overlap	-0.19	0.08	-0.02	-0.0596	-0.93	0.3786
Plant niche overlap	-0.21	0.09	-0.01	-0.0154	-0.48	0.6474
log(Pollinator functional complementarity)	0.33	0.96	0.57	0.6423	4.41	**0.0007**
Plant functional complementarity	25.05	38.66	0.88	31.8550	10.11	**<0.0001**
Weighted nestedness	-3.45	5.85	-0.08	1.2010	0.60	0.5679
Weighted connectance	-0.07	0.00	0.22	-0.0360	-2.22	0.0450
Link density	-1.61	-0.14	0.29	-0.8758	-2.58	0.0227
Interaction strength asymmetry	-0.21	0.04	0.09	-0.0880	-1.53	0.1497
Interaction evenness	-0.16	0.96	0.57	-0.1242	-7.94	**<0.0001**
c) Full season bees-only						
Generality	-0.09	0.21	-0.02	0.0617	0.88	0.3960
log(Vulnerability)	-0.20	0.01	0.15	-0.0927	-1.86	0.0851
Pollinator niche overlap	-0.10	0.03	0.04	-0.0375	-1.28	0.2240
Plant niche overlap	-0.06	0.05	-0.08	-0.0031	-0.12	0.9058
log(Pollinator functional complementarity)	0.34	0.96	0.51	0.6516	4.42	**0.0004**
Plant functional complementarity	24.96	44.42	0.76	34.6900	7.52	**<0.0001**
Weighted nestedness	-2.78	2.38	-0.07	-0.1993	-0.17	0.8700
Weighted connectance	-0.06	-0.01	0.32	-0.0361	-3.05	0.0073
Link density	-0.36	0.15	-0.01	-0.1088	-0.90	0.3820

Interaction strength asymmetry[a]	-0.23	0.06	0.03	-0.0376	-1.24	0.2333
Interaction evenness	-0.10	-0.05	0.68	-0.0777	-6.33	**<0.0001**
d) Full season without-honey bees						
log(Generality)	-0.04	0.14	0.02	0.0482	1.15	0.2660
Vulnerability	-1.12	1.66	-0.05	0.2684	0.41	0.6890
log(Pollinator niche overlap)	-0.35	-0.02	0.21	-0.1877	-2.40	0.0279
Plant niche overlap	-0.02	0.01	0.00	-0.0069	-0.95	0.3530
Pollinator functional complementarity	-12.18	14.07	-0.06	0.9445	0.15	0.8810
Plant functional complementarity	-9.67	13.14	-0.05	1.7320	0.32	0.7530
Weighted nestedness	-1.58	0.24	0.09	-0.6727	-1.58	0.1350
Modularity (*CorRatio*)[b]	0.00	0.02	NA	0.0081	1.25	0.2282
Weighted connectance	-0.03	0.00	0.20	-0.0162	-2.34	0.0320
Link density	-0.55	0.88	-0.04	0.1616	0.48	0.6390
Interaction strength asymmetry	-0.16	0.03	0.07	-0.0669	-1.52	0.1470
Interaction evenness	-0.02	0.02	-0.06	-0.0009	-0.09	0.9310

Results are shown for the three datasets: a) the full season all taxa dataset, b) the mid-season all taxa dataset, and c) the full season bees-only dataset, all of which included the honey bee interactions, and d) the full season without-honey bees dataset. The regression coefficients (slopes) are a measure of the expected change in the response variable associated with a one-unit change in the predictor. Bolded P-values denote significance with Bonferroni-Holm correction ($P < 0.00077$). Transformations are listed with the response variable.

[a] Assumptions of normality and homogeneity of variance were not met for this statistical test, so P-value is not likely accurate and is not interpreted.

[b] Adjusted R^2 cannot be calculated for a model fit using generalized least squares.

Fig 3. Predictions vs. results of network metric analyses from with-honey bees networks (a) and without-honey bees networks (b).

a) Relationships between honey bee abundance and the network metrics: (i) pollinator functional complementarity, (ii) plant functional complementarity, and (iii) interaction evenness that were significantly related to honey bee abundance across three datasets (full season all taxa, mid-season all taxa, and full season bees-only). Solid regression lines indicate significant relationships with Bonferroni-Holm correction, whereas dotted lines indicate insignificant relationships. b) Relationships between honey bee abundance and the network metrics: (i) pollinator niche overlap, (ii) pollinator functional complementarity, (iii) plant functional complementarity, (iv) weighted connectance, and (v) interaction evenness in the without-honey bees dataset. Dashed regression lines indicate non-significant relationships with Bonferroni-Holm correction. Although (i) and (iv) appear to contain trends, these cannot be unambiguously attributed to honey bee abundance, as they can be explained by flower community variables and collection effort (Table 2d) that were somewhat correlated with honey bee abundance (Table S4).

In the second set of MR models (including all predictor variables), flower species richness and total number of collections best explained most response variables (Table 2a). However, increasing honey bee abundance was still associated with significant increases in pollinator functional complementarity ($t = 5.51$, $P < 0.0001$) and plant functional complementarity ($t = 5.75$, $P < 0.0001$), and a significant decrease in interaction evenness ($t = -4.33$, $P = 0.0005$) (Table 2a), indicating that these effects on plant-pollinator network structure could be attributed to honey bees, rather than to flower community variables that were correlated with honey bee abundance. For the mid-season all taxa dataset (Table 2b) and the full season

bees-only dataset (Table 2c), the results were very similar, except that in the full season bees-only dataset, pollinator functional complementarity was not significantly increased by honey bees after the Bonferroni-Holm correction, even though it still had a very low P-value (t = 3.57, P = 0.0026).

Table 2. Results of the multiple regression (MR) model with the lowest AICc value for each response variable.

**Response variable**	**Predictor variables retained in final model**	**Confidence intervals (95%)**		**Adjusted R^2**	**Partial regression coefficient**	***t*-value**	***P*-value**
a) Full season all taxa							
Pollinator functional complementarity	honey bee<br>collection	16.13<br>8.79	36.34<br>29.00	 0.81	26.2380<br>18.8970	5.51<br>3.97	**<0.0001**<br>0.0011
Plant functional complementarity	honey bee<br>collection	16.33<br>5.30	35.39<br>24.36	 0.80	25.8630,<br>14.8320	5.75<br>3.30	**<0.0001**<br>0.0045
Weighted connectance	flower species richness<br>collection	-0.03<br>-0.03	-0.01<br>0.00	0.68 	-0.0210<br>-0.0146	-3.50<br>-2.43	0.0030<br>0.0271
Interaction strength asymmetry	flower species richness	-0.19	-0.04	0.33	-0.1136	-3.25	0.0047
Interaction evenness	honey bee	-0.07	-0.02	0.50	-0.0475	-4.33	**0.0005**
b) Mid-season all taxa							
log(Pollinator functional complementarity)	honey bee<br>flower species richness	0.51<br>0.17	0.97<br>0.62	 0.79	0.7395<br>0.3952	6.98<br>3.76	**<0.0001**<br>0.0027
Plant functional complementarity	honey bee<br>flower species richness	28.50<br>2.74	39.16<br>13.32	 0.93	33.8290<br>8.0290	13.84<br>3.31	**<0.0001**<br>0.0062
Weighted connectance	honey bee<br>flower species richness	-0.07<br>-0.07	-0.02<br>-0.01	 0.55	-0.0460<br>-0.0409	-3.62<br>-3.23	0.0035<br>0.0072
Link density	honey bee	-1.61	-0.14	0.29	-0.8758	-2.58	0.0227
Interaction evenness	honey bee	-0.16	-0.09	0.82	-0.1242	-7.94	**<0.0001**
c) Full season bees-only							
log(Pollinator functional complementarity)	honey bee<br>collection	0.18<br>0.18	0.72<br>0.72	 0.71	0.4534<br>0.4481	3.57<br>3.52	0.0026<br>0.0028
Plant functional complementarity	honey bee<br>collection	20.16<br>2.03	39.07<br>20.93	 0.82	29.6150<br>11.4760	6.64<br>2.57	**<0.0001**<br>0.0204
Weighted connectance	flower species richness	-0.07	-0.03	0.58	-0.0476	-5.20	**0.0001**
Interaction evenness	honey bee	-0.10	-0.05	0.68	-0.0887	-4.56	**0.0003**
d) Full season without-honey bees							
log(Pollinator niche overlap)	flower species richness	-0.11	-0.03	0.39	-0.0704	-3.54	0.0025
Pollinator functional complementarity	collection	6.85	26.72	0.39	16.7810	3.56	0.0024
Plant functional complementarity	collection	5.33	23.02	0.37	14.1780	3.28	0.0035

Weighted connectance	flower species richness<br>collection	-0.03<br>-0.03	-0.00<br>-0.00	0.61	-0.0156<br>-0.0144	-2.65<br>-2.46	0.0174<br>0.0256
Interaction evenness	flower species richness	-0.01	0.03	0.00	-0.0100	0.97	0.3450

The full MR model for each response variable contained honey bee abundance, flower abundance, flower species richness, the interactions between honey bee abundance and flower abundance, and between honey bee abundance and flower species richness, and collection effort as predictor variables. Only response variables for which the P-value in the corresponding simple linear regression (SLR) model was ≤ 0.00077 are shown here, with the exception of d), where they were also included if a) was ≤ 0.05. Results are shown for four datasets: a) the full season all taxa dataset, b) the mid-season all taxa dataset, c) the full season bees-only dataset, all of which included the honey bee interactions, and d) the full season without-honey bees dataset. The partial regression coefficients are the expected change in the response variable associated with a one unit change in a predictor variable holding the other predictor variables constant. Bolded P-values denote significance with Bonferroni-Holm correction ($P < 0.00077$). Transformations are listed with the response variable.

Effect of honey bees on the without-honey bees network structure

In contrast to all of the above results, the full season without-honey bees dataset with only honey bee abundance as a predictor variable (SLR models) showed that honey bees did not significantly affect pollinator functional complementarity, plant complementarity, or interaction evenness, or any other network metric (Fig 3b, Table 2d). Although some P-values were low in the SLR models (Table 1d), in the MR models, honey bee abundance was removed during model selection in every case (Table 2d), suggesting that the low P-values in the SLR models could be explained by correlated effects of the flower variables or collection effort better than they could

be explained by honey bee abundance. This disappearance of any significant effect of honey bee abundance on any network metrics when honey bees were not considered as part of the network was not an artifact of having decreased the network size by removing the honey bee interactions (see comparison of all network sizes in Table S8).

Discussion

As predicted, total plant-pollinator network structure (including honey bee interactions), changed with increasing honey bee abundance, as reported in other studies (17,23,25,26). Across three datasets (full season all taxa, mid-season all taxa, and full season bees-only), the changes due to honey bees (rather than potentially due to flower community variables correlated with honey bee abundance) were that pollinator functional complementarity and plant functional complementarity significantly increased, and interaction evenness significantly decreased as honey bee abundance increased. The effect sizes were highest in the mid-season all taxa dataset, when honey bee abundances were highest (compare regression coefficients in Table 2a to partial regression coefficients in Table 2b). The exception was that pollinator functional complementarity did not change significantly in the bees-only dataset (Table 2c). When looking at the meta-networks (Fig S5), which had interactions pooled across transects by distance from hives, similar to (26), rather than pooled by transect, the results were qualitatively similar (Table S9), suggesting that our results are robust across different analysis methods.

However, when considering only wild plant-pollinator interactions in the without-honey bees dataset, honey bee abundance (which varied from high to almost zero between these sites) did not affect any of the network metrics (Table 2d), suggesting that all changes to network structure associated with honey bee abundance in this study were caused by the honey bees'

interactions contributing to the network structure, rather than by honey bees altering the interactions of wild pollinators and plants. Thus, adding honey bees had no detectable effect on the network structure of the wild plant-pollinator interactions in this region, whether considering metrics of resource overlap, or metrics associated with network stability.

Magrach et al, (17), who reported changes in plant-pollinator network structure between orange groves in Spain in which honey bees accounted for 72% vs. 38% of interactions, found similar results to our with-honey bee networks: honey bees decreased interaction evenness and increased functional complementarity in their system. The increase in functional complementarity indicates increasing dissimilarity in the interaction composition of pollinators and plants in the network, which can be thought of as a reduction of their resource use overlap (31). Although this metric accounts for the interactions of all species in the community, its value can change in response to the addition of an abundant generalist species' interactions, such as the honey bee, because an abundant generalist species' interaction identities and frequencies are so different from those of less abundant and generalist species (corresponding to a long dendrogram branch length). However, (17) did not test with- versus without-honey bee networks for honey bee abundance effects on these metrics. Although their with-honey bee network results are similar to ours, we found that in our without-honey bees network, the effect of honey bee abundance disappeared, suggesting no effect of honey bees on wild plant-pollinator interactions.

Similarly, Valido et al (26), who tested changes in network structure before vs. after honey bees were added to one site in Tenerife, Canary Islands in three consecutive years, did not remove the honey bee node from the networks and re-test effects of honey bee abundance on just the wild pollinator interactions. Additionally, they did not control for floral abundance or flower species richness as potentially correlated predictor variables. Furthermore, we had similar

sampling effort, range of honey bee abundances tested, and estimated sampling completeness to both (17) and (26). Taken together, this suggests that if more potentially correlated variables had been controlled for, these two studies may also have found a non-significant effect of honey bees on wild plant-pollinator network structure. Alternatively, it may be that honey bees shift wild pollinator interactions in other systems more than in the grassland system where our study took place. Lázaro et al (23), similar to our study, tested honey bee effects on total network structure, as well as on the network structure of just wild pollinator interactions, and found larger effects on total network structure. They reported that the effects of honey bee abundance on wild bee network metrics that they observed were caused by changes in wild bee species richness, whereas in our study, wild pollinator species richness did not change with increasing honey bee abundance (Worthy et al in review).

The most unexpected result from our study was that we found no effect of honey bees on nestedness, whether honey bee interactions were included or not (Table 1a-d). This contrasts with several studies that have reported increases in network nestedness when honey bees were present (19–21,23,50). However, all these studies except (23) tested honey bee effects on "binary" nestedness, calculated without considering interaction frequencies, whereas we used "weighted" nestedness, which incorporates interaction frequency, because (51) showed that changes to weighted nestedness are a better indicator of shifts in resource partitioning. Our finding of no change to weighted nestedness associated with increasing honey bee abundance is further evidence that honey bees did not change patterns of resource partitioning by wild pollinators or plants (51). Our findings for the other stability-related metrics for the with-honey bees networks are similar to the findings of other studies (see Supplementary Information).

Our failure to find evidence that honey bees shift resource use by wild pollinators, or shift visitation of plant species, may indicate that honey bees do not compete with wild pollinators in this system, due to abundant floral resources that are not limiting. Additionally, honey bees interacted frequently with non-native plants. Alberta's grasslands are diverse and dominated by native plant species, but several exotic plants exist in the region; sweet clover (*Melilotus* spp.), sow-thistle (*Sonchus* spp.), and cicer milk-vetch (*Astragalus cicer*), in particular, exist in scattered patches throughout the study region. We observed that these exotic species were highly attractive to pollinators of all types. In their network study, (52) determined that honey bees preferred exotic plants over native plants. We also observed that small-flowered and small-stemmed plants were less appealing to larger-bodied pollinators. This preference may have prevented honey bees from outcompeting wild pollinators for resources, preserving native plant-pollinator interactions.

The plant-pollinator network in this system appears to accommodate honey bee interactions, integrating them into the system without major changes, despite honey bees taking a central role (Fig S5). Honey bees may spread pathogens and parasites to some wild bee species via shared flowers (53), or cause other changes to wild plant or pollinator fitness undetectable from looking at changes to flower visitation (16). Furthermore, we did not detect all occurring plant-pollinator interactions at our transects. Our estimated sampling completeness was 37% overall (see Table S4), suggesting that we missed many rare interactions. Rare interactions typically involve rare species, which may be particularly sensitive to competition from honey bees (3). However, understanding honey bee effects on the subset of detected interactions is useful because the most detectable interactions are the most frequent, and are those that contribute most to ecosystem function (54).

Additionally, our study was conducted in only one season, and plant-pollinator interactions are known to be highly variable year to year (55). This variability is largely because of species and interaction turnover, however, and network structure is expected to be more consistent between years (55), so our conclusions about network structure might be expected to hold in other years. The sampled year 2019 was drier than the 20-year average (Table S9), meaning floral resources should have been more limiting than in a normal year (56), and therefore we should have been more likely to see competitive effects of honey bees on native pollinators in 2019. We also sampled in 2018, another very dry year (Table S10), and though the data were not of high enough quality to publish, they indicated the same results.

Conclusions

Honey bees have been frequently associated with wild pollinator decline, and because regulating honey bee densities might assist wild pollinator conservation, it is worth establishing whether honey bees are a significant threat to wild pollinators. Our study took place in a part of Canada with high bee diversity, in a dry year when floral resources for pollinators should have been more limiting than usual. In this setting we found no effects of honey bees on wild pollinator-plant network metrics, suggesting that even at high densities, honey bees do not detectably cause shifts in wild pollinator foraging habits in this region. Future studies should control for the effects of flower abundance and diversity on plant-pollinator network structure, and distinguish the effects of honey bee interactions on total network structure from effects of honey bees on just the wild pollinator interactions with plants.

Acknowledgements

We thank Brittany Wingert, Irene Jimenez Roncancio, James Glasier, Greg Pohl, Lincoln Best, and the University of Calgary for their assistance in the identification of specific insect taxa. We also thank assistants Alexandra Johnson and Janelle Goodine, and volunteers Connor Nelson, Olivia DeBourcier, Zachary Roote, Ferf Brownoff, Sara Peterson, Olivia Hrehoruk, Rykkar Jackson, and Victoria Dubord for all their support on this project. We thank Edwin and Ruth Mattheis for donating the Mattheis Research Ranch to the University of Alberta, and Marcel Busz and Lisa Raatz for assistance with logistics at the ranch. We also thank the Eastern Irrigation District for allowing the use of their land. Honey bees were generously supplied by Pankratz Beekeeping. We thank Cameron Carlyle and Mark Poesch for helpful discussion. Funding was provided by the University of Alberta's Rangeland Research Institute and the Alberta Conservation Association.

References

1. Iwasaki JM, Hogendoorn K. Mounting evidence that managed and introduced bees have negative impacts on wild bees: an updated review. Current Research in Insect Science. 2022;2:100043.

2. Mallinger RE, Gaines-Day HR, Gratton C. Do managed bees have negative effects on wild bees?: A systematic review of the literature. Raine NE, editor. PLoS ONE. 2017 Dec 8;12(12):e0189268.

3. Waser NM, Ollerton J, editors. Plant-pollinator interactions: from specialization to generalization. Chicago: University of Chicago Press; 2006. 445 p.

4. Badano EI, Vergara CH. Potential negative effects of exotic honey bees on the diversity of native pollinators and yield of highland coffee plantations. Agricultural and Forest Entomology. 2011 Nov;13(4):365–72.

5. Forup ML, Memmott J. The relationship between the abundances of bumblebees and honeybees in a native habitat. Ecol Entomol. 2005 Feb;30(1):47–57.

6. Nielsen A, Reitan T, Rinvoll AW, Brysting AK. Effects of competition and climate on a crop pollinator community. Agriculture, Ecosystems & Environment. 2017 Aug;246:253–60.

7. Hudewenz A, Klein A. Red mason bees cannot compete with honey bees for floral resources in a cage experiment. Ecol Evol. 2015 Nov;5(21):5049–56.

8. Cane JH, Tepedino VJ. Gauging the Effect of Honey Bee Pollen Collection on Native Bee Communities. Conservation Letters. 2017;10(2):205–10.

9. Morse DH. Resource Partitioning in Bumble Bees: The Role of Behavioral Factors. Science. 1977 Aug 12;197(4304):678–80.

10. Taggar AK, McGrath E, Despland E. Competition between a native and introduced pollinator in unmanaged urban meadows. Biol Invasions. 2021 Jun;23(6):1697–705.

11. Heinrich B. Resource Partitioning Among Some Eusocial Insects: Bumblebees. Ecology. 1976 Aug;57(5):874–89.

12. Fontaine C, Collin CL, Dajoz I. Generalist foraging of pollinators: diet expansion at high density: Flexibility of pollinator generalization. Journal of Ecology. 2008 Sep;96(5):1002–10.

13. Wignall VR, Brolly M, Uthoff C, Norton KE, Chipperfield HM, Balfour NJ, et al. Exploitative competition and displacement mediated by eusocial bees: experimental evidence in a wild pollinator community. Behav Ecol Sociobiol. 2020 Dec;74(12):152.

14. Cervantes- Loreto A, Ayers CA, Dobbs EK, Brosi BJ, Stouffer DB. The context dependency of pollinator interference: How environmental conditions and co- foraging species impact floral visitation. Irwin R, editor. Ecology Letters. 2021 Jul;24(7):1443–54.

15. Montero- Castaño A, Vilà M. Influence of the honeybee and trait similarity on the effect of a non- native plant on pollination and network rewiring. Ayasse M, editor. Funct Ecol. 2017 Jan;31(1):142–52.

16. Aizen MA, Morales CL, Vázquez DP, Garibaldi LA, Sáez A, Harder LD. When mutualism goes bad: density-dependent impacts of introduced bees on plant reproduction. New Phytol. 2014 Oct;204(2):322–8.

17. Magrach A, González-Varo JP, Boiffier M, Vilà M, Bartomeus I. Honeybee spillover reshuffles pollinator diets and affects plant reproductive success. Nat Ecol Evol. 2017 Sep;1(9):1299–307.

18. Delmas E, Besson M, Brice MH, Burkle LA, Dalla Riva GV, Fortin MJ, et al. Analysing ecological networks of species interactions. Biological Reviews. 2019;94(1):16–36.

19. Geslin B, Gauzens B, Baude M, Dajoz I, Fontaine C, Henry M, et al. Massively Introduced Managed Species and Their Consequences for Plant–Pollinator Interactions. Advances in Ecological Research. 2017;57:147–99.

20. Santos, GM, Aguiar CML, Genini J, Martins CF, Zanella FCV, Mello MAR. Invasive Africanized honeybees change the structure of native pollination networks in Brazil. Biol Invasions. 2012 Nov;14(11):2369–78.

21. Giannini TC, Cordeiro GD, Freitas BM, Saraiva AM, Imperatriz-Fonseca VL. The Dependence of Crops for Pollinators and the Economic Value of Pollination in Brazil. Journal of Economic Entomology. 2015 Jun 1;108(3):849–57.

22. Ropars L, Dajoz I, Fontaine C, Muratet A, Geslin B. Wild pollinator activity negatively related to honey bee colony densities in urban context. Blenau W, editor. PLoS ONE. 2019 Sep 12;14(9):e0222316.

23. Lázaro A, Müller A, Ebmer AW, Dathe HH, Scheuchl E, Schwarz M, et al. Impacts of beekeeping on wild bee diversity and pollination networks in the Aegean Archipelago. Ecography. 2021 Sep;44(9):1353–65.

24. Vázquez DP, Melián CJ, Williams NM, Blüthgen N, Krasnov BR, Poulin R. Species abundance and asymmetric interaction strength in ecological networks. Oikos. 2007 Jul;116(7):1120–7.

25. Prendergast KS, Ollerton J. Spatial and temporal scale of analysis alter conclusions about the effects of urbanisation on plant–pollinator networks. Arthropod-Plant Interactions. 2022 Dec;16(6):553–65.

26. Valido A, Rodríguez-Rodríguez MC, Jordano P. Honeybees disrupt the structure and functionality of plant-pollinator networks. Sci Rep. 2019 Dec;9(1):4711.

27. Ropars L, Affre L, Thébault É, Geslin B. Seasonal dynamics of competition between honey bees and wild bees in a protected Mediterranean scrubland. Oikos [Internet]. 2022 Apr [cited 2023 Feb 3];2022(4). Available from: https://onlinelibrary.wiley.com/doi/10.1111/oik.08915

28. SVG SILH. SVG SILH [Internet]. SVG SILH. 2022. Available from: https://svgsilh.com/

29. Dormann CF, Gruber B, Frueund J. Introducing the bipartite Package: Analysing Ecological Networks. R News. 2008;8(2):8–11.

30. Horn HS. Measurement of “Overlap” in Comparative Ecological Studies. The American Naturalist. 1966 Sep;100(914):419–24.

31. Devoto M, Bailey S, Craze P, Memmott J. Understanding and planning ecological restoration of plant-pollinator networks. Ecology Letters. 2012;15(4):319–28.

32. Okuyama T, Holland JN. Network structural properties mediate the stability of mutualistic communities. Ecol Letters. 2008 Mar;11(3):208–16.

33. Thébault E, Fontaine C. Stability of Ecological Communities and the Architecture of Mutualistic and Trophic Networks. Science. 2010 Aug 13;329(5993):853–6.

34. Canadian Honey Council. Bee Facts [Internet]. Canadian Honey Council. 2018 [cited 2021 Jun 18]. Available from: https://honeycouncil.ca/bee-facts/

35. Couvillon MJ, Schürch R, Ratnieks FLW. Waggle Dance Distances as Integrative Indicators of Seasonal Foraging Challenges. Smagghe G, editor. PLoS ONE. 2014 Apr 2;9(4):e93495.

36. Chacoff NP, Vázquez DP, Lomáscolo SB, Stevani EL, Dorado J, Padrón B. Evaluating sampling completeness in a desert plant-pollinator network: Sampling a plant-pollinator network. Journal of Animal Ecology. 2012 Jan;81(1):190–200.

37. Thomas BW, Gao X, Zhao M, Bork EW, Hao X. Grazing altered carbon exchange in a dry mixedgrass prairie as a function of soil texture. Can J Soil Sci. 2017 Dec 18;CJSS-2017-0052.

38. Pasek JE. Influence of wind and windbreaks on local dispersal of insects. Agriculture, Ecosystems & Environment. 1988;22–23:539–54.

39. Sanderson RA, Goffe LA, Leifert C. Time-series models to quantify short-term effects of meteorological conditions on bumblebee forager activity in agricultural landscapes. Agricultural and Forest Entomology. 2015;17(3):270–6.

40. Lefebvre V, Fontaine C, Villemant C, Daugeron C. Are empidine dance flies major flower visitors in alpine environments? A case study in the Alps, France. Biology Letters. 2014;10(11).

41. Bersier LF, Banašek-Richter C, Cattin MF. QUANTITATIVE DESCRIPTORS OF FOOD-WEB MATRICES. Ecology. 2002 Sep;83(9):2394–407.

42. Almeida-Neto M, Guimarães P, Guimarães PR, Loyola RD, Ulrich W. A consistent metric for nestedness analysis in ecological systems: reconciling concept and measurement. Oikos. 2008 Aug;117(8):1227–39.

43. Dormann CF, Frueund J, Bluethgen N, Gruber B. Indices, graphs and null models: analyzing bipartite ecological networks. The Open Ecology Journal. 2009;2:7–24.

44. R Core Team. R: A language and environment for statistical computing. [Internet]. R Foundation for Statistical Computing; 2020. Available from: https://www.r-project.org/

45. Jose P, Douglas B, Saikat D, Sarkar D, R Core Team. nlme: Linear and Nonlinear Mixed Effects Models. 2020.

46. Bartoń K. MuMIn: Multi-model inference. [Internet]. Available from: https://r-forge.rproject.org/R/?group_id=346

47. Morrissey MB, Ruxton GD. Multiple Regression Is Not Multiple Regressions: The Meaning of Multiple Regression and the Non-Problem of Collinearity. Philosophy, Theory, and Practice in Biology [Internet]. 2018 Jun [cited 2022 Apr 28];10(20220112). Available from: http://hdl.handle.net/2027/spo.16039257.0010.003

48. Venables WN, Ripley BD. Modern Applied Statistics with S [Internet]. 4th ed. Springer; 2002. Available from: http://www.stats.ox.ac.uk/pub/MASS4/

49. Chao A, Ma KH, Hsieh TC, Chiu CH. SpadeR: Species-Richness Prediction and Diversity Estimation with R [Internet]. 2016. Available from: https://cran.r-project.org/package=SpadeR

50. Traveset A, Heleno R, Chamorro S, Vargas P, McMullen CK, Castro-Urgal R, et al. Invaders of pollination networks in the Galápagos Islands: emergence of novel communities. Proc R Soc B. 2013 May 7;280(1758):20123040.

51. Staniczenko PPA, Kopp JC, Allesina S. The ghost of nestedness in ecological networks. Nat Commun. 2013 Jun;4(1):1391.

52. Bendel CR, Kral- O'Brien KC, Hovick TJ, Limb RF, Harmon JP. Plant–pollinator networks in grassland working landscapes reveal seasonal shifts in network structure and composition. Ecosphere [Internet]. 2019 Jan [cited 2021 Aug 2];10(1). Available from: https://onlinelibrary.wiley.com/doi/10.1002/ecs2.2569

53. Graystock P, Goulson D, Hughes WOH. Parasites in bloom: flowers aid dispersal and transmission of pollinator parasites within and between bee species. Proc R Soc B. 2015 Aug 22;282(1813):20151371.

54. Winfree R, W. Fox J, Williams NM, Reilly JR, Cariveau DP. Abundance of common species, not species richness, drives delivery of a real-world ecosystem service. Shea K, editor. Ecol Lett. 2015 Jul;18(7):626–35.

55. Zografou K, Swartz MT, Tilden VP, McKinney EN, Eckenrode JA, Sewall BJ. Stable generalist species anchor a dynamic pollination network. Ecosphere [Internet]. 2020 Aug [cited 2022 Apr 30];11(8). Available from: https://onlinelibrary.wiley.com/doi/10.1002/ecs2.3225

56. Phillips BB, Shaw RF, Holland MJ, Fry EL, Bardgett RD, Bullock JM, et al. Drought reduces floral resources for pollinators. Glob Change Biol. 2018 Jul;24(7):3226–35.

57. Bascompte J, Jordano P, Melian CJ, Olesen JM. The nested assembly of plant-animal mutualistic networks. Proceedings of the National Academy of Sciences. 2003 Aug 5;100(16):9383–7.

58. Bastolla U, Fortuna MA, Pascual-García A, Ferrera A, Luque B, Bascompte J. The architecture of mutualistic networks minimizes competition and increases biodiversity. Nature. 2009;458(7241):1018–20.

59. Aizen MA, Morales CL, Morales JM. Invasive Mutualists Erode Native Pollination Webs. Simberloff D, editor. PLoS Biol. 2008 Feb 12;6(2):e31.

60. Frost CM, Allen WJ, Courchamp F, Jeschke JM, Saul WC, Wardle DA. Using Network Theory to Understand and Predict Biological Invasions. Trends in Ecology and Evolution. 2019;34(9):831–43.

61. Almeida-Neto M, Ulrich W. A straightforward computational approach for measuring nestedness using quantitative matrices. Environmental Modelling and Software. 2011;26(2):173–8.

62. Newman MEJ, Girvan M. Finding and evaluating community structure in networks. Phys Rev E. 2004 Feb 26;69(2):026113.

63. Bascompte J, Jordano P, Olesen JM. Asymmetric coevolutionary networks facilitate biodiversity maintenance. Science. 2006;312(5772):431–3.

64. Blüthgen N. Why network analysis is often disconnected from community ecology: A critique and an ecologist’s guide. Basic and Applied Ecology. 2010 May;11(3):185–95.

65. Tylianakis JM, Tscharntke T, Lewis OT. Habitat modification alters the structure of tropical host-parasitoid food webs. Nature. 2007;445(7124):202–5.

66. Acorn J. Butterflies of Alberta. Edmonton: Lone Pine Publishing; 1993.

67. Vandyk J, editor. BugGuide.Net: Identification, Images, & Information For Insects, Spiders & Their Kin For the United States & Canada. [Internet]. Iowa State University; 2021. Available from: https://bugguide.net/

68. Warren AD, Davis KJ, Strangeland EM, Pelham JP, Willmott KR, Grishin NV. Butterflies of America [Internet]. Illustrated Lists of American Butterflies (North and South Amerca). 2016. Available from: https://www.butterfliesofamerica.com/

69. Glasier JRN, Acorn, John H., Nielsen SE, Proctor H. Ants (Hymenoptera: Formicidae) of Alberta: A key to species based primarily on the worker caste. CJAI. 2013 Jul 4;22.

70. Acorn J. The Tiger Beetles of Alberta: Killers on the Clay, Stalkers on the Sand. Edmonton: University of Alberta Press; 2001.

71. Acorn J. Ladybugs of Alberta: finding the spots and connecting the dots. 1st ed., 1st print., 2007. Edmonton: University of Alberta Press; 2007. 169 p. (Alberta insects series).

72. Arnett RH, Thomas MC, editors. American beetles, Volume I: Archostemata, Myxophaga, Adephaga, Polyphaga: Staphyliniformia. Boca Raton, Fla: CRC Press; 2000. 2 p.

73. Arnett RH, Thomas MC, Skelley PE, Frank JH, editors. American beetles, Volume II: Polyphaga: Scarabaeoidea through Curculionoidea. Boca Raton, Fla: CRC Press; 2002. 2 p.

74. Bousquet Y, Bouchard P, Davies A, Sikes D. Checklist of beetles (Coleoptera) of Canada and Alaska. Second edition. ZK. 2013 Dec 6;360:1–44.

75. Lindroth CH. The Ground-Beetles (Carabidae, Excluding Cicindelinae) of Canada and Alaska. Opuscula Entomologica [Internet]. 1961 [cited 2021 Nov 29];Supplementa XX, XXIV, XXIX, XXXIII, XXXIV, XXXV,. Available from: https://academic.oup.com/sysbio/article-lookup/doi/10.2307/2412348

76. Pinto JD. The taxonomy of North American Epicauta (Coleoptera: Meloidae), with a revision of the nominate subgenus and a survey of courtship behavior. Berkeley: University of California Press; 1991. 372 p. (University of California publications in entomology).

77. Smith ABT, Skelley PE. A New Species of Flaviellus Gordon and Skelley, 2007 (Coleoptera: Scarabaeidae: Aphodiinae) from the Yukon, Canada. The Coleopterists Bulletin. 2020 Mar 25;74(1):101.

78. Aldrich JM. North American two-winged flies of the genus Cylindromyia Meigen (Ocyptera of authors). Proceedings of the United States National Museum. 1926;68(2624):1–27.

79. Aldrich JM. A revision of the American parasitic flies belonging to the genus Belvosia. Proceedings of the United States National Museum. 1928;73(2729):1–45.

80. Ávalos-Hernández O. A Review Of The North American Species Of Hemipenthes Loew, 1869 (Diptera: Bombyliidae). 2009 Dec 31 [cited 2022 Jan 28]; Available from: https://zenodo.org/record/187152

81. Brooks AR. A REVIEW OF THE CANADIAN SPECIES OF ERNESTIA SENS. LAT. (TACHINIDAE, DIPTERA). Can Entomol. 1943 Apr;75(4):66–78.

82. Brooks AR. A REVIEW OF THE NORTH AMERICAN SPECIES OF GONIA sens. lat. (DIPTERA, TACHINIDAE). Can Entomol. 1943 Dec;75(12):219–36.

83. Brooks AR. A REVISION OF THE NORTH AMERICAN SPECIES OF THE RHODOGYNE COMPLEX (DIPTERA, LARVAEVORIDAE). Can Entomol. 1945 Dec;77(12):218–30.

84. Burt T. Taxonomic revision of four Nearctic Conopidae (Insecta: Diptera) genera (Dalmannia, Roberstonomyia, Stylogaster and Zodion) with notes on all other Nearctic genera [Internet]. [Ottawa, Ontario]: Carleton University; 2015. Available from: https://curve.carleton.ca/system/files/etd/83a38375-1d49-4c32-8c2c-2b0f0b9301a1/etd_pdf/1c9eb60a06629211d3fe3d6e7f5ffdff/burt-taxonomicrevisionoffournearcticconopidaeinsecta.pdf

85. Camras S. Notes on the North American species of the Zodion fulvifrons group (Diptera: Conopidae). The Pan-Pacific Entomologist. 1944;20(4):121–8.

86. Camras S. A Study of the Genus Occemyia in North America (Diptera: Conopidae)1. Annals of the Entomological Society of America. 1945 Jun 1;38(2):216–22.

87. Camras S. A Review of the New World Physocephala (Diptera: Conopidae). Annals of the Entomological Society of America. 1957 May 1;50(3):213–8.

88. Cole FR. A revision of the North American two-winged flies of the family Therevidae. Proceedings of the United States National Museum. 1923;62(2450):1–140.

89. Coquillett DW. Revision of the Tachinidae of America north of Mexico : a family of parasitic two-winged insects / [Internet]. Washington : Government Printing Office,; 1897 [cited 2022 Jan 27]. Available from: http://www.biodiversitylibrary.org/bibliography/87236

90. Curran CH. Revision of the Pipiza group of the family Syrphidae (flower-flies) from north of Mexico. Proceedings of the California Academy of Sciences. 1921;11(16):345–93.

91. Curran CH. Notes on the Genus Pipizella Rondani, with Descriptions of New Species (Diptera; Syrphidae). Transactions of the American Entomological Society. 1923;49(4):339–45.

92. Curran CH. The families and genera of North American Diptera. American Museum Novitates [Internet]. 1935 [cited 2022 Jan 28];812. Available from: http://www.biodiversitylibrary.org/bibliography/6825

93. Curran CH. The species of Temnostoma related to bombylans Linné (Syrphidae, Diptera). American Museum Novitates. 1939;1040.

94. Curran CH. New American Syrphidae. Bulletin of the American Museum of Natural History. 1941;78(3):243–304.

95. Curran CH, Fluke CL. Revision of the nearctic species of Helophilus and allied genera. Transactions of the Wisconsin Academy of Sciences, Arts and Letters. 1926;207–81.

96. Fluke CL, Weems JrHV. The Myoleptini of the Americas (Diptera, Syrphidae). American Museum Novitates. 1956;1758:1–23.

97. Foster GA, Mathis WN. A revision of the nearctic species of the genus Trixoscelis Rondani (Diptera: Heleomyzidae: Trixoscelidinae). Smithsonian Contributions to Zoology. 2012;(637):1–128.

98. Gibson JF. An updated and annotated checklist of the thick-headed flies (Diptera: Conopidae) of British Columbia, the Yukon, and Alaska. Journal of the Entomological Society of British Columbia. 2017;114.

99. Gill GD. The Heleomyzid Flies of American North of Mexico (Diptera: Heleomyzidae). Proceedings of the United States National Museum. 1962;113(3465):495–603.

100. Hardy E. New Nearctic Pipunculidae (Diptera). Journal of the Kansas Entomological Society. 1943;12(1):16–25.

101. Huckett HC. A Review of the North American Species Belonging to the Genus Hydrotaea Robineau-Desvoidy (Diptera, Muscidae). Annals of the Entomological Society of America. 1954 Jun 1;47(2):316–42.

102. James MT. The Genus Odontomyia in America North of Mexico (Diptera, Stratiomyidae). Annals of the Entomological Society of America. 1936 Sep 1;29(3):517–50.

103. James MT. The Status of Odontomyia arcuata Loew, O. inaequalis Loew, and Their Close Relatives in Western North America (Diptera: Stratiomyidae). Journal of the Kansas Entomological Society. 1974;47(2):222–6.

104. James MT, Steyskal GC. A Review of the Nearctic Stratiomyini (Diptera, Stratiomyidae). Annals of the Entomological Society of America. 1952 Sep 1;45(3):385–412.

105. Kits JH, Marshall SA, Evenhuis NL. The Bee Flies (Diptera: Bombyliidae) of Ontario, with a Key to the Species of Eastern Canada. CJAI. 2008;6.

106. Malloch JR. Diptera from the South-Western United States. Paper IV. Anthomyiidae. Transactions of the American Entomological Society. 1918;44(3):263–319.

107. Malloch JR. A SYNOPTIC REVISION OF THE ANTHOMYIID GENUS HYDROPHORIA ROBINEAU-DESVOIDY (DIPTERA). Can Entomol. 1920 Nov;52(11):253–7.

108. Malloch JR. A SYNOPSIS OF THE NORTH AMERICAN SPECIES OF THE GENUS HELINA R.-D., SENS. LAT. (DIPTERA, ANTHOMYIIDAE). Can Entomol. 1921 May;53(5):103–9.

109. Martin CH. The Holopogon complex of North America, excluding Mexico, with the descriptions of a new genus and a new subgenus (Diptera, Asilidae). American Museum Novitates. 1959;1980.

110. McAlpine JF, editor. Manual of Nearctic Diptera. Vol. 1. Ottawa: Research Branch, Agriculture Canada; 1981. (Monograph / Research Branch, Agriculture Canada; vol. 1).

111. McAlpine JF, editor. Manual of Nearctic Diptera. Vol. 2. Repr. Hull, Que: Canadian Gornvment Publ. Centre; 1993. 675 p. (Monograph / Research Branch, Agriculture Canada; vol. 2).

112. Miranda GFG, Young AD, Locke MM, Marshall SA, Skevington JH, Thompson FC. Key to the Genera of Nearctic Syrphidae. CJAI. 2013;23:1–351.

113. O'Hara JE. Classification, phylogeny and zoogeography of the North American species of Siphona Meigen (Diptera: Tachinidae). Department of Entomology, University of Alberta. 1982;18:261–380.

114. O'Hara JE, Wood DM. Checklist of the Tachinidae (Diptera) of America north of Mexico. Online [Internet]. 2004; Available from: http://www.nadsdiptera.org/Tach/WorldTachs/TTimes/Tach18.html

115. Sabrosky CW. The Chloropidae of Kansas (Diptera). Transactions of the American Entomological Society. 1935;61(3):207–68.

116. Sabrosky CW. A Third Species of Eusiphona, with Remarks on the Systematic Position of the Genus (Diptera, Milichiidae). Entomological News. 1955;66(7):169–73.

117. Sabrosky CW. Notes on the tachinid genus Cylindromyia in North America. Proceedings of the Entomological Society of Washington. 1967;69(1):60–3.

118. Schlinger EI. A Revision of the Genus Ogcodes Latreille with Particular Reference to Species of the Western Hemisphere. Proceedings of the United States National Museum. 1960;111(3429):227–336.

119. Sedman YS. The Chrysogaster (Orthonevra) pictipennis group in North America (Diptera: Syrphidae). Entomological Society of Washington. 1966;68:185–94.

120. Shannon RC. Review of the American xylotine syrphid-flies. Proceedings of the United States National Museum. 1926;69(2635):1–52.

121. Shannon RC. Temnostoma bombylans and related species (Syrphidae, Diptera). Entomological Society of Washington. 1939;41(7):215–24.

122. Skevington JH, Thompson FC. Review of New World Sericomyia (Diptera: Syrphidae), including description of a new species. Can Entomol. 2012 Apr;144(2):216–47.

123. Strickland EH. AN ANNOTATED LIST OF THE DIPTERA (FLIES) OF ALBERTA. Can J Res. 1938 Jul 1;16d(7):175–219.

124. Sun X, Marshall SA. Systematics of Phasia Latreille (Diptera: Tachinidae). Zootaxa. 2003 Aug 29;276(1):1.

125. Telford HS. Eristalis (Diptera: Syrphidae) from America North of Mexico1. Annals of the Entomological Society of America. 1970 Sep 15;63(5):1201–10.

126. Thompson FC. Revisionary notes on Nearctic Microdon flies (Diptera: Syrphidae). Proceedings of the Entomological Society of Washington. 1981;83:725–58.

127. Thompson FC, Fee FD, Bezark LD. Two Immigrant Synanthropic Flower Flies (Diptera: Syrphidae) New to North America. Entomological News. 1990;101(2):69–74.

128. Townsend CHT. NOTES ON NORTH AMERICAN TACHINIDÆ, WITH DESCRIPTIONS OF NEW GENERA AND SPECIES. Paper II. Can Entomol. 1891;18(4):349–82.

129. Townsend CHT. The taxonomy of the muscoidean flies, including descriptions of new genera and species. Smithsonian Miscellaneous Collections. 1908;51(2):1–138.

130. Vockeroth JR. The Flower Flies of the Subfamily Syrphinae of Canada, Alaska, and Greenland: Diptera: Syrphidae. Ottawa, Ont: Centre for Land and Biological Resources Research, Research Branch, Agriculture Canada; 1992. 456 p. (The Insects and arachnids of Canada).

131. Webb DW, Gaimari SD, Hauser M, Holston KC, Metz MA, Irwin ME, et al. An annotated catalogue of the New World Therevidae (Insecta: Diptera: Asiloidea). Zootaxa. 2013 Jan 11;3600(1):1–105.

132. Whitworth T. Keys to the Genera and Species of Blow Flies (Diptera: Calliphoridae) of America, North of Mexico. Proceedings of the Entomological Society of Washington. 2006;108(3):689–725.

133. Wilder DD. Systematics of the Nearctic Ptilodexia Brauer and Bergenstamm (Diptera, Tachinidae). Proceedings of the California Academy of Sciences. 1979;42(1):55.

134. Young AD, Marshall SA, Skevington JH. Revision of Platycheirus Lepeletier and Serville (Diptera: Syrphidae) in the Nearctic north of Mexico. Zootaxa. 2016 Feb 17;4082(1):1.

135. Cockerell TDA. The Bees of the Genus Perdita F. Smith. Proceedings of the Academy of Natural Sciences of Philadelphia. 1896;48:25–107.

136. Cockerell TDA. New species of Andrena from North America. The Entomologist. 1897;30:305–9.

137. Cockerell TDA. North American bees of the Genus Andrena. Annals and Magazine of Natural History. 1902 Feb;9(50):101–6.

138. Cockerell TDA. THE BEES OF ALBERTA.—I. Can Entomol. 1936 Dec;68(12):274–7.

139. Cockerell TDA. Bees of the Genus Sphecodes from Saskatchewan. Vol. 909. The American Museum of Natural History; 1937.

140. Cockerell TDA. THE BEES OF ALBERTA—IV. Can Entomol. 1937 May;69(5):113–4.

141. Cockerell TDA. BEES OF ALBERTA. V. Can Entomol. 1937 Jun;69(6):126–7.

142. Cresson ET. A list of the North American species of the genus Anthophora, with descriptions of new species. In Transactions of the American Entomological Society; 1869. p. 289–93.

143. Cresson ET. Descriptions of new species of North American bees. In Proceedings of the Academy of Natural Sciences of Philadelphia; 1878. p. 181–221.

144. Droege S, Rightmyer MG, Sheffield CS, Brady SG. New synonymies in the bee genus Nomada from North America (Hymenoptera: Apidae). Zootaxa. 2010 Oct 29;2661(1):1.

145. Droege S, Jean R, Orr M. Bee Genera of Eastern North America [Internet]. Apoidea - Discover Life. 2021. Available from: https://www.discoverlife.org/20/q?search=Apoidea

146. Dumesh S, Sheffield CS. Bees of the Genus Dufourea Lepeletier (Hymenoptera: Halictidae: Rophitinae) of Canada. CJAI. 2012 May;18.

147. Dumesh S, Sheffield CS. Photographic keys to the bees of the Northwest Territories, Canada. Department of Environment and Natural Resources, Government of the Northwest Territories.; 2014. 257 p.

148. Gibbs J. Revision of the metallic species of Lasioglossum (Dialictus) in Canada (Hymenoptera, Halictidae, Halictini). Zootaxa. 2010 Aug 31;2591(1):1.

149. Hurd PD, Michener CD. The Megachiline bees of California (Hymenoptera : Megachilidae). University of California Press Berkeley and Los Angeles; 1955. 251 p. (Bulletin of the California Insect Survey; vol. 3).

150. LaBerge WE. A Revision of the Bees of the Genus Melissodes in North and Central America. Parts I., II. (Hymenoptera, Apidae). The University of Kansas science bulletin. 1956;37:911–1194.

151. LaBerge WE. A Revision of the Bees of the Genus Melissodes in North and Central America. Part III (Hymenoptera, Apidae). The University of Kansas science bulletin. 1961;42:283–663.

152. McGinley RJ. Studies of Halictinae (Apoidea: Halictidae), I: Revision of New World Lasioglossum Curtis. Smithsonian Contributions to Zoology. 1986;(429):1–294.

153. Michener CD. The Bees of the World. 2nd ed. Baltimore: Johns Hopkins University Press; 2007. 953 p.

154. Mitchell TB. New Species of Sphecodes from the Eastern United States. Journal of the Elisha Mitchell Scientific Society. 1956;72(2):206–22.

155. Mitchell TB. Bees of the Eastern United States, V1. Raleigh: North Carolina Agricultural Experiment Station Technical Bulletin; 1960. 538 p.

156. Mitchell TB. Bees of the Eastern United States, V2. Raleigh: North Carolina Agricultural Experiment Station Technical Bulletin; 1962. 557 p.

157. Mitchell TB. A subgeneric revision of the bees of the genus Coelioxys of the Western Hemisphere (Hymenoptera: Megachilidae). Department of Entomology, North Carolina State University; 1973. 129 p.

158. Onuferko TM. Cleptoparasitic bees of the genus Epeolus Latreille (Hymenoptera: Apidae) in Canada. CJAI. 2017;30:1–62.

159. Packer L, Genaro JA, Sheffield CS. The Bee Genera of Eastern Canada. CJAI. 2007;3:1–32.

160. Rightmyer MG. A review of the cleptoparasitic bee genus Triepeolus (Hymenoptera: Apidae).-Part I. Zootaxa. 2008 Feb 22;1710(1):1.

161. Roberts RB. Bees of northwestern America: Agapostemon (Hymenoptera: Halictidae). Technical Bulletin of the Agricultural Experiment Station. 1973;125:1–23.

162. Roberts RB. Bees of northwestern America: Halictus (Hymenoptera: Halictidae). Technical Bulletin of the Agricultural Experiment Station [Internet]. 1973;126. Available from: https://static1.squarespace.com/static/5a849d4c8dd041c9c07a8e4c/t/5a9727410d9297d03d5f566c/1519855427669/Roberts+1973+Bees+of+Northwestern+America+Halictus.pdf

163. Sheffield CS, Ratti C, Packer L, Griswold T. Leafcutter and Mason Bees of the Genus Megachile Latreille (Hymenoptera: Megachilidae) in Canada and Alaska. CJAI. 2011 Nov 29;18.

164. Sheffield CS, Frier SD, Dumesh, S. The Bees (Hymenoptera: Apoidea, Apiformes) of the Prairies Ecozone with Comparisons to other Grasslands of Canada. 2014;427–67.

165. Stephen WP. A Revision of the Bee Genus Colletes in America North of Mexico (Hymenoptera Colletidae). The University of Kansas Science Bulletin. 1954;36(6):149–527.

166. Viereck HL, Cockerell TDA. New North American bees of the genus Andrena. Proceedings of the United States National Museum. 1914;48(2064):1–58.

167. Williams P, Thorp RW, Richardson L, Colla S. Bumble bees of North America: an identification guide. Princeton: Princeton University Press; 2014. 208 p.

168. York University. Key to the Genera of Andreninae [Internet]. York University; Available from: https://www.yorku.ca/bugsrus/resources/keys/Andreninae/Images/Andreninae_A_Start/Andreninae_A_Start_Export.htm

169. Goulet H, Huber JT, editors. Hymenoptera of the world: an identification guide to families. Ottawa, Ontario: Centre for Land and Biological Resources Research; 1993. 668 p. (Publication).

170. Kimsey L, Carpenter J. The Vespinae of North America (Vespidae, Hymenoptera). JHR. 2012 Aug 24;28:37–65.

171. Bain J, Flanagan J, Kuigt J. Common Coulee Plants of Southern Alberta. 2nd ed. University of Lethbridge Herbarium.; 2014.

172. Moss EH, Packer JG. Flora of Alberta [Internet]. 2nd ed. University of Toronto Press; 1994. Available from: http://www.jstor.org/stable/10.3138/j.ctt1287xvf

173. Tannas K. Common plants of the western rangelands, volume 2: Trees and Shrubs. Olds College; 2003.

174. Tannas K. Common plants of the western rangelands, volume 2: Forbs. Olds College; 2004.

Supporting Information

Description of Network Metrics Associated With Stability

Of the network metrics theoretically related to plant-pollinator network stability, **nestedness** has the strongest association. A highly nested network is one in which interactions of specialists are subsets of the interactions of generalists. In this way, the interactions in the network become highly asymmetrical and organized around a few generalist species (57). Plant-pollinator networks are generally nested when "binary" networks are considered (presence/absence of interactions only, as opposed to "quantitative" networks that also consider interaction frequencies), and this binary nested structure has been interpreted to minimize interspecific competition and enhance species coexistence, and to arise when new species enter the network where they have minimal competitive load (58). Binary nestedness increases plant-pollinator network stability (33), and almost all studies that have examined honey bee effects on nestedness concur that honey bees increase binary network nestedness (19–21) when honey bee interactions are included in the network, but the effect of honey bees on nestedness of just the wild pollinator-plant interactions has not been investigated. More recently, (51) showed that although most plant-pollinator networks are nested in their binary structure, they are not quantitatively nested, meaning that less abundant flower species are focused on by different pollinator species, such that niches are less overlapping than appears to be the case in the binary structure. Because we were interested in detecting honey bee effects on this kind of niche partitioning, we used the weighted version, WNODF, of the metric for nestedness, NODF, that has been used in previous studies of the effects of honey bees on nestedness (19–21).

Modularity (the extent to which interactions are clumped into semi-separate modules) is thought to decrease stability in plant-pollinator networks (33) and to be decreased by honey bees, because they introduce new links that join previously separate modules (19,20); but (26) found the opposite). We calculated modularity only for the full season all taxa and full season without-honey-bees datasets; as it was not significant in either, it was not calculated for other datasets.

Other network metrics are more tenuously linked to stability, such as **connectance** (number of observed interactions over total possible interactions) and **link density** (total number of interactions over the total number of nodes, or the mean number of interactions per species). Both are expected to increase stability (33) and be decreased by honey bees (17,26,59). (17) compared effects of honey bees on link density of the total network and effects of honey bees on link density of just wild pollinator interactions, and found that the effect of honey bees on this metric decreased dramatically when the honey bee interactions were not included in the calculation of the metric. Nevertheless, they found a significant but small decrease in link density of wild pollinator interactions as honey bee density increased.

Network metrics of **interaction strength asymmetry** (the asymmetrical strength of one species interaction relative to the reciprocal interaction, averaged across all species pairs in the network) and **interaction evenness** (the uniformity of the interaction frequencies distributed through the network, calculated using Shannon's evenness index) may also be related to stability. Interaction strength asymmetry has been found to increase with increasing honey bee abundance, as honey bees dominate interactions with wild plants (59). However, interaction evenness has been found to decrease with increasing honey bee abundance, as the distribution of interactions was skewed

toward honey bees (17) and even for plant-pollinator networks excluding honey bees ((22), though this study identified taxa into broad groups rather than providing species-level resolution, so network metrics may not be comparable). While interaction strength asymmetry is thought to be stabilizing, interaction evenness may be destabilizing, though its relationship to stability has only been tested in antagonistic networks, not in mutualistic networks (60).

Justifications For the Predictions Shown in Figure 1b.

In Fig 1b, we show our predictions as to how each network metric will change as honey bee abundance increases (going from transects far from honey bee hives to transects close to honey bee hives). Because we added honey bees in high enough densities that they would be involved in a large proportion of all plant-flower visitor interactions, for certain network metrics, we anticipated that increasing honey bee abundance would affect the with-honey bee versus without-honey bee networks in different ways (yellow vs. blue lines in Fig 1b).

Resource Overlap Metrics

For the resource overlap metrics all of our predictions stem from our hypothesis that introduced honey bees would compete with many other pollinator species, excluding some of these species from some of their floral resources, and narrowing their floral niches. We predicted decreases in **pollinator niche overlap** (a univariate index of similarity in the set of flower species visited by different pollinator species), **plant niche overlap** (univariate similarity in the set of pollinator species visiting each plant species), **generality** (the mean number of plant species visited by each pollinator species), **vulnerability** (the mean number of pollinator species visiting each plant species), and increases in **pollinator functional complementarity** (the multivariate dissimilarity

of the set of species that each pollinator species interacts with) and **plant functional complementarity** (multivariate dissimilarity of the set of species that each plant species interacts with) (17).

Stability-Related Metrics For Networks Including Honey Bees (Yellow Trend Lines in Fig 1b)

Our predictions for the effects of varying honey bee abundance on the set of stability-related metrics are based on the findings of previous studies. We expected networks from transects with higher honey bee abundance to have an increase in **nestedness** (**WNODF**) (19–21,23,50), either an increase (26) or a decrease (19,20) in **modularity**, decreases in **connectance** (26,59), **link density** (17,26,59), and **interaction evenness** (17), and either no change (26) or an increase (59) in **interaction strength asymmetry**.

Stability-Related Metrics For Networks Excluding Honey Bees (Blue Trend Lines in Fig 1b)

With honey bees removed from the network we predicted no change in WNODF and interaction strength asymmetry, an increase in modularity, and decreases in connectance, link density (17), and interaction evenness (22).

Calculation of Network Metrics Associated With Stability

The first network metric for stability, **weighted nestedness** based on overlap and decreasing fill (WNODF, hereinafter referred to as “nestedness”) is a measurement of the degree to which interactions of specialists are subsets of the interactions of generalists (61). We adopted the

methods used by (26) to calculate **modularity** using DIRT_LBA_wb_plus which computes modules with **Newman's modularity measure** (bipartite package, (29,62)). We ran 50 trials for each network matrix and retained the output to two decimal places. **Interaction strength asymmetry** describes, on average, how asymmetric the interactions are between interacting species pairs from each trophic level, where strength is the frequency of visits (63). We used the "Bluethgen" method to calculate this metric (64). Similarly, **interaction evenness** measures the evenness of the frequency of unique interactions using **Shannon's evenness** (41,65). **Weighted connectance** is a calculation of the proportion of interactions relative to the number of possible interactions in a network, and is therefore calculated by dividing the number of links by the number of cells in the species interaction matrix (29). Lastly, **link density** measures the mean number of interactions per pollinator species weighted by the average number of interactions of each species (41,65).

Rationale For Using Both SLR and MR Models in the Statistical Analysis

The purpose of using both an SLR and an MR to analyze honey bee effects on each response variable is because it is statistically impossible to separate any correlated effects of honey bee abundance and flower community variables (flower abundance and flower species richness) on our response variables (network metrics). In an SLR, any effect on the response variable possibly attributable to honey bee abundance is attributed fully to honey bee abundance. Conversely, in the MR model any effect on the response variable that is statistically indistinguishable (because of multicollinearity) between an effect due to honey bees or an effect due to flower community

variables, is not attributed to honey bees (47). Thus, the reason to include the SLR model in addition to the more conservative MR model is because the part of the effect of the predictor variables on the response variable that cannot be attributed to solely honey bee abundance, may in reality be due to honey bee abundance, but our methods cannot detect this. So, providing the SLR as well as the MR allows us to see what the maximum possible effect of honey bee abundance was in this study (regression coefficient from the SLR) versus the more conservative but unambiguous effect of honey bee abundance (partial regression coefficient from the MR) (47).

Supplementary Tables & Figures

Table S1. Longitude and latitude for each hive location and sampling transect, and collection effort at each transect. The northernmost, central, and southernmost hive locations are listed as Bee48, Bee32, and Bee16 respectively, indicating their number of hives. Each transect is indicated by its treatment (100 m, 500 m, or 5000 m distance from a hive location). Letters indicate each replicated set of distances from hives (See Figure 1). G5000 indicates the new location sampled at once sampling at F5000 was discontinued mid-season.

**Longitude**	**Latitude**	**Transect**	**Number of Collections**
-111.91850	50.90210	Bee48	
-111.94658	50.88120	Bee32	
-111.93580	50.84320	Bee16	
-111.91992	50.90202	A100	10
-111.92545	50.90248	A500	10
-111.98687	50.91594	A5000	8
-111.91699	50.90205	B100	7
-111.91010	50.90265	B500	6
-111.85447	50.88185	B5000	8
-111.94792	50.88178	C100	9
-111.95325	50.88365	C500	8
-112.00554	50.90691	C5000	8
-111.94533	50.88081	D100	9
-111.94149	50.87831	D500	9
-111.87483	50.86788	D5000	8
-111.93735	50.84338	E100	3
-111.94283	50.84407	E500	8
-111.99767	50.93795	E5000	7
-111.93415	50.84310	F100	8
-111.92928	50.84510	F500	7
-111.86687	50.83544	F5000	2
-111.98858	50.95827	G5000	5

Table S2. List of references and resources used in species identifications. Full citations are listed in the References section of the Supporting Information.

Insect Group	References	Museum Resources	Expert Taxonomists
Butterflies (Lepidoptera - Papilionoidea)	• Acorn (1993) - (66)<br>• Vandyk (2021) - (67)<br>• Warren et al (2016) - (68)	• University of Alberta Strickland Museum	John Acorn
Moths (Lepidoptera)		• Northern Forestry Centre	Greg Pohl
Ants (Hymenoptera – Formicoidea)	• Glasier et al (2013) - (69)		James Glasier
Beetles (Coleoptera)	• Acorn (2001) - (70)<br>• Acorn (2007) - (71)<br>• Arnett and Thomas (2000) - (72)<br>• Arnett et al (2002) - (73)<br>• Bousquet et al (2013) - (74)<br>• Lindroth (1961) - (75)<br>• Pinto (1991) - (76)<br>• Smith and Skelley (2020) - (77)<br>• Vandyk (2021) - (67)	• University of Alberta Strickland Museum	John Acorn
Flies (Diptera)	• Aldrich (1926) - (78)<br>• Aldrich (1928) - (79)<br>• Ávalos-Hernández (2009) - (80)<br>• Brooks (1943a) - (81)<br>• Brooks (1943b) - (82)<br>• Brooks (1945) - (83)<br>• Burt (2015) - (84)<br>• Camras (1944) - (85)<br>• Camras (1945) - (86)<br>• Camras (1957) - (87)<br>• Cole (1923) - (88)<br>• Coquillett (1897) - (89)<br>• Curran (1921) - (90)<br>• Curran (1923) - (91)<br>• Curran (1935) - (92)<br>• Curran (1939) - (93)<br>• Curran (1941) - (94)<br>• Curran and Fluke (1926) - (95)<br>• Fluke and Weems (1956) - (96)<br>• Foster and Mathis (2012) - (97)<br>• Gibson (2017) - (98)<br>• Gill (1962) - (99)<br>• Hardy (1943) - (100)	• University of Alberta Strickland Museum	Brittany Wingert

	• Huckett (1954) - (101)<br>• James (1936) - (102)<br>• James (1974) - (103)<br>• James and Steyskal (1952) - (104)<br>• Kits et al. (2008) - (105)<br>• Malloch (1918) - (106)<br>• Malloch (1920) - (107)<br>• Malloch (1921) - (108)<br>• Martin (1959) - (109)<br>• McAlpine (1981) - (110)<br>• McAlpine (1993) - (111)<br>• Miranda et al (2013) - (112)<br>• O'Hara (1982) - (113)<br>• O'Hara and Wood (2004) - (114)<br>• Sabrosky (1935) - (115)<br>• Sabrosky (1955) - (116)<br>• Sabrosky (1967) - (117)<br>• Schlinger (1960) - (118)<br>• Sedman (1966) - (119)<br>• Shannon (1926) - (120)<br>• Shannon (1939) - (121)<br>• Skevington and Thompson (2012) - (122)<br>• Strickland (1938) - (123)<br>• Sun and Marshall (2003) - (124)<br>• Telford (1970) - (125)<br>• Thompson (1981) - (126)<br>• Thompson et al (1990) - (127)<br>• Townsend (1891) - (128)<br>• Townsend (1908) - (129)<br>• Vockeroth (1992) - (130)<br>• Webb et al (2013) - (131)<br>• Whitworth (2006) - (132)<br>• Wilder (1979) - (133)<br>• Young et al (2016) - (134)		
Bees (Hymenoptera - Anthophila)	• Cockerell (1896) - (135)<br>• Cockerell (1897) - (136)<br>• Cockerell (1902) - (137)<br>• Cockerell (1936) - (138)<br>• Cockerell (1937a) - (139)<br>• Cockerell (1937b) - (140)<br>• Cockerell (1937c) - (141)<br>• Cresson (1869) - (142)<br>• Cresson (1878) - (143)<br>• Droege et al (2010) - (144)<br>• Droege et al (2021) - (145)	• University of Alberta Strickland Museum<br>• University of Calgary Museum of Zoology	Lincoln Best

	• Dumesh and Sheffield (2012) - (146)<br>• Dumesh and Sheffield (2014) - (147)<br>• Gibbs (2010) - (148)<br>• Hurd and Michener (1955) - (149)<br>• LaBerge (1956) - (150)<br>• LaBerge (1961) - (151)<br>• McGinley (1986) - (152)<br>• Michener (2007) - (153)<br>• Mitchell (1956) - (154)<br>• Mitchell (1960) - (155)<br>• Mitchell (1962) - (156)<br>• Mitchell (1973) - (157)<br>• Onuferko (2017) - (158)<br>• Packer et al (2007) - (159)<br>• Rightmyer (2008) - (160)<br>• Roberts (1973a) - (161)<br>• Roberts (1973b) - (162)<br>• Sheffield et al. (2011) - (163)<br>• Sheffield et al. (2014) - (164)<br>• Stephen (1954) - (165)<br>• Viereck and Cockerell (1914) - (166)<br>• Williams et al (2014) - (167)<br>• York University (n.d.) - (168)		
Wasps (Hymenoptera - Apocrita)	• Goulet and Huber (1993) - (169)<br>• Kimsey and Carpenter (2012) - (170)	Royal Alberta Museum	Matthias Buck
Plants	• Bain et al (2014) - (171)<br>• Moss (1994) - (172)<br>• Tannas (2003) - (173)<br>• Tannas (2004) - (174)		

Table S3. Pearson correlations between the response variables (network metrics). Each row and column header refers to the following metrics, respectively: G, generality; V, vulnerability; Poll. NO, pollinator niche overlap; Pl. NO, plant niche overlap; Poll. FC, pollinator functional complementarity; Pl. FC, plant functional complementarity; WNODF, weighted nestedness based on overlap and decreasing fill; M, modularity; WC, weighted connectance; LD, link density; ISA, interaction strength asymmetry; IE, interaction evenness.

	G	V	Poll. NO	Pl. NO	Poll. FC	Pl. FC	WNODF	M	WC	LD	ISA	IE
**G**		0.2697	-0.6359	0.6946	0.2326	0.1871	0.6796	0.4069	-0.5348	0.3489	-0.6631	0.1584
**V**	0.2697		0.3847	0.1610	0.0598	-0.0021	0.1004	0.1328	0.1491	0.9965	0.2703	0.4320
**Poll. NO**	-0.6359	0.3847		-0.2876	-0.2328	-0.2307	-0.2577	-0.5865	0.7461	0.3195	0.7857	0.1155
**Pl. NO**	0.6946	0.1610	-0.2876		0.1473	0.1077	0.7357	0.0417	-0.2985	0.2167	-0.3601	0.0293
**Poll. FC**	0.2326	0.0598	-0.2328	0.1473		0.9922	0.0476	0.0006	-0.7518	0.0783	-0.2365	-0.7252
**Pl. FC**	0.1871	-0.0021	-0.2307	0.1077	0.9922		0.0147	-0.0267	-0.7322	0.0141	-0.2439	-0.7601
**WNODF**	0.6796	0.1004	-0.2577	0.7357	0.0476	0.0147		-0.0233	-0.2743	0.1564	-0.5198	0.0206
**M**	0.4069	0.1328	-0.5865	0.0417	0.0006	-0.0267	-0.0233		-0.4000	0.1644	-0.3664	0.4278
**WC**	-0.5348	0.1491	0.7461	-0.2985	-0.7518	-0.7322	-0.2743	-0.4000		0.0989	0.6602	0.5171
**LD**	0.3489	0.9965	0.3195	0.2167	0.0783	0.0141	0.1564	0.1644	0.0989		0.2058	0.4341
**ISA**	-0.6631	0.2703	0.7857	-0.3601	-0.2365	-0.2439	-0.5198	-0.3664	0.6602	0.2058		0.1623
**IE**	0.1584	0.4320	0.1155	0.0293	-0.7252	-0.7601	0.0206	0.4278	0.5171	0.4341	0.1623	

Table S4. Pearson correlations between predictor variables. “Collections” refers to the number of collection rounds at each transect, “Honey bee abundance” refers to honey bee abundance at each transect, “Flower species” refers to the number of flowering species at each transect, and “Flower abundance” refers to the number of individual flowers at each transect.

	Honey bee abundance	Collections	Flower species	Flower abundance
Honey bee abundance		0.4454	0.6178	0.8517
Collections	0.4454		0.4343	0.3848
Flower species	0.6178	0.4343		0.5981
Flower abundance	0.8517	0.3848	0.5981	

Table S5. Percent sampling completeness and 95% confidence intervals, calculated as the number of unique interactions observed, divided by the Chao1 estimated total number of unique interactions, and multiplied by 100, for the full season all taxa, mid-season all taxa, and full season bees-only datasets. The "Lower 95%" column indicates the observed interactions divided by the lowest Chao1 estimated total number of true interactions, and the "Upper 95%" column indicates the observed interactions divided by the highest Chao1 estimated total number of true interactions (which is why the "Lower" values are higher). Shaded cells denote networks that had too few species for some network metrics to be meaningful, so these replicates were excluded from analysis for the metrics: generality, vulnerability, plant and pollinator niche overlap, and nestedness.

Transect	Full Season Chao (%)	Full Season (Lower 95%)	Full Season (Upper 95%)	Mid-Season Chao (%)	Mid-Season (Lower 95%)	Mid-Season (Upper 95%)	Full Season Bees Only Chao (%)	Full Season Bees Only (Lower 95%)	Full Season Bees Only (Upper 95%)
**A100**	32.261	56.043	15.104	42.155	74.898	15.109	51.891	85.115	16.904
**A500**	37.002	60.298	18.510	21.937	54.374	6.215	41.172	73.312	15.133
**A5000**	30.264	55.443	13.146	31.239	62.984	10.818	58.120	85.569	24.515
**B100**	19.273	49.517	5.492	42.996	85.010	9.120	26.521	70.481	5.174
**B500**	37.534	66.456	15.415	NA	NA	NA	73.275	94.453	94.453
**B5000**	25.159	46.371	11.559	43.914	77.220	15.313	23.161	56.628	6.506
**C100**	46.666	66.435	27.892	25.828	60.420	7.358	71.861	90.536	40.544
**C500**	33.590	50.239	20.217	51.112	74.850	26.860	26.942	57.414	9.164
**C5000**	50.330	68.571	32.000	50.051	74.673	25.405	53.648	84.379	19.870
**D100**	21.306	42.350	9.073	21.265	47.044	7.588	33.229	65.748	11.428
**D500**	24.903	45.944	11.456	41.776	78.793	12.170	12.111	44.338	2.328
**D5000**	20.522	46.661	7.081	31.528	68.871	8.745	28.679	64.485	8.177
**E100**	44.262	80.830	13.009	NA	NA	NA	NA	NA	NA
**E500**	31.594	48.799	18.289	35.263	58.631	17.311	37.100	62.525	17.254
**E5000**	36.864	63.866	16.170	15.967	53.154	3.084	62.461	91.466	20.538
**F100**	26.546	52.593	10.533	46.701	76.693	18.917	42.938	79.319	12.865
**F500**	40.013	72.013	14.742	50.505	85.046	15.473	25.180	69.013	4.839
**F5000**	35.302	69.065	11.767	48.478	83.064	15.293	71.016	95.303	22.809
**G5000**	33.442	65.967	11.523	43.852	77.176	15.282	83.333	97.752	36.498

Table S6. Identifications of insect pollinators to species-level or morphospecies level. Morphospecies identifications are listed by “[Genus] sp. #”. Some species could not be differentiated between genera, and so both genera are listed along with the epithet “sp”. Specimens listed beside “cf” (confer, meaning compare with) are specimens that were damaged or for which taxonomic keys are insufficient, and these were compared to other specimens to determine identification. Numbers of each (morpho)species are given for each distance from hives, despite the fact that we used honey bee abundance, rather than distance from hive as the predictor variable in our analyses.

Group/ Order	Family	Species	100 m	500 m	5000 m	Total
**Anthophila**						
	Andrenidae	*Andrena amphibola*	1	0	0	1
	Andrenidae	*Andrena cyanophila*	0	2	0	2
	Andrenidae	*Andrena lupinorum*	0	1	1	2
	Andrenidae	*Andrena medionitens*	0	1	0	1
	Andrenidae	*Andrena peckhami*	0	3	6	9
	Andrenidae	*Andrena prunorum*	0	1	0	1
	Andrenidae	*Andrena* sp.1	0	1	0	1
	Andrenidae	*Andrena* sp.2	1	0	0	1
	Andrenidae	*Andrena thaspii*	4	1	2	7
	Andrenidae	*Panurginus beardsleyi*	0	2	0	2
	Andrenidae	*Perdita bruneri*	7	12	0	19
	Andrenidae	*Perdita swenki*	26	4	0	30
	Apidae	*Apis mellifera*	172	108	5	285
	Apidae	*Bombus borealis*	7	2	4	13
	Apidae	*Bombus fervidus*	1	0	0	1
	Apidae	*Bombus rufocinctus*	2	0	0	2
	Apidae	*Bombus ternarius*	0	1	1	2
	Apidae	*Epeolus compactus*	0	0	2	2
	Apidae	*Epeolus minimus*	0	5	4	9
	Apidae	*Holcopasites pulchellus*	0	1	0	1
	Apidae	*Melissodes* cf. *coreopsis*	3	2	4	9

Apidae	*Melissodes rivalis*	1	0	0	1
Apidae	*Neolarra pruinosa*	2	0	0	2
Apidae	*Neolarra vigilans*	1	0	0	1
Apidae	*Triepeolus helianthi*	0	1	1	2
Colletidae	*Colletes brevicornis*	0	18	23	41
Colletidae	*Colletes fulgidus*	3	0	1	4
Colletidae	*Colletes hyalinus*	28	46	15	89
Colletidae	*Colletes impunctatus*	6	11	6	23
Colletidae	*Colletes kincaidii*	6	11	10	27
Colletidae	*Colletes* cf. *petalostemonis*	2	0	0	2
Colletidae	*Colletes* cf. *simulans*	3	3	1	7
Colletidae	*Hylaeus annulatus*	2	0	0	2
Colletidae	*Hylaeus mesillae*	1	3	0	4
Halictidae	*Agapostemon femoratus*	1	0	0	1
Halictidae	*Agapostemon texanus*	1	0	2	3
Halictidae	*Agapostemon virescens*	1	1	0	2
Halictidae	*Dufourea maura*	0	1	10	11
Halictidae	*Halictus confusus*	5	7	4	16
Halictidae	*Halictus ligatus*	0	2	0	2
Halictidae	*Halictus rubicundus*	1	1	5	7
Halictidae	*Lasioglossum* cf. *albohirtum*	2	4	5	11
Halictidae	*Lasioglossum egregium*	0	1	0	1
Halictidae	*Lasioglossum hudsoniellum*	2	2	1	5
Halictidae	*Lasioglossum leucozonium*	0	2	1	3
Halictidae	*Lasioglossum paraforbesii*	0	0	1	1
Halictidae	*Lasioglossum pruinosum*	1	2	2	5
Halictidae	*Lasioglossum sagax*	2	0	0	2
Halictidae	*Lasioglossum* sp.1	2	1	2	5
Halictidae	*Lasioglossum* sp.2	0	0	6	6

Halictidae	*Lasioglossum* sp.3	0	1	0	1
Halictidae	*Lasioglossum succinipenne*	2	3	1	6
Halictidae	*Lasiogossum* cf. *rufulipes*	6	12	18	36
Megachilidae	*Anthidium clypeodentatum*	0	4	0	4
Megachilidae	*Coelioxys rufitarsus*	0	1	0	1
Megachilidae	*Hoplitis fulgida*	0	1	0	1
Megachilidae	*Hoplitis pilosifrons*	3	4	1	8
Megachilidae	*Hoplitis producta*	5	1	0	6
Megachilidae	*Hoplitis spoliata*	0	1	1	2
Megachilidae	*Megachile brevis*	2	1	2	5
Megachilidae	*Megachile circumcincta*	0	1	0	1
Megachilidae	*Megachile dentitarsus*	1	1	32	34
Megachilidae	*Megachile frigida*	0	1	0	1
Megachilidae	*Megachile inermis*	0	2	0	2
Megachilidae	*Megachile latimanus*	5	9	3	17
Megachilidae	*Megachile perihirta*	1	4	2	7
Megachilidae	*Megachile rotundata*	11	4	0	15
Megachilidae	*Megachile wheeleri*	0	1	0	1
Megachilidae	*Osmia distincta*	5	2	3	10
Megachilidae	*Osmia integra*	0	1	0	1
Megachilidae	*Osmia simillima*	10	9	5	24
Megachilidae	*Osmia* sp.1	0	1	0	1
Megachilidae	*Stelis lateralis*	0	2	0	2
**Coleoptera**					
Anthicidae	*Notoxus* cf. *anchora*	10	2	10	22
Chrysomelidae	*Acanthoscelides* sp.	0	0	1	1
Chrysomelidae	*Erynephala* cf. *puncticollis*	1	0	0	1
Cleridae	*Phyllobaenus humeralis*	4	25	8	37
Cleridae	*Trichodes nutalli*	1	0	2	3

	Coccinellidae	*Brachiacantha albifrons*	0	1	0	1
	Meloidae	*Epicauta ferruginea*	0	1	0	1
	Meloidae	*Epicauta pruinosa*	0	1	0	1
	Meloidae	*Epicauta puncticollis*	0	1	0	1
	Meloidae	*Epicauta subglabra*	0	1	5	6
	Melyridae	*Collops vittatus*	0	1	0	1
	Melyridae	*Listrus* sp.	0	2	0	2
	Mordellidae	*Mordella atrata*	0	2	1	3
	Staphylinidae	*Philonthus caerulipennis*	1	7	10	18
**Diptera**						
	Acroceridae	*Ogcodes eugonatus*	2	0	0	2
	Anthomyiidae	*Adia* sp.	0	0	1	1
	Anthomyiidae	*Adia* or *Paregle* sp.	0	0	1	1
	Anthomyiidae	*Botanophila* sp.	1	0	0	1
	Anthomyiidae	*Delia* sp.	0	0	5	5
	Anthomyiidae	*Delia* or *Lasiomma* sp.	0	0	1	1
	Anthomyiidae	*Fucellia* sp.	0	2	0	2
	Anthomyiidae	*Fucellia* or *Delia* sp.	0	1	0	1
	Anthomyiidae	*Hydrophoria* or *Delia* sp.	0	1	0	1
	Anthomyiidae	*Pegohylemyia* or *Delia* or *Lasiomma* sp.	0	1	1	2
	Anthomyiidae	*Pegohylemyia* or *Delia* sp.	0	0	5	5
	Anthomyiidae	*Pegohylemyia* or *Lasiomma* sp.	1	0	0	1
	Anthomyiidae	*Pegomya* sp.	0	1	1	2
	Anthomyiidae	*Pegomya* or *Adia* sp.	0	0	1	1
	Anthomyiidae	*Pegoplata* sp.	1	1	1	3
	Anthomyiidae	*Phorbia* or *Hydrophoria* or *Pegohylemyia* sp.	1	0	1	
	Asillidae	*Dicropaltum mesae*	0	1	0	1
	Asillidae	*Holopogon albopilosa*	1	0	1	2

Bombyliidae	*Anastoechus barbatus*	12	18	4	34
Bombyliidae	*Anastoechus melanohalteralis*	2	6	1	9
Bombyliidae	*Anthrax picea*	1	0	0	1
Bombyliidae	*Chrysanthrax costata*	0	0	1	1
Bombyliidae	*Geron* sp.	0	1	0	1
Bombyliidae	*Hemipenthes morio*	4	2	0	6
Bombyliidae	*Hemipenthes sinuosa*	0	0	1	1
Bombyliidae	*Poecilanthrax alcyon*	0	1	1	2
Bombyliidae	*Poecilanthrax monticola*	0	3	0	3
Bombyliidae	*Poecilanthrax tegminipennis*	0	1	11	12
Bombyliidae	*Poecilanthrax willistonii*	0	2	0	2
Bombyliidae	*Poecilognathus* sp.	0	0	1	1
Bombyliidae	*Systoechus vulgaris*	4	10	2	16
Bombyliidae	*Villa fulviana*	1	1	3	5
Bombyliidae	*Villa lateralis*	2	2	1	5
Bombyliidae	*Villa lateralis* spgr.	0	2	0	2
Calliphoridae	*Phormia regina*	1	0	0	1
Calliphoridae	*Protophormia terraenovae*	0	0	1	1
Conopidae	*Thecophora occidensis*	0	1	0	1
Conopidae	*Zodion cinereiventre*	0	0	2	2
Conopidae	*Zodion fulvifrons*	0	1	1	2
Conopidae	*Zodion hitchensi*	0	1	0	1
Conopidae	*Zodion lisafyrea*	1	0	0	1
Conopidae	*Zodion* sp.	0	1	1	2
Dolichopodidae	sp. indet.	1	0	0	1
Milichiidae	*Eusiphona mira*	0	1	1	2
Milichiidae	*Pholeomyia* sp.	0	1	0	1
Muscidae	*Haematobia irritans*	0	1	0	1
Muscidae	*Hydrotaea meteorica*	1	1	0	2

Muscidae	*Neomyia cornicina*	0	2	2	4
Pipunculidae	sp. indet.	0	0	1	1
Sarcophagidae	*Ravinia* sp.	2	1	6	9
Sarcophagidae	*Ravinia* or *Arachnidomyia* sp.	0	0	1	1
Sarcophagidae	sp. indet.	2	2	3	7
Sarcophagidae	*Senotainia* sp.	1	9	1	11
Sarcophagidae	*Senotainia* or *Macronychia* sp.	0	2	0	2
Sarcophagidae	*Sphixapata trilineata*	0	1	0	1
Sarcophagidae	*Sphixapata triliniata*	0	3	0	3
Sarcophagidae	*Udamopyga niagarana*	0	1	0	1
Stratiomyidae	*Nemotelus* or *Camptopelta* sp.	1	0	0	1
Syrphidae	*Cheilosia* sp.	0	0	2	2
Syrphidae	*Copestylum marginatum*	1	0	0	1
Syrphidae	*Copestylum* sp.	0	1	0	1
Syrphidae	*Eristalis dimidiata*	0	0	1	1
Syrphidae	*Eristalis hirta*	1	0	1	2
Syrphidae	*Eristalis stipator*	15	16	14	45
Syrphidae	*Eristalis tenax*	0	0	1	1
Syrphidae	*Eupeodes* sp.	0	2	0	2
Syrphidae	*Eupeodes volucris*	2	1	1	4
Syrphidae	*Helophilus hybridus*	3	1	1	5
Syrphidae	*Helophilus latifrons*	3	7	4	14
Syrphidae	*Helophilus obscurus*	1	1	0	2
Syrphidae	*Lapposyrphus lapponicus*	0	0	1	1
Syrphidae	*Paragus haemorrhous*	6	10	9	25
Syrphidae	*Paragus* sp.	4	13	2	19
Syrphidae	*Sphaerophoria bifurcata*	2	2	0	4
Syrphidae	*Sphaerophoria contigua*	1	3	2	6

Syrphidae	*Sphaerophoria philanthus*	8	13	4	25
Syrphidae	*Sphaerophoria* sp.	1	0	0	1
Syrphidae	*Syritta pipiens*	1	0	0	1
Syrphidae	*Syrphus vitripennis*	0	0	1	1
Syrphidae	*Toxomerus marginatus*	6	7	16	29
Syrphidae	*Trichopsomyia apisaon*	1	0	0	1
Tachinidae	*Aphria ocypterata*	5	3	1	9
Tachinidae	*Archytas californiae*	0	0	1	1
Tachinidae	*Belvosia canadensis*	1	0	0	1
Tachinidae	*Besseria brevipennis*	0	1	4	5
Tachinidae	*Chaetocrania antennalis*	0	1	0	1
Tachinidae	*Cylindromyia californica*	0	3	0	3
Tachinidae	*Cylindromyia decora*	0	1	2	3
Tachinidae	*Dinera grisescens*	1	1	0	2
Tachinidae	*Estheria* sp.	3	4	0	7
Tachinidae	*Exorista* sp.	1	0	0	1
Tachinidae	*Gonia* sp.	0	6	0	6
Tachinidae	*Gymnoclytia immaculata*	0	1	0	1
Tachinidae	*Lydina americana spcomplex*	1	0	1	2
Tachinidae	*Panzeria fasciventris*	0	1	0	1
Tachinidae	*Peleteria clara*	4	18	11	33
Tachinidae	*Peleteria* sp.	1	3	2	6
Tachinidae	*Peleteria* or *Oxydosphyria* sp.	0	3	0	3
Tachinidae	*Peleteria* or *Sphyromyia* sp.	0	0	1	1
Tachinidae	*Ptilodexia rufipennis*	1	0	4	5
Tachinidae	*Siphona medialis*	0	1	0	1
Tachinidae	*Spallanzania hebes*	1	0	0	1
Tachinidae	*Spallanzania hesperidarum*	0	2	1	3
Tachinidae	*Tachina* sp.	1	0	0	1

	Tachinidae	*Tachinidae* sp.1	1	0	0	1
	Tachinidae	*Tachinidae* sp.2	0	1	0	1
	Tachinidae	*Tachinidae* sp.3	2	0	0	2
	Tachinidae	*Tachinidae* sp.4	1	0	0	1
	Tachinidae	*Tachinidae* sp.6	1	0	0	1
	Tachinidae	*Tachinidae* sp.8	0	0	1	1
	Tachinidae	*Tachinidae* sp.9	0	1	0	1
	Therevidae	*Ozodiceromya platancala*	0	1	0	1
	Ulidiidae	sp. indet.	1	1	0	2
**Papilionoidea**						
	Hesperiidae	*Hesperia assiniboia*	2	19	55	76
	Hesperiidae	*Oarisma garita*	3	3	0	6
	Hesperiidae	*Pyrgus communis*	0	4	0	4
	Lycaenidae	*Glaucopsyche lygdamus*	1	0	0	1
	Lycaenidae	*Icaricia saepiolus*	3	4	0	7
	Lycaenidae	*Lycaena dione*	0	0	1	1
	Lycaenidae	*Plebejus melissa*	7	0	0	7
	Nymphalidae	*Cercyonis pegala*	2	4	3	9
	Nymphalidae	*Coenonympha california*	2	0	0	2
	Nymphalidae	*Phyciodes tharos*	0	2	1	3
	Nymphalidae	*Speyeria aphrodite*	0	0	1	1
	Nymphalidae	*Speyeria callippe*	1	0	0	1
	Pieridae	*Colias alexandra*	1	0	0	1
	Pieridae	*Colias philodice*	2	5	2	9
	Pieridae	*Pontia occidentalis*	1	0	1	2
**Non-Papilionoidea Lepidoptera – "Moths"**						
	Coleophoridae	*Coleophora trifolii*	1	0	0	1
	Crambidae	*Pediasia dorsipunctellus*	0	0	1	1
	Gelechiidae	sp. indet.	1	0	0	1

	Gelechiidae	*Gnorimoschema* sp.	0	0	1	1
	Geometridae	sp. indet.	1	0	0	1
	Noctuidae	*Euxoa ochrogaster*	0	0	1	1
	Noctuidae	*Euxoa* sp.	0	0	1	1
	Noctuidae	*Lacinipolia lorea*	1	2	0	3
	Noctuidae	Noctuinae sp. indet.	0	0	1	1
	Noctuidae	*Ponometia tortricina*	1	0	1	2
	Noctuidae	*Schinia villosa*	1	0	0	1
	Scythrididae	*Landryia scintillifera*	0	1	1	2
	Scythrididae	*Rhamphura ochristriata*	0	1	1	2
	Scythrididae	*Scythris eboracensis*	0	1	3	4
	Scythrididae	*Scythris inspersella*	0	0	2	2
	Tortricidae	*Hystrichophora ochreicostana*	1	0	0	1
**Formicoidea**						
	Formicidae	*Formica canadensis*	2	1	1	4
	Formicidae	*Formica lasioides*	18	9	4	31
	Formicidae	*Formica montana*	1	0	1	2
	Formicidae	*Formica neogagates*	0	5	0	5
	Formicidae	*Formica obscuripes*	13	5	0	18
	Formicidae	*Formica oreas*	1	26	0	27
	Formicidae	*Formica podzolica*	7	3	0	10
	Formicidae	*Formica ravida*	0	1	0	1
	Formicidae	*Myrmica fracticornis*	0	0	1	1
	Formicidae	*Myrmica* sp.	1	0	0	1
	Formicidae	*Tapinoma sessile*	0	4	0	4
**Hemiptera**						
	Alydidae	*Alydus* sp.	4	1	2	7
	Cicadellidae	*Cicadellidae* sp.1	1	1	1	3
	Cicadellidae	*Cicadellidae* sp.2	0	0	1	1

Miridae	*Lopidea* sp.	1	0	0	1
Miridae	*Lygus* sp.	0	0	2	2
Reduviidae	*Phymata americana*	4	5	5	14
Rhyparochromidae	sp. indet.	0	1	0	1
**Aculeata (non-Anthophila & non-Formicoidea) – "Wasps"**					
Braconidae	*Braconidae* sp.	1	1	0	2
Braconidae	*Chelonus annulipes*	1	2	0	3
Braconidae	*Chelonus* sp.	0	1	0	1
Crabronidae	*Belomicrus* sp.	0	0	3	3
Crabronidae	*Cerceris deserta*	0	1	0	1
Crabronidae	*Cerceris nigrescens*	2	0	0	2
Crabronidae	*Ectemnius arcuatus*	0	1	0	1
Crabronidae	*Ectemnius rufifemur*	1	0	5	6
Crabronidae	*Eucerceris tricolor*	1	0	0	1
Crabronidae	*Gorytes simillimus*	0	4	0	4
Crabronidae	*Nysson recticornis*	0	1	1	2
Crabronidae	*Philanthus bilunatus*	0	2	0	2
Crabronidae	*Tachysphex aequalis*	0	1	1	2
Crabronidae	*Tachysphex pompilliformis*	0	1	1	2
Cynipidae	*Diplolepsis rosae*	0	3	0	3
Ichneumonidae	*Himerta* sp.1	0	0	1	1
Ichneumonidae	*Himerta* sp.2	0	1	0	1
Ichneumonidae	sp. indet.	1	0	0	1
Ichneumonidae	*Ophion* sp.	0	0	1	1
Perilampidae	*Chrysolampus schwarzi*	0	1	0	1
Perilampidae	*Perilampus hyalinus*	0	0	3	3
Pompilidae	*Arachnospila michiganensis*	0	1	0	1
Pompilidae	*Episyron oregon*	0	0	1	1
Pompilidae	*Evagetes crassicornis*	1	0	0	1

Sphecidae	*Ammophila harti*	0	2	2	4
Sphecidae	*Prionyx atratus*	0	0	1	1
Sphecidae	*Sphex ichneumoneus*	1	0	0	1
Vespidae	*Ancistrocerus* sp.	0	1	0	1
Vespidae	*Euodynerus crypticus*	0	0	1	1
Vespidae	*Euodynerus leucomelas*	1	0	0	1
Vespidae	*Paranstrocerus* or *Stenodynerus* sp.	0	0	1	1
Vespidae	*Stenodynerus anormis*	2	2	2	6
		**586**	**726**	**502**	**1814**

Table S7. Identifications of flowering species from each distance from honey bee hives to species level.

Family	Species	100 m	500 m	5000 m	Total
Asteraceae	*Achillea millefolium*	68	183	115	366
Asteraceae	*Antennaria microphylla*	0	0	47	47
Asteraceae	*Cirsium arvense*	0	0	4	4
Asteraceae	*Cirsium undulatum*	18	21	2	41
Asteraceae	*Erigeron philadelphicus*	9	27	6	42
Asteraceae	*Erigeron speciosus*	0	0	2	2
Asteraceae	*Grindelia squarrosa*	15	346	178	539
Asteraceae	*Gutierrezia sarothrae*	0	7	0	7
Asteraceae	*Happlopappus spinulosus*	0	2	0	2
Asteraceae	*Heterotheca villosa*	117	281	47	445
Asteraceae	*Liatris punctata*	30	84	197	311
Asteraceae	*Lygodesmia juncea*	0	72	4	76
Asteraceae	*Mulgedium pulchellum*	0	1	0	1
Asteraceae	*Solidago canadensis*	96	91	47	234
Asteraceae	*Solidago missouriensis*	8	67	8	83
Asteraceae	*Sonchus arvensis*	33	34	3	70
Asteraceae	*Sonchus oleraceus*	0	0	1	1

Asteraceae	*Taraxacum officinale*	52	0	1	53
Asteraceae	*Trogopogon dubius*	1	0	4	5
Boraginaceae	*Lithospermum incisum*	28	61	0	89
Brassicaceae	*Descurainia sophia*	36	4	0	40
Brassicaceae	*Erysimum inconspicuum*	3	2	4	9
Cactaceae	*Escobaria vivipara*	1	3	4	8
Campanulaceae	*Campanula rotundifolia*	18	151	526	695
Caprifoliaceae	*Symphoricarpos occidentalis*	368	443	367	1178
Caprifoliaceae	*Symphyotrichum falcatum*	30	75	140	245
Caryophyllaceae	*Cerastium arvense*	0	9	7	16
Elaeagnaceae	*Elaeagnus commutata*	7	0	0	7
Fabaceae	*Astragalus adsurgens*	0	6	0	6
Fabaceae	*Astragalus cicer*	245	164	118	527
Fabaceae	*Astragalus flexuosus*	0	0	2	2
Fabaceae	*Astragalus striatus*	20	12	9	41
Fabaceae	*Dalea purpurea*	5	21	0	26
Fabaceae	*Glycyrrhiza lepidota*	67	497	413	977
Fabaceae	*Medicago sativa*	34	0	0	34
Fabaceae	*Melilotus albus*	66	21	0	87
Fabaceae	*Melilotus officinalis*	0	1	0	1
Fabaceae	*Thermopsis rhombifolia*	117	48	0	165
Fabaceae	*Vicia americana*	0	7	0	7
Liliaceae	*Allium textile*	68	2	0	70
Onagraceae	*Oenothera nuttallii*	0	5	1	6
Onagraceae	*Oenothera suffrutescens*	0	11	1	12
Rosaceae	*Potentilla arguta*	0	2	1	3
Rosaceae	*Potentilla concinna*	3	85	0	88
Rosaceae	*Potentilla pensylvanica*	0	1	0	1
Rosaceae	*Rosa arkansana*	14	14	10	38
Rosaceae	*Rosa woodsii*	16	8	5	29
Santalaceae	*Comandra umbellata*	81	62	0	143
		**1674**	**2931**	**2274**	**6879**

Table S8. Network size (number of interactions) for each network that was analyzed from each transect. Shaded cells denote networks that had too few species for some network metrics to be meaningful, so these replicates were excluded from analysis for the metrics: generality, vulnerability, plant and pollinator niche overlap, and nestedness. Mean network size and range in network size are shown for each dataset.

**Transect**	**Network Size**			
	Full Season All Taxa	Mid-season All Taxa	Full Season Bees Only	Without-honey bees Full Taxa
A100	118	39	67	110
A500	146	70	107	120
A5000	101	46	50	101
B100	55	22	21	48
B500	103	25	27	103
B5000	78	20	28	76
C100	164	78	98	110
C500	145	61	42	125
C5000	122	54	25	119
D100	126	79	91	57
D500	123	41	69	91
D5000	53	15	28	53
E100	18	0	7	15
E500	147	72	72	124
E5000	74	30	26	74
F100	105	46	64	74
F500	62	25	14	55
F5000	25	23	21	25
G5000	49	21	15	49
**Mean Network Size**	95.5	40.4	45.9	80.5
**Range in Network Size**	18 to 164	0 to 79	7 to 107	15 to 125

Table S9. Comparison of the full season meta-network metrics calculated for networks created by pooling data from all transects at each distance from hives, over the whole season. There was a general increase in each network metric with distance to hives, with the exceptions of plant niche overlap, functional complementarity, and generality. Bolded values indicate results that were unexpected based on the literature and/or expectations if competition between honey bees and wild pollinators is occurring. Positive (+) indicates a positive correlation between honey bee abundance and the metric, while negative (-) indicates a negative correlation, with distance from hives as a proxy for honey bee abundance (even though this was not a perfect proxy, as can be seen in Fig S1).

	Distance From Honey Bee Hives			
Network Metric	100 m	500 m	5000 m	
**Generality**	**2.29**	**2.94**	**2.06**	+
Vulnerability	9.92	12.45	14.44	-
Pollinator niche overlap	0.12	0.09	0.14	-
**Plant niche overlap**	**0.06**	**0.07**	**0.04**	+
Pollinator functional complementarity	476.78	427.39	339.66	+
Plant functional complementarity	411.43	355.71	255.55	+
**Weighted nestedness**	**4.21**	**4.15**	**5.97**	**-**
Modularity	0.59	0.58	0.63	-
**Weighted connectance**	**0.04**	**0.04**	**0.05**	**-**
Link density	6.11	7.69	8.25	-
**Interaction strength asymmetry**	**0.18**	**0.13**	**0.23**	**-**
Interaction evenness	0.54	0.60	0.61	-

Table S10. Historical data obtained from the Alberta Climate Information Service (ACIS) Verger AGCM weather station in Southern Alberta, located on the University of Alberta's Mattheis Research Ranch.

		Time Period		
	**Month**	**2018**	**2019**	**20-Year Average (1999-2019)**
Average Maximum Air Temperature (℃)	June	23.92	23.68	22.26
	July	27.20	25.87	26.62
	August	26.06	24.24	25.54
Average Precipitation (mm)	June	51.65	26.77	74.61
	July	42.78	39.39	46.49
	August	28.36	43.50	44.67

Figure S1. Abundance of honey bees caught visiting flowers (full season all taxa dataset), pooled across the full season per transect, with transects ordered by increasing honey bee abundance, and coloured by distance from bee hives. In the transect names, 100 indicates 100 m, 500 indicates 500 m, and 5000 indicates 5000 m distances from hives. The Eastern Irrigation District, owner of the land surrounding the Mattheis Research Ranch, reported no known apiaries within 19 km of the ranch's boundary line. Apiaries, commercial or otherwise, are required by law to register their hive(s) annually to the Provincial Apiculturist.

Figure S2. Visual representation of the spatial autocorrelation of residuals from the model for each response variable for the full season all taxa dataset, for which honey bee abundance was a significant predictor (before Bonferroni-Holm correction) when it was the only predictor in the model, for full season dataset. Each panel is a map of the transect locations in space (compare to Figure 2). Circles indicate the residual from each transect, with circle size proportional to residual size (smaller circle = better model fit for that transect). Colour indicates the sign of the residual; blue shows values lower than 0 and red values higher than 0. Circles close together in space having the same colour and size would indicate that spatial autocorrelation might be a problem, in which case a special correlation structure would be likely to be selected during model selection.

Figure S3. Total abundance of honey bees caught visiting flowers, with data pooled across all transects. The entire season was split into three: collection rounds 1-4 represented "early" season (May 28th-July 7th), 5-7 represented "mid" season (July 8th-July 31st), and 8-10 represented "late" season (August 1st-August 28th).

Figure S4. Rarefaction curve for interaction richness. This figure shows, for a given number of re-sampled interactions from our full season all taxa dataset (x-axis), the mean number of unique interactions detected (y-axis). There was a total observed interaction richness of 654, and Chao1 estimated 1,779 interactions (95% confidence interval: 1500-2148 interactions), meaning 31 – 44% of the estimated interactions were observed.

Figure S5. Plant-pollinator meta-networks from the full season all taxa dataset. The data from transects 100 m (upper), 500 m (mid) and 5000 m (lower) from honey bee hives are pooled across the season. The bottom row (in black) depicts the plant species (see Table S7 for the full names of plant species) and the upper row depicts the pollinator species, coloured by their given taxonomic "group" or Order. For the full pollinator species list, see Table S6. "Moths" are Lepidoptera excluding butterflies (Papilionoidea). "Wasps" are Aculeata excluding ants (Formicoidea) and bees (Anthophila). The width of each upper and lower bar represents the relative frequency of interactions observed for that species. The interactions between plants and wild pollinators are represented by blue lines; width indicates the frequency of the interaction. Honey bees and their interactions are indicated in red.

Figure1

Click here to access/download;Figure;Fig1.tif

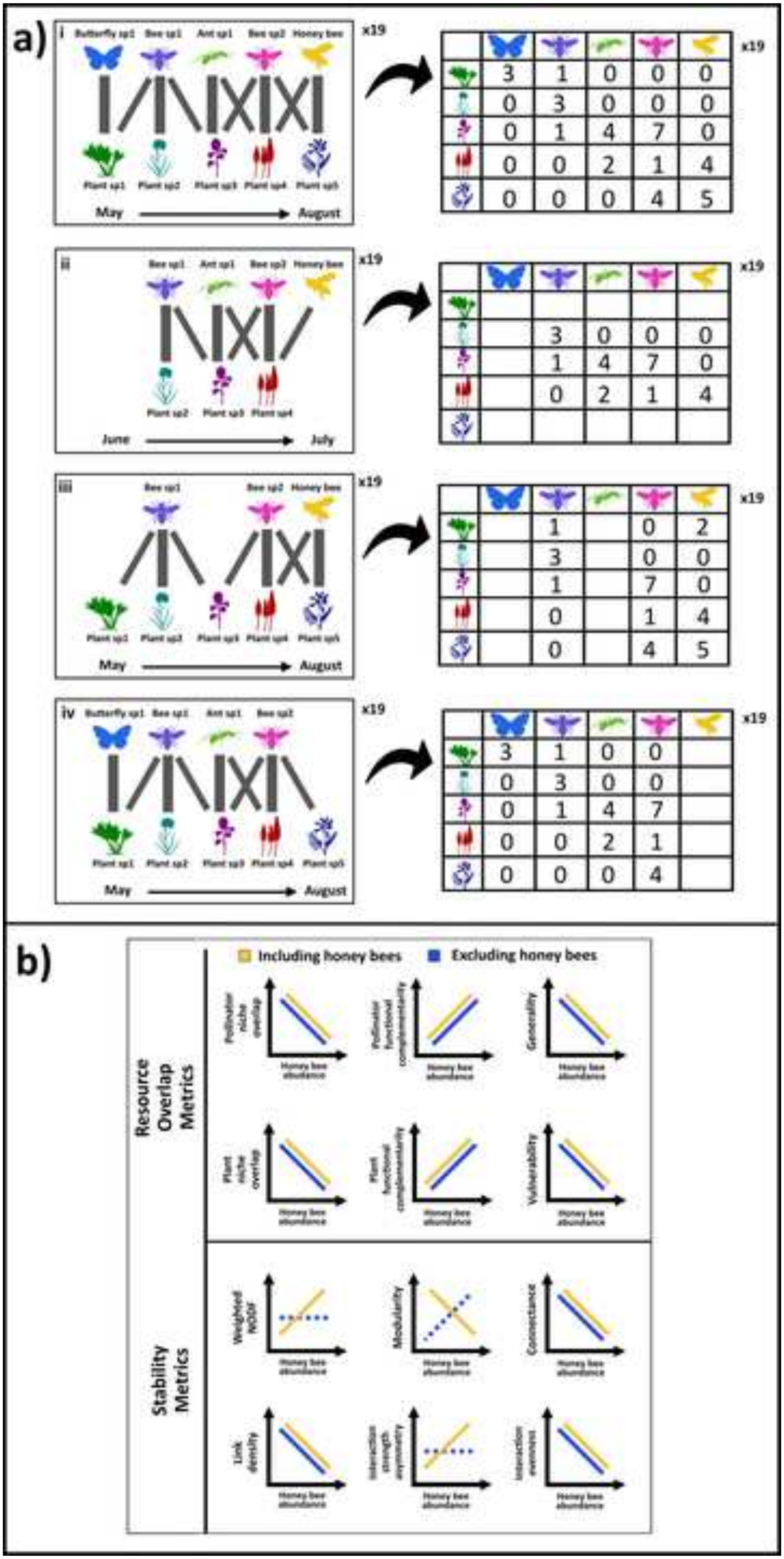

Figure2

Click here to access/download;Figure;Fig2.tif

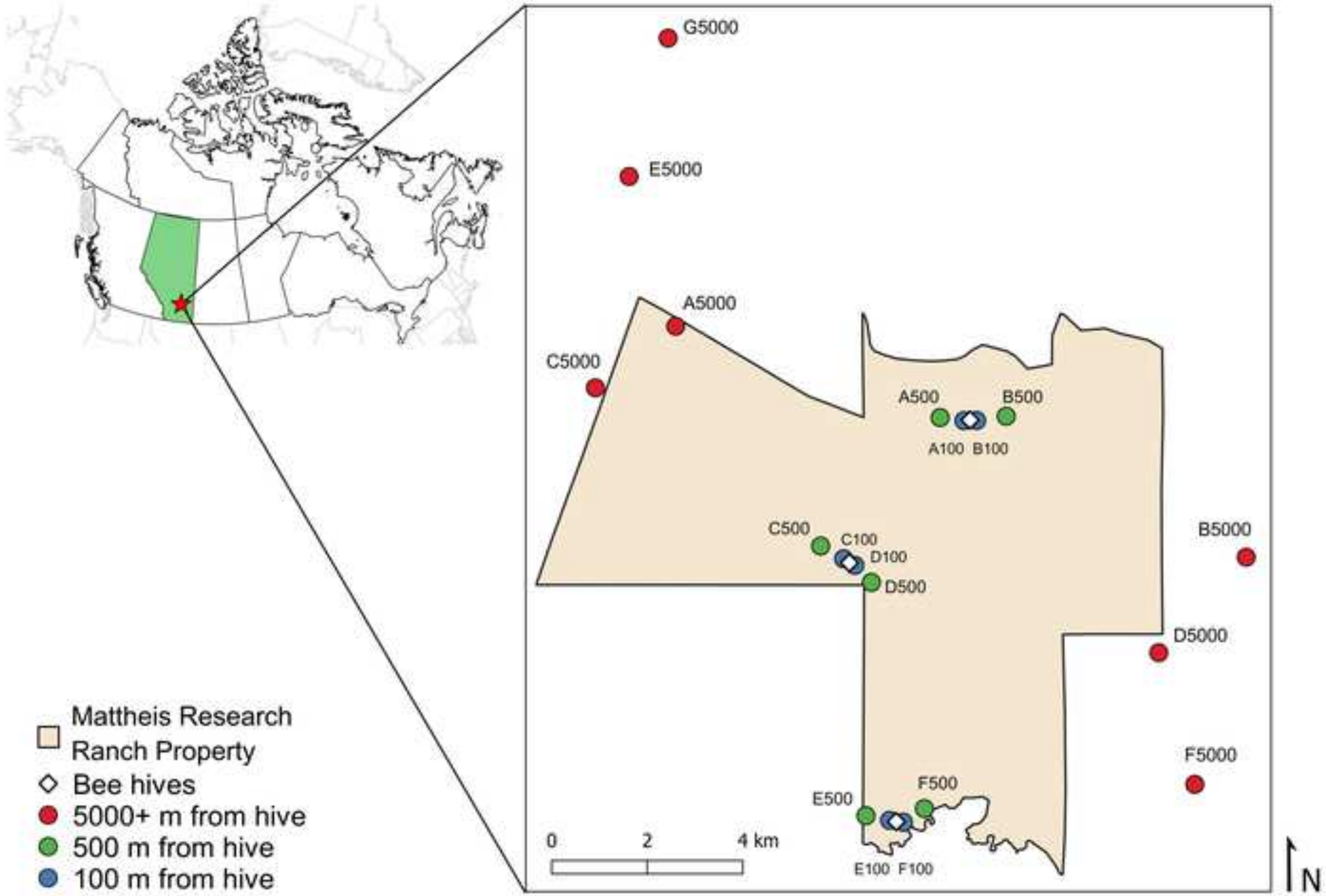

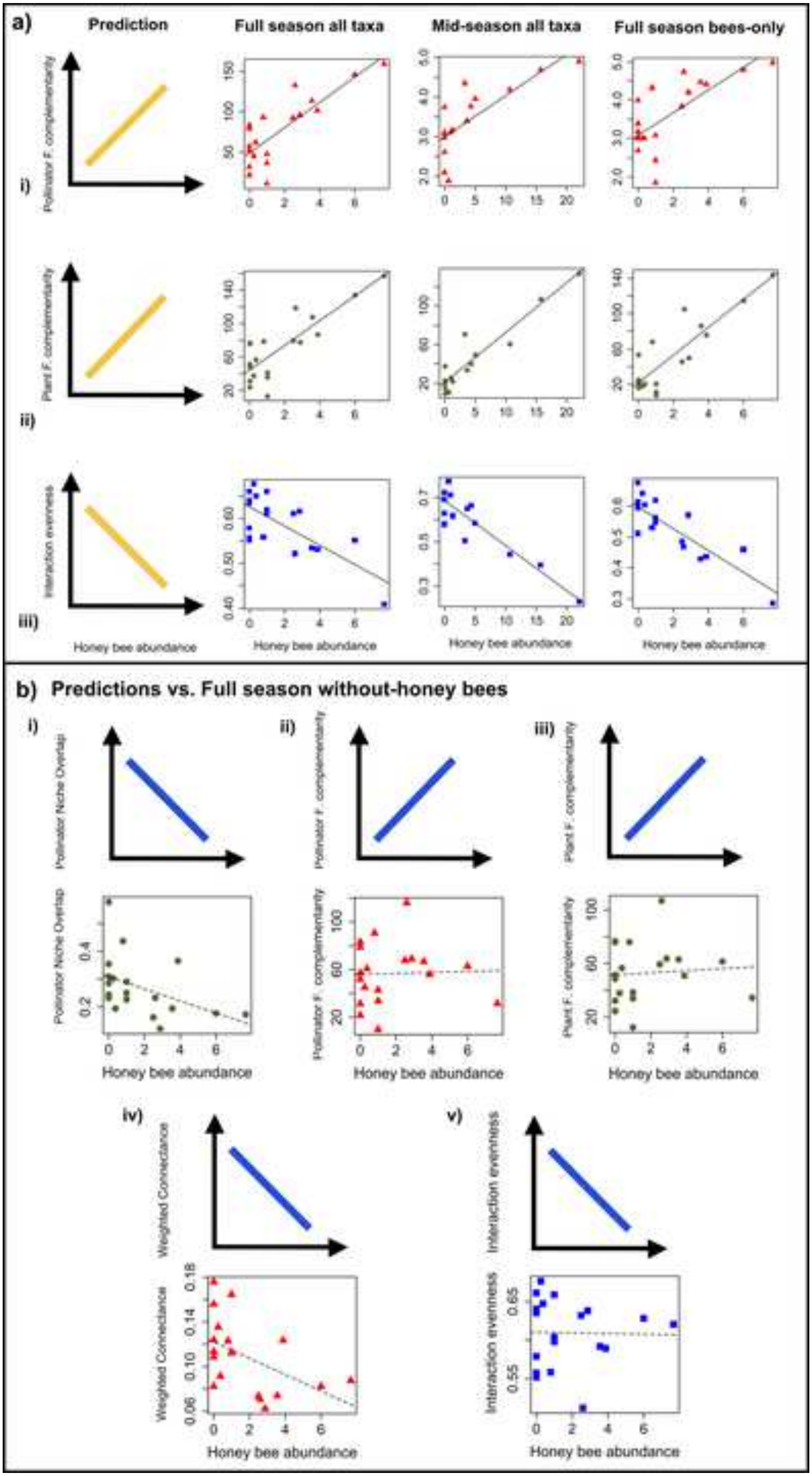

a)
Prediction
Full season all taxa
Mid-season all taxa
Full season bees-only
i)
Pollinator F. complementarity
ii)
Plant F. complementarity
iii)
Interaction evenness
Honey bee abundance
b) Predictions vs. Full season without-honey bees
i)
Pollinator Niche Overlap
ii)
Pollinator F. complementarity
iii)
Plant F. complementarity
iv)
Weighted Connectance
v)
Interaction evenness

Click here to access/download;Figure;FigS1.tif

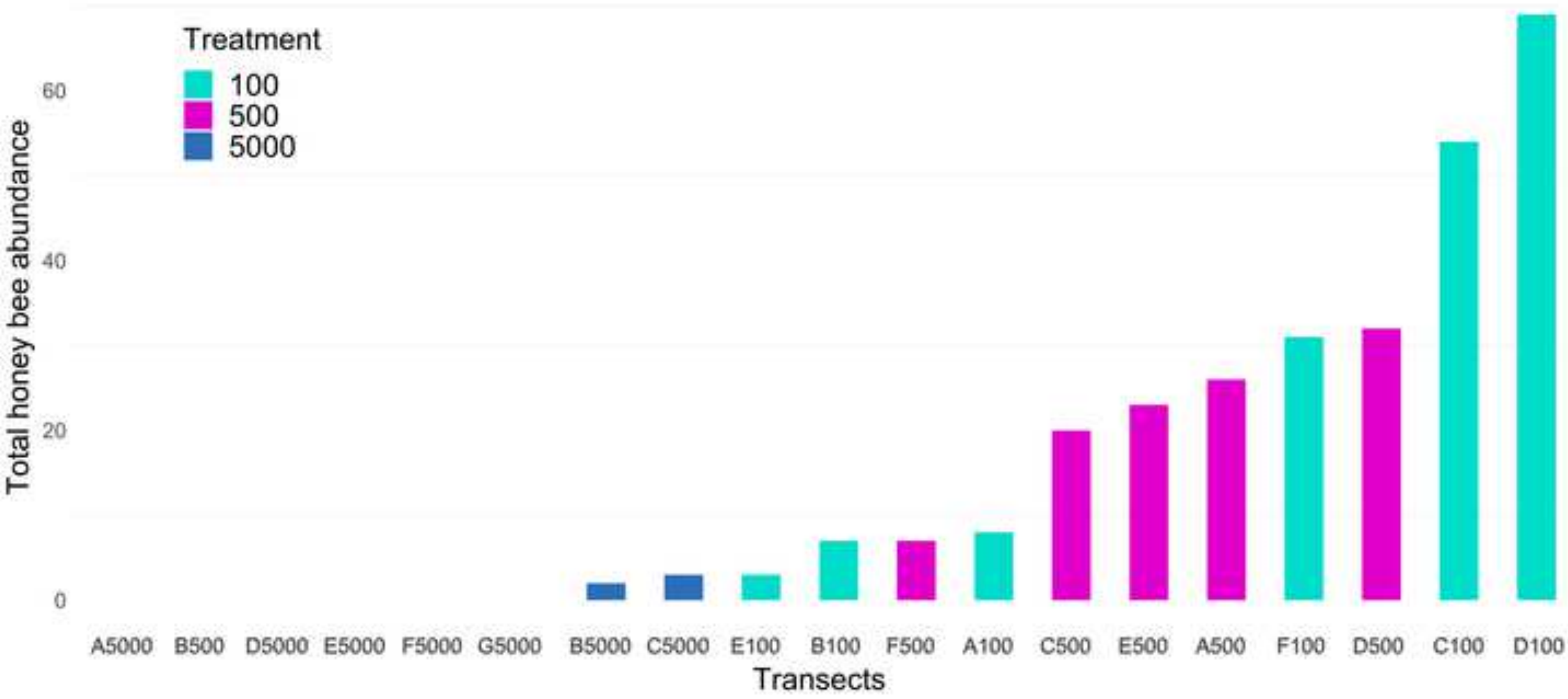

FigureS2

Click here to access/download;Figure;FigS2.tif

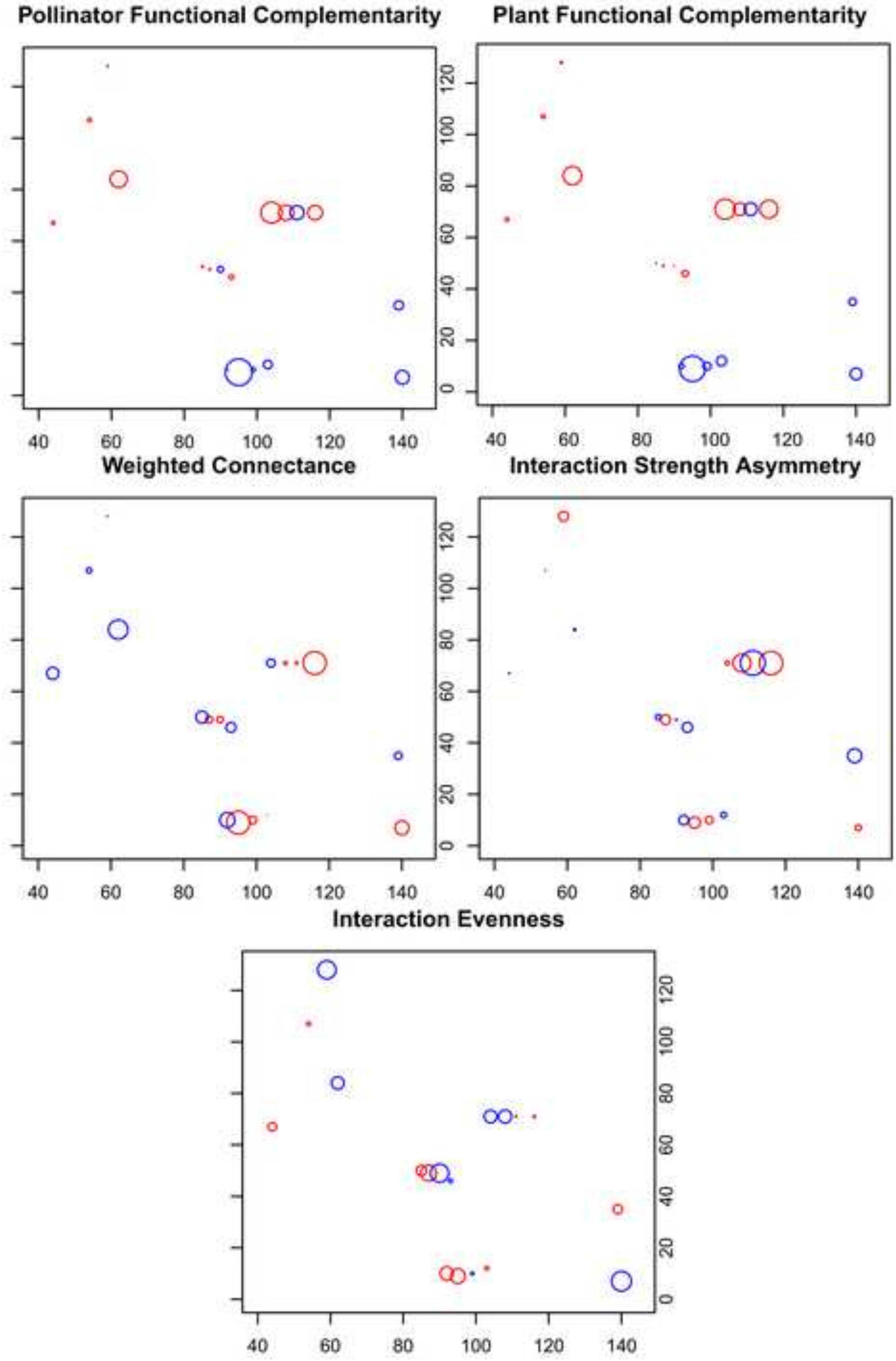

FigureS3

Click here to access/download;Figure;FigS3.tif

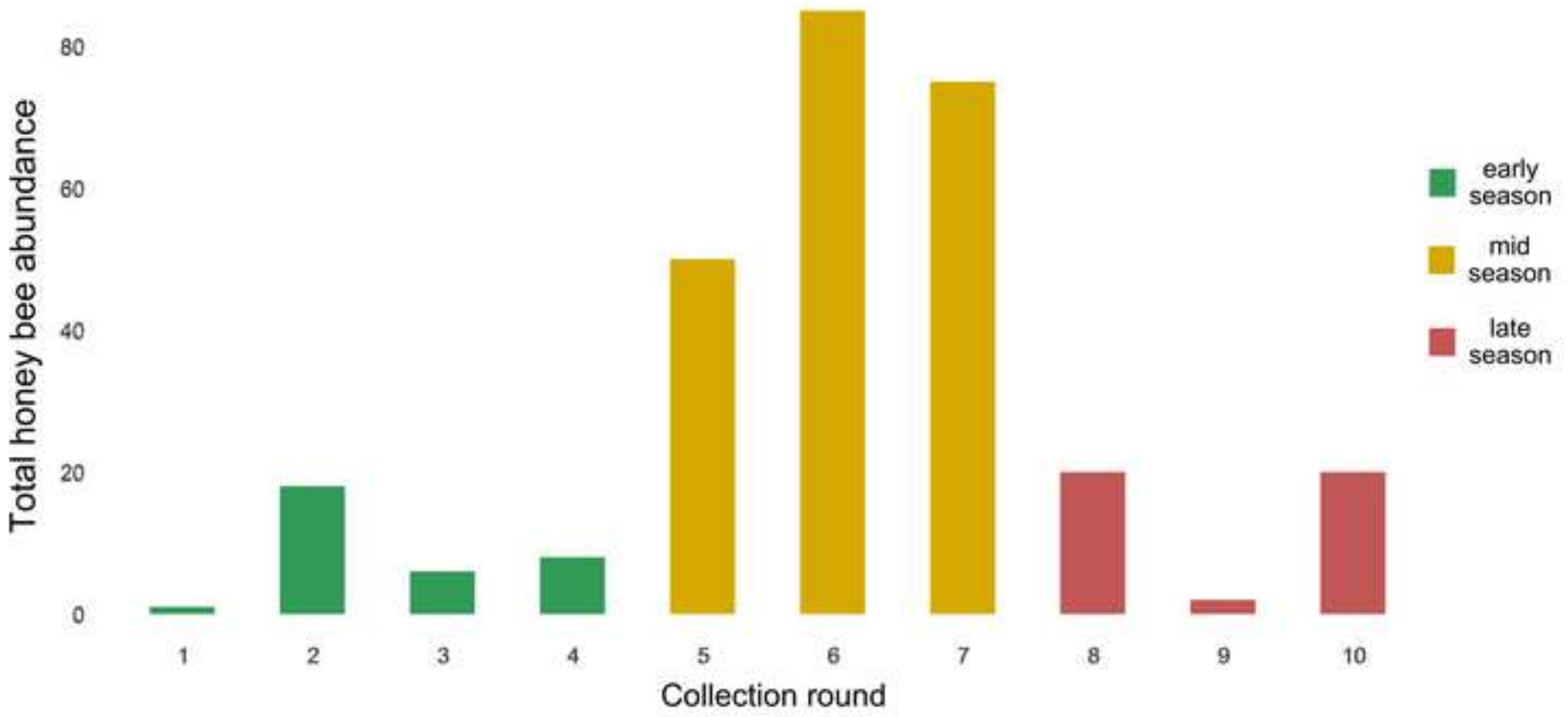

FigureS4

Click here to access/download;Figure;FigS4.tif

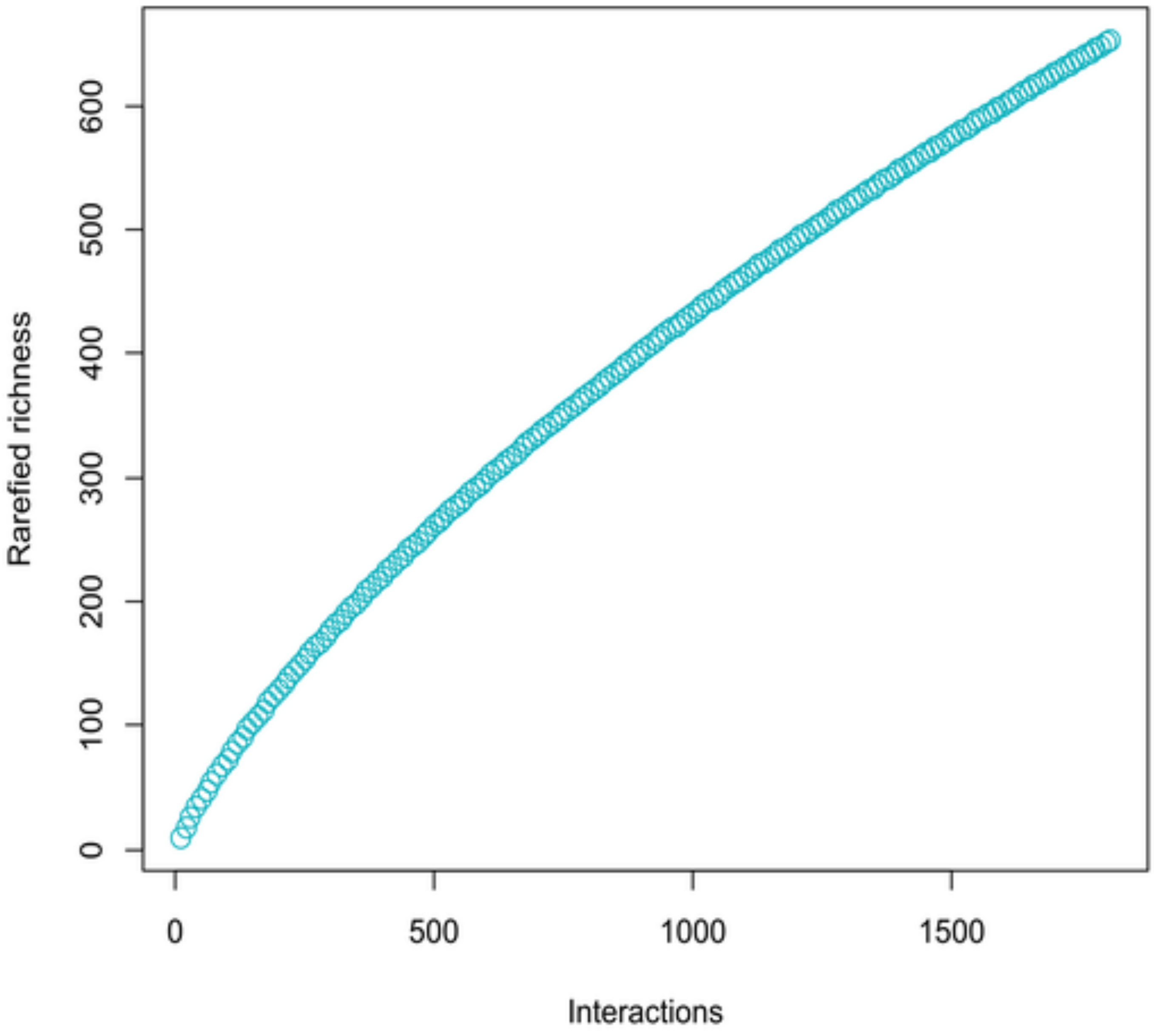

FigureS5

Click here to access/download;Figure;FigS5.tif

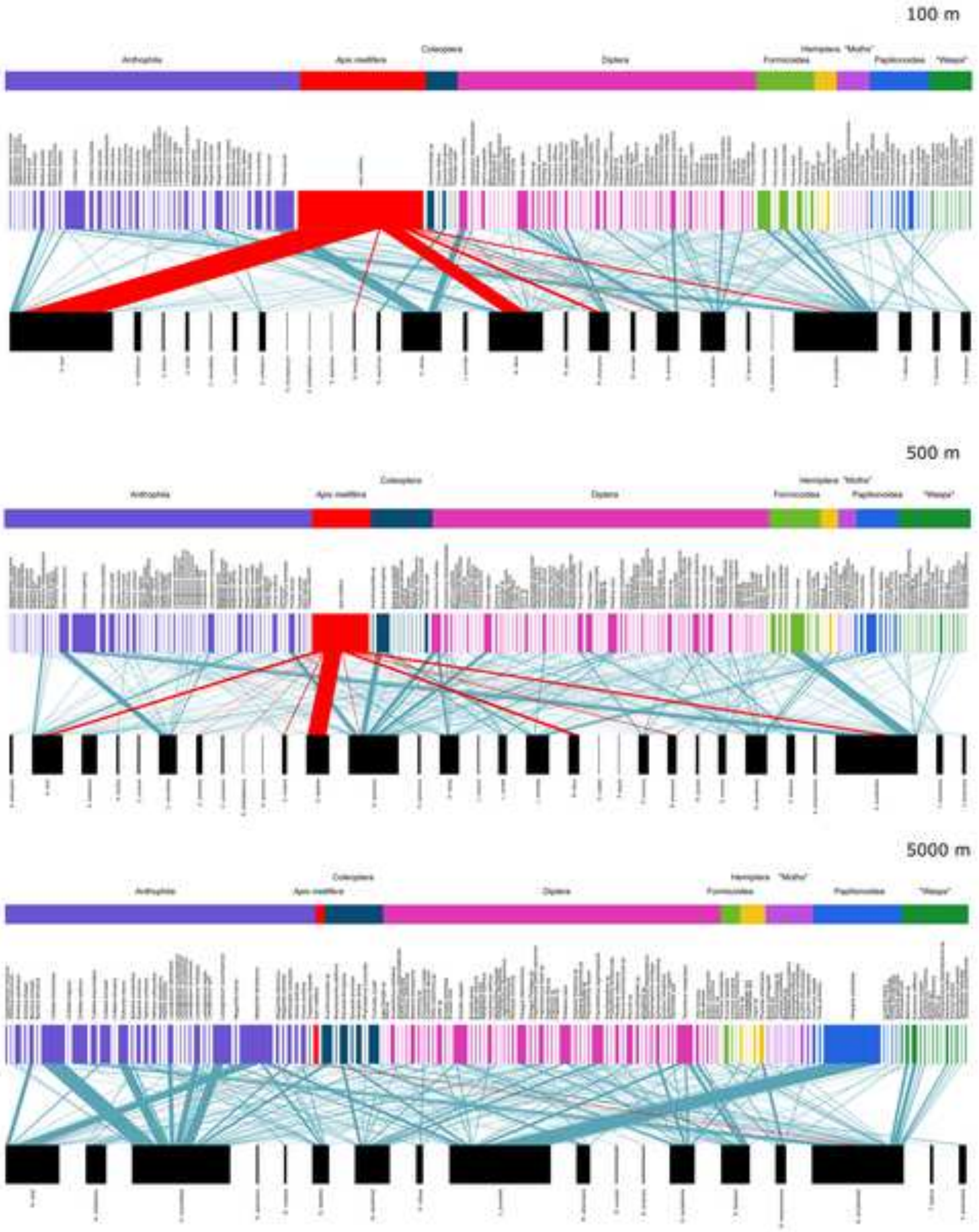

Supplement: Attachment — Submitted filename: PONE-D-23-06336_reviewer PF.pdf [file pone.0287332.s016.pdf]
